# Supplementary material for: Efficacy and safety of passive immunotherapies targeting amyloid beta in Alzheimer’s disease: A systematic review and meta-analysis
Source: PLoS Med. 2025 Mar 31;22(3):e1004568. doi: 10.1371/journal.pmed.1004568 (PMC12002640; doi:10.1371/journal.pmed.1004568)
Supplement: S1 File — Appendix A. Preferred Reporting Items for Systematic review and Meta-Analysis Protocols (PRISMA-P) 2015 checklist: recommended items to address in a systematic review protocol. Text A. Search strategy. Text B. Data extraction for continuous outcomes. Text C. Methodologies to combine groups. Text D. Calculation methods for the standard mean difference (SMD). Text E. Calculation of number needed to treat/harm (NNT)/(NNH). Text F. Comparison between meta and metafor packages. Text G. Definition of serious adverse events (SAE). Text H. Definition of cerebral macrohemorrhage. Table A. Trials that were included in the analyses. Table B. Results of meta-regression analyses for death, fall, and dizziness. Table C. Summary of amyloid PET and Tau PET results. Table D. Summary of biomarker results. Table E. Number needed to treat/harm. (DOCX) [file pmed.1004568.s001.docx]

Supplemental Materials

Table of Contents

[Supplemental Appendix 2](#_Toc190169794)

[Appendix A. PRISMA-P (Preferred Reporting Items for Systematic review and Meta-Analysis Protocols) 2015 checklist: recommended items to address in a systematic review protocol 2](#_Toc190169795)

[Supplemental Texts 6](#_Toc190169796)

[Text A. Search strategy 6](#_Toc190169797)

[Text B. Data extraction for continuous outcomes 8](#_Toc190169798)

[Text C. Methodologies to combine groups 11](#_Toc190169799)

[Text D. Calculation Methods for the standard mean difference (SMD) 15](#_Toc190169800)

[Text E. Calculation of number needed to treat/ harm (NNT)/(NNH) 16](#_Toc190169801)

[Text F. Comparison between meta and metafor packages 21](#_Toc190169802)

[Text G. Definition of serious adverse events (SAE) 24](#_Toc190169803)

[Text H. Definition of cerebral macrohemorrhage 30](#_Toc190169804)

[Supplemental Tables 31](#_Toc190169805)

[Table A. Trials that were included in the analyses 31](#_Toc190169806)

[Table B. Results of meta-regression analyses for death, fall, and dizziness. 32](#_Toc190169807)

[Table C. Summary of amyloid PET 35](#_Toc190169808)

[Table D. Summary of tau PET results 39](#_Toc190169809)

[Table E. Summary of cerebrospinal fluid biomarker results 41](#_Toc190169810)

[Table F. Summary of plasma biomarker results 44](#_Toc190169811)

[Table G. Number needed to treat/harm 48](#_Toc190169812)

**Supplemental Appendix**

**Appendix A. PRISMA-P (Preferred Reporting Items for Systematic review and Meta-Analysis Protocols) 2015 checklist: recommended items to address in a systematic review protocol**

| Section and topic | Item No | Checklist item |  |  | |  | | |  |
| --- | --- | --- | --- | --- | --- | --- | --- | --- | --- |
| ADMINISTRATIVE INFORMATION | | | |  | Where it was mentioned. | |  | | |
| Title: |  |  | |  |  | | |  |  |
| Identification | 1a | Identify the report as a protocol of a systematic review | | 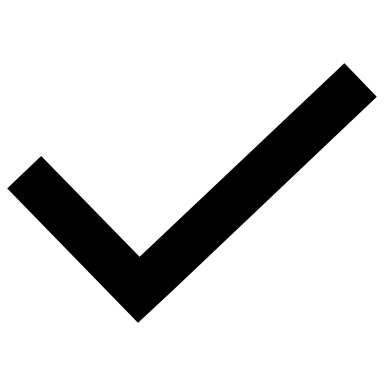 | Title | | |  |  |
| Update | 1b | If the protocol is for an update of a previous systematic review, identify as such | | NA |  | | |  |  |
| Registration | 2 | If registered, provide the name of the registry (such as PROSPERO) and registration number | | NA |  | | |  |  |
| Authors: |  |  | |  |  | | |  |  |
| Contact | 3a | Provide name, institutional affiliation, e-mail address of all protocol authors; provide physical mailing address of corresponding author | | 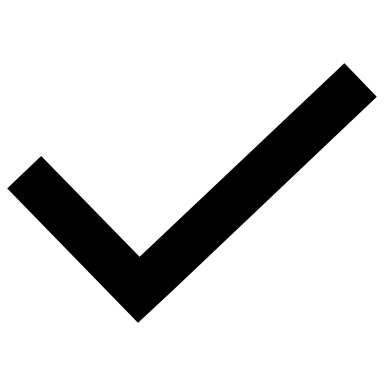 | Author information | | |  |  |
| Contributions | 3b | Describe contributions of protocol authors and identify the guarantor of the review | | 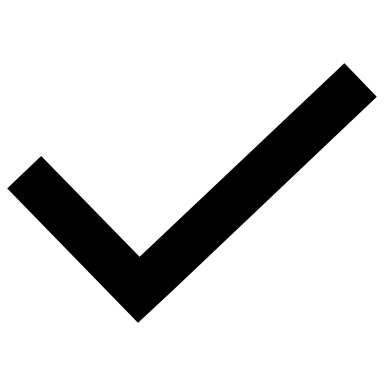 | Author contribution section | | |  |  |
| Amendments | 4 | If the protocol represents an amendment of a previously completed or published protocol, identify as such and list changes; otherwise, state plan for documenting important protocol amendments | | NA |  | | |  |  |
| Support: |  |  | |  |  | | |  |  |
| Sources | 5a | Indicate sources of financial or other support for the review | | 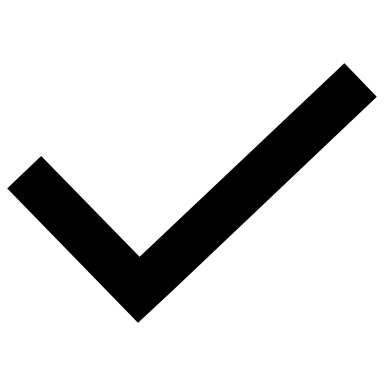 | “Financial Disclosure Statement” section in the Acknowledgement | | |  |  |
| Sponsor | 5b | Provide name for the review funder and/or sponsor | | 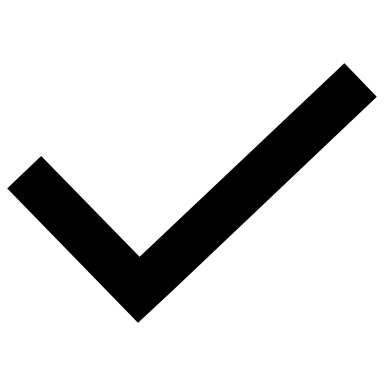 | “Financial Disclosure Statement” section in the Acknowledgement | | |  |  |
| Role of sponsor or funder | 5c | Describe roles of funder(s), sponsor(s), and/or institution(s), if any, in developing the protocol | | 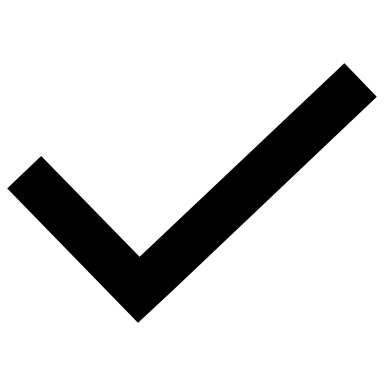 | “Financial Disclosure Statement” section in the Acknowledgement | | |  |  |
| INTRODUCTION | | | |  |  | | |  |  |
| Rationale | 6 | Describe the rationale for the review in the context of what is already known | | 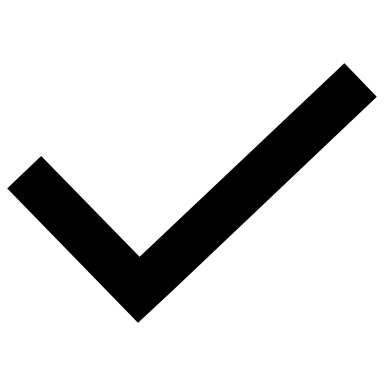 | In the introduction, “The main concern surrounding these drugs relates to…Thus, there is a growing interest in meta-regression analyses to address the impact of using different antibodies on efficacy and safety profiles.”. | | |  |  |
| Objectives | 7 | Provide an explicit statement of the question(s) the review will address with reference to participants, interventions, comparators, and outcomes (PICO) | | 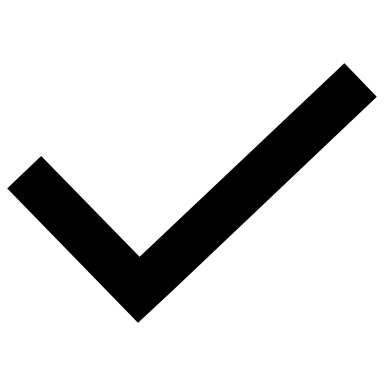 | In the introduction, we stated “We conducted an updated systematic review including meta-regression analyses to investigate the efficacy and safety of mAbs against Ab for AD. | | |  |  |
| METHODS | | | |  |  | | |  |  |
| Eligibility criteria | 8 | Specify the study characteristics (such as PICO, study design, setting, time frame) and report characteristics (such as years considered, language, publication status) to be used as criteria for eligibility for the review | | 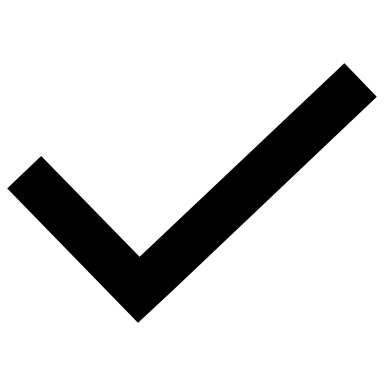 | The first and the second paragraphs of the “Search strategy and selection criteria” section in the methods. | | |  |  |
| Information sources | 9 | Describe all intended information sources (such as electronic databases, contact with study authors, trial registers or other grey literature sources) with planned dates of coverage | | 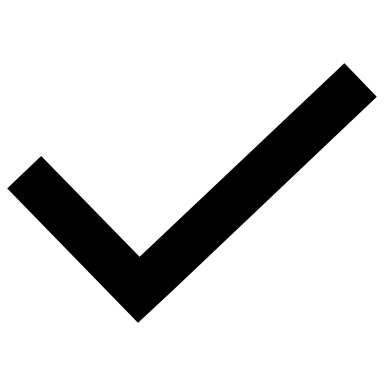 | The first paragraph of the “Search strategy and selection criteria” section in the methods. “We searched for published trials on Pubmed, Embase, and Clinical Trials.gov on January 14^th^ 2024.” | | |  |  |
| Search strategy | 10 | Present draft of search strategy to be used for at least one electronic database, including planned limits, such that it could be repeated | | 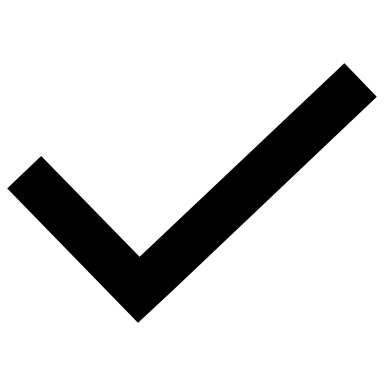 | The first paragraph of the “Search strategy and selection criteria” section in the method and the **Text A in the supporting information.** | | |  |  |
| Study records: |  |  | |  |  | | |  |  |
| Data management | 11a | Describe the mechanism(s) that will be used to manage records and data throughout the review | | 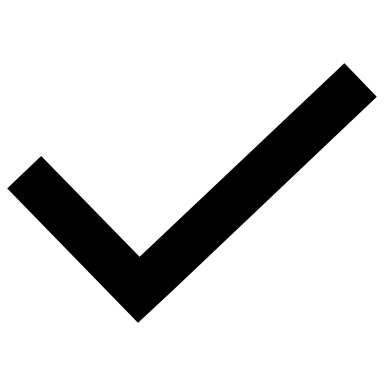 | **Text A in the supporting information.** | | |  |  |
| Selection process | 11b | State the process that will be used for selecting studies (such as two independent reviewers) through each phase of the review (that is, screening, eligibility and inclusion in meta-analysis) | | 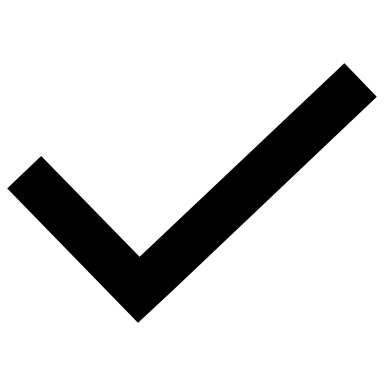 | In the first paragraph of the “Search strategy and selection criteria” section in the methods, we stated “Study selection, review, and data extraction were performed by two authors independently, and any disagreements were resolved by consensus.”. | | |  |  |
| Data collection process | 11c | Describe planned method of extracting data from reports (such as piloting forms, done independently, in duplicate), any processes for obtaining and confirming data from investigators | | 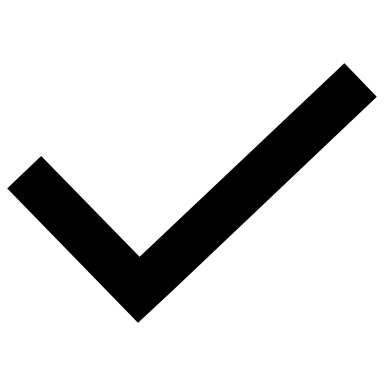 | In the first paragraph of the “Search strategy and selection criteria” section in the methods, we stated “Study selection, review, and data extraction were performed by two authors independently, and any disagreements were resolved by consensus.”. | | |  |  |
| Data items | 12 | List and define all variables for which data will be sought (such as PICO items, funding sources), any pre-planned data assumptions and simplifications | | 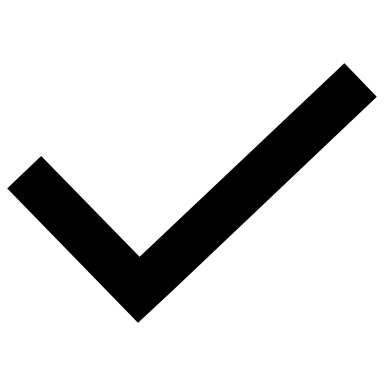 | “Outcomes and modifiers” section in the method. | | |  |  |
| Outcomes and prioritization | 13 | List and define all outcomes for which data will be sought, including prioritization of main and additional outcomes, with rationale | | 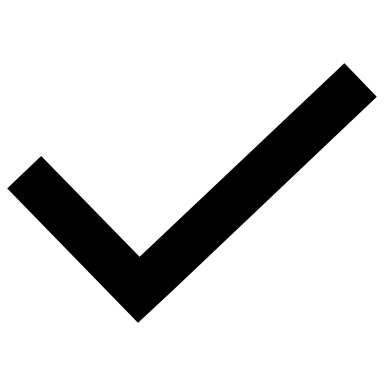 | In the first paragraph of the “Outcomes and modifiers” section in the method, all outcomes were listed with the information regarding prioritization. Also, detailed definition of serious adverse events and cerebral macrohemorrhage are described in **Text G and Text H in the supporting information.** | | |  |  |
| Risk of bias in individual studies | 14 | Describe anticipated methods for assessing risk of bias of individual studies, including whether this will be done at the outcome or study level, or both; state how this information will be used in data synthesis | | 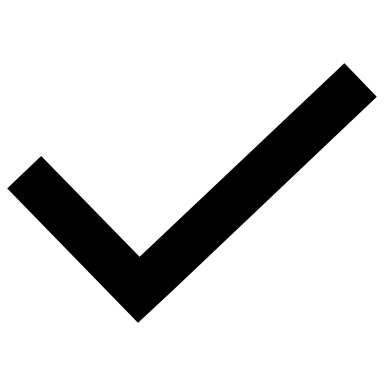 | In the first paragraph of the “Data analysis” section in the method.  We stated “Publication bias was assessed using Egger’s test and Funnel’s plot for each outcome reported in at least 10 trials. P-value of <0.05 for Egger’s test was considered statistically significant.”. | | |  |  |
| Data synthesis | 15a | Describe criteria under which study data will be quantitatively synthesised | | 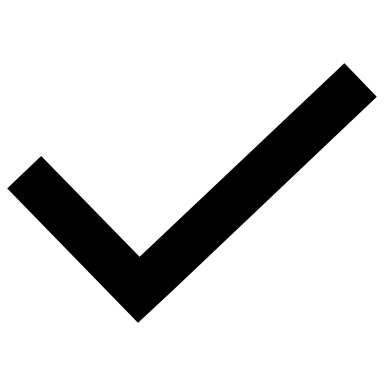 | In the second paragraph of the “Data analysis” section in the method.  When data were available for 10 or more trials, meta-regression was performed to evaluate the impact of modifiers. | | |  |  |
|  | 15b | If data are appropriate for quantitative synthesis, describe planned summary measures, methods of handling data and methods of combining data from studies, including any planned exploration of consistency (such as I^2^, Kendall’s τ) | | 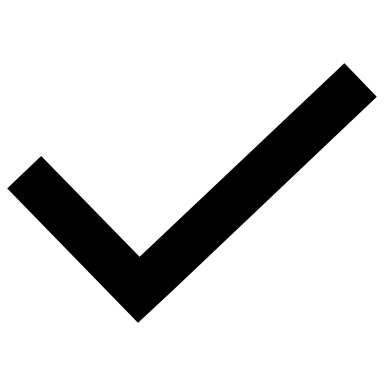 | In the second paragraph of the “Data analysis” section in the method.  Summary measure of CDR-SB was expressed as Mean Difference (MD), while Standardized Mean Difference (SMD) was expressed for ADAS-Cog, as it was reported in either ADAS-Cog version 11, 13, or 14 in each trial. To assess heterogeneity, t2 and I2 statistics were reported, and Cochran’s Q test was performed, where a P-value of <0.05 was considered statistically significant. The heterogeneity was characterized as low, moderate, or substantial, based on I2 cut-offs of 25%, 50%, and 75%, respectively. t2 was calculated using the Paule-Mandel method to estimate between-study variance. | | |  |  |
|  | 15c | Describe any proposed additional analyses (such as sensitivity or subgroup analyses, meta-regression) | | 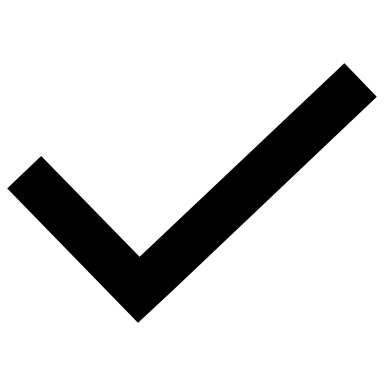 | Meta-regression analyses and sensitivity analyses were described in the second and the third paragraphs of the “Data analysis” section in the method. | | |  |  |
|  | 15d | If quantitative synthesis is not appropriate, describe the type of summary planned | | 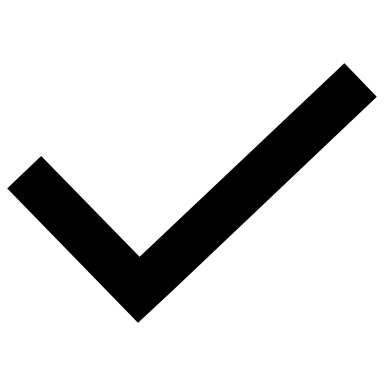 | In the third paragraph of the “Outcome and modifiers” section in the method, plan for the summary of the neuroimaging and biomarker results are described. | | |  |  |
| Meta-bias(es) | 16 | Specify any planned assessment of meta-bias(es) (such as publication bias across studies, selective reporting within studies) | | 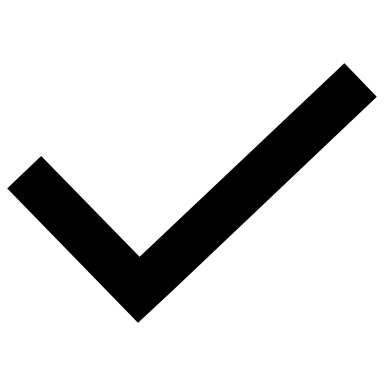 | In the first paragraph of the “Data analysis” section in the method. Plan for the Egger’s test and Funnel’s plot are described. | | |  |  |
| Confidence in cumulative evidence | 17 | Describe how the strength of the body of evidence will be assessed (such as GRADE) | | 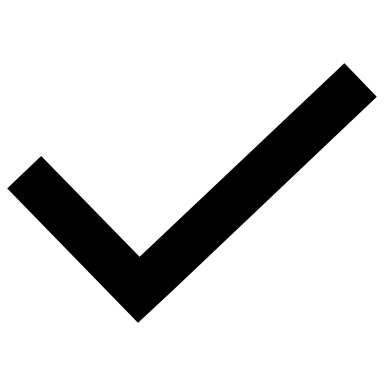 | In the second paragraph of the “Search strategy and selection criteria” section in the method. Jaded scale was used to ensure the quality of trial reports. | | |  |  |

*From: Shamseer L, Moher D, Clarke M, Ghersi D, Liberati A, Petticrew M, Shekelle P, Stewart L, PRISMA-P Group. Preferred reporting items for systematic review and meta-analysis protocols (PRISMA-P) 2015: elaboration and explanation. BMJ. 2015 Jan 2;349(jan02 1):g7647.*

# **Supplemental Texts**

## **Text A. Search strategy**

***Pubmed***

((Alzheimer) OR (mild cognitive impairment) OR (dementia)) AND ((antibody) OR (passive immunotherapy)) AND ((randomized controlled trial) OR (randomized clinical trial))

***Embase (via Ovid)***

(“Alzheimer.mp” or “mild cognitive impairment” or “dementia.mp”) and (“Antibody. mp. or antibody” or “passive immunotherapy”) and (“randomized controlled trial” or “randomized clinical trial”)

“.mp.” is a filed code in Embase and stands for “multiple fields”. It indicates that the search should look for the keyword in multiple fields of the database records, such as the title or the abstract.

***Clinical Trials.gov***

Search was performed with the following terms.

Condition/disease: Alzheimer

Intervention/Treatment: Placebo

Phase: Phase 3

Study type: Interventional

The search results from PubMed, Embase, and ClinicalTrials.gov were downloaded in CSV format and distributed to two authors. Study selection, review, and data extraction were performed independently by both authors, and any disagreements were resolved by consensus.

## **Text B. Data extraction for continuous outcomes**

The reporting methods for continuous outcome variables varied between trials, so the parameters reported in each trial were appropriately recalculated as described below. Ultimately, the difference in mean change between the experimental group and the control group, as well as the standard error of the difference, were calculated. A generic inverse meta-analysis was then performed using the “metagen” function from the R package “meta”.

1. **Method for deriving the difference in mean change**

We used the reported value directly if the difference in mean change between the experimental and control groups and its SE were provided in the paper. This is because in many trials, covariate-adjusted differences, such as the leas squared mean change, are reported, and using these values allows for a more accurate assessment. Only if the difference is not reported, we calculated using the following formula.

- 1. **Mean change in each group**

Mean change in the experimental and the control groups were obtained by subtracting the baseline mean from the follow-up mean.

$$M_{e}={Followup mean}_{e}-{Baseline mean}_{e}$$

$$M_{c}={Followup mean}_{c}-{Baseline mean}_{c}$$

Where:

- Me, Mc = Mean change in the experimental and the control group
- Followup mean_e_, Followup mean_e_ = Mean value of the outcome at the follow-up in the experimental and the control group.
- Baseline mean_e_, Baseline mean_e_ =Mean value of the outcome at the baseline in the experimental and the control group.
  1. **Difference in mean change**

$$Diff=M_{e}-M_{c}$$

Where:

- Diff= Difference in mean change

If the paper reports the difference in mean change between the experimental and the we could use that value.

1. **Method for deriving the standard error (SE) of the difference in mean change**
   1. **Calculation from the 95% confidence interval (CI) of the difference in mean change**

$${SE}_{diff}=\frac{{Upper limit of CI}_{diff}-{Lower limit of CI}_{diff}}{2\times1.96}$$

Where:

- SE_diff_ = the standard error of the difference in mean change
- Upper limit of CI_diff_=the upper limit of the 95% CI of the difference in mean change
- Lower limit of CI_diff_= the lower limit of the 95% CI of the difference in mean change
  1. **Calculation from the standard deviation (SD) of the mean change in each group**

If the 95%CI, SD, or SE of the difference in mean change is not reported, we calculated the SE of the difference of mean change using the SE or SD of the mean change in each group. This formula is explained in the chapter 7 of “Essential Medical Statistics”. (Reference No.16)

$${SE}_{diff}=\sqrt{\frac{{SD}_{e}^{2}}{N_{e}}+\frac{{SD}_{c}^{2}}{N_{c}}} =\sqrt{{SE}_{e}^{2}+{SE}_{c}^{2}}$$

Where:

- SE_diff_ = the standard error of the difference in mean change
- SE_e_, SE_c_= the standard error of the mean change in the experimental and the control groups
- SD_e_, SD_c_ = the standard deviation of the mean change in the experimental and the control groups
- N_e_, N_c_= Sample size of the experimental and the control groups

## **Text C. Methodologies to combine groups**

**Continuous outcomes**

In the main analysis, if the experimental arm had a single dose, the results were used as is. However, cases with multiple dosage groups in antibody arms were combined. The combined sample size, mean change, standard deviation, difference in mean change, and standard error of the difference were calculated using the following formulas.

1. **Combined sample size**

$$N_{combined}=N_{1}+N_{2}$$

Where:

- N_combined_ =the combined sample size for both groups.
- N_1_, N_2_ =the sample size of the low dose and high dose groups, respectively

1. **Combined Mean Change**

$$M_{combined}=\frac{N_{1}\cdot M_{1}+N_{2}\cdot M_{2}}{N_{1}+N_{2}}$$

　　　 Where:

- M_1_, M_2_ =the mean change of the low dose and high dose groups, respectively

1. **Combined Standard Deviation (SD)**

$${SD}_{combined}=\sqrt{\frac{\left( N_{1}-1 \right)\cdot{SD}_{1}^{2}+\left( N_{2}-1 \right)\cdot{SD}_{2}^{2}+\frac{N_{1}\cdot N_{2}}{N_{1}+N_{2}}\cdot{(M_{1}-M_{2})}^{2}}{N_{1}+N_{2}-1}}$$

Where:

- SD_combined_=the combined standard deviation for both groups
- SD_1_, SD_2_ =the dose-specific standard deviations.

1. **Difference in Mean Change**

$$\mathrm{Diff}_{\mathrm{combined}}=M_{combined}-M_{control}$$

Where:

- Diff_combined_= the difference in mean change between the combined dose group and the control group
- M_control_= the mean change for the control group

1. **Standard Error of the Difference**

$${SE}_{diff}=\sqrt{\frac{{SD}_{combined}^{2}+{SD}_{control}^{2}}{N_{combined}+N_{control}}}$$

Where:

- SE_diff_ = the standard error of the difference in mean change
- SD_control_ = the standard deviation of the control group
- N_control_ = sample size of the control group

For the aducanumab study, SD of the low and the high dose groups were calculated based on the following formulas.

$${{SE}_{diff}^{2}=SE}_{e}^{2}+{SE}_{c}^{2}$$

$${SE}_{e}^{2}={SE}_{diff}^{2}-{SE}_{c}^{2}$$

$${SE}_{e}=\sqrt{{SE}_{diff}^{2}-{SE}_{c}^{2}}=\sqrt{\left( \frac{{Upper limit of CI}_{diff}-{Lower limit of CI}_{diff}}{2\times1.96} \right)^{2}-{SE}_{c}^{2}}$$

Example:

For the Noncarriers study of bapineuzumab (Salloway S et.al., NEJM 2014), there were two dose groups of bapineuzumab (0.5mg/kg and 1.0 mg/kg). N_combined_ (the combined sample size of these groups), M_combined_ (the mean change in CDR-SB for the combined group), and SD_combined_ (the standard deviation of the change in CDR-SB for the combined group) were calculated.

$$N_{combined}=N_{1}+N_{2}=314+307=621$$

$$M_{combined}=\frac{N_{1}\cdot M_{1}+N_{2}\cdot M_{2}}{N_{1}+N_{2}}= \frac{314 \times2.6 +307 \times2.8}{314 +307}=2.70$$

$${SD}_{combined}=\sqrt{\frac{\left( N_{1}-1 \right)\cdot\mathrm{SD}_{1}^{2}+\left( N_{2}-1 \right)\cdot\mathrm{SD}_{2}^{2}+\frac{N_{1}\cdot N_{2}}{N_{1}+N_{2}}\cdot\left( M_{1}-M_{2} \right)^{2}}{N_{1}+N_{2}-1}} =\sqrt{\frac{(314-1)\times({0.2\times\sqrt{314})}^{2}+(307-1)\times({0.2\times\sqrt{307})}^{2}+\frac{314\times307}{314+307} \times{(2.6-2.7)}^{2}}{314+307-1}}=3.52$$

SD was calculated as $SE\times\sqrt{N}$.

The values were taken from Table 1 and Table 2 of the paper (Salloway S et.al., NEJM 2014).

**Categorical outcomes**

For the categorical outcomes, sample size (N_1_, N_2_) and the number of events (E_1_, E_2_) for each group was added.

1. **Combined sample size**

$$N_{combined}=N_{1}+N_{2}$$

Where:

- N_combined_ =the combined sample size for both groups.
- N_1_, N_2_ =the sample size of the low dose and high dose groups, respectively

1. **Combined number of events**

$$E_{combined}=E_{1}+E_{2}$$

Where:

- E_combined_ =the combined number of events.
- E_1_, E_2_ =the number of events for the low dose and high dose groups, respectively

Example:

For the Noncarriers study of bapineuzumab (Salloway S et.al., NEJM 2014), there were two dose groups of bapineuzumab (0.5mg/kg and 1.0 mg/kg). N_combined_ (the combined sample size of these groups), E_combined_ (the combined number of ARIAE events) were calculated.

$$N_{combined}=N_{1}+N_{2}=337 +329=666$$

$$E_{combined}=E_{1}+E_{2} =14+31=45$$

The values were taken from and Table 3 of the paper (Salloway S et.al., NEJM 2014).

## **Text D. Calculation Methods for the standard mean difference (SMD)**

The SMD was calculated with the following formula.

Cohen’s *d* refers to the SMD that was estimated from study-specific estimates of the respective outcome means and variances. Let $\bar{x}_{E}^{k}$ and $\bar{x}_{C}^{k}$ respectively represent estimates of the mean outcome level (e.g., CDR-SB, ADAS-COG) in the experimental and control groups in the $k$th study. The standardized mean treatment effect was estimated as

$$d_{k}=\frac{\bar{x}_{E}^{k}-\bar{x}_{C}^{k}}{S_{k}},$$

where $S^{2}$ is an estimated pooled variance, calculated as

$$S_{k}^{2}=N_{E}^{k}\hat{\mathrm{Var}}\left( \bar{x}_{E}^{k} \right)+N_{C}^{k}\hat{\mathrm{Var}}\left( \bar{x}_{C}^{k} \right),$$

where the component terms are provided as summary data from *k*th study, and $N_{E}^{k}$ and $N_{C}^{k}$ are the corresponding sample sizes in treatment and control groups. We estimated $\mathrm{Var}(d_{k})$ conditional on $S^{2}$ as

$$\hat{\mathrm{Var}}\left( d \right)=\hat{\mathrm{Var}}\left( \bar{x}_{E}^{k} \right)+\hat{\mathrm{Var}}\left( \bar{x}_{C}^{k} \right)$$

and used $\left[ \hat{Var}\left( d_{k} \right) \right]^{-1}d_{k}^{2}\sim\chi^{2}(1)$ under $H_{0}^{k}:E\left( d_{k} \right)=0$ vs $H_{1}^{k}:E\left( d_{k} \right)\neq0$ for asymptotic inference.

## **Text E. Calculation of number needed to treat/ harm (NNT)/(NNH)**

Continuous outcome (CDR-SB, ADAS-Cog): NNT

Calculation of NNT/NNH for scaled outcome measures is more challenging compared to that for categorical measures. In this study, we used a method first described by Kraemer HC et.al. in 2006 (Kraemer HC et.al., Biol Psychiatry 2006). There are reports arguing that Furukawa’s method is better than Kraemer’s method used in the paper (Furukawa TA et.al., Plos One 2011). However, to perform Furukawa’s method, we need to know the control event rate, which was not available in most of the trials. Also, it would be necessary to determine an appropriate cutoff for the reduction percentage (e.g., 50% reduction). However, selecting such a cutoff can be subjective and introduce potential bias. Therefore, we used the Kraemer’s method. Please note that Kraemer’s NNTs tend to provide smaller than the actual NNTs, so the interpretation of the result must be taken with careful consideration.

1. Normalize the ADAS-Cog scores

Given that different versions of the ADAS-Cog (versions 11, 13, 14) have varying maximum scores, the ADAS-Cog scores were normalized by dividing each score by the corresponding version's maximum possible score (70 for ver11, 85 for ver13, and 90 for ver14).

1. A Mann-Whitney U test was conducted to compare the pre- and post-treatment mean change in CDR-SB or normalized ADAS-Cog scores between the experimental and control groups, and the U value was calculated.

$$U_{e}=R_{e}-\frac{n_{e}(n_{e}+1)}{2}$$

$$U_{c}=R_{c}-\frac{n_{c}(n_{c}+1)}{2}$$

Where:

- R_e_ and R_c_ are the sum of ranks in the experimental and the control group
- n_e_ and n_c_ are the number of observations (e.g., trials) in the experimental and the control group).
- U_e_ and U_c_ are the Mann-Whitney’s U values in the experimental and the control group

$$U=min(U_{e},U_{c})$$

- The final U value is the smaller of the two U values.

1. Calculation of the area under curve (AUC) from Mann Whitney’s U value

$$AUC=\frac{U}{n_{e}\times n_{c}}$$

Where:

- AUC is the area under the curve of the receiver operating characteristic curve (ROC) comparing the experimental and the control groups scaled responses.
- For the rationale of calculating the AUC from U, please also refer to the following reference (Kraemer HC et.al, Size of Treatment Effects and Their importance to Clinical Research Practice. Biol Psychiatry 2006).

1. Calculation of the risk difference from the AUC

$$RD=2\times AUC-1$$

Where:

- RD is the risk difference between the experimental and the control group.

1. Calculation of the standard error of the AUC

$$SE\left( AUC \right)=\sqrt{\frac{AUC\left( 1-AUC \right)+\left( N_{1}-1 \right)\left( Q_{1}-{AUC}^{2} \right)+(N_{2}-1)(Q_{2}-{AUC}^{2})}{N_{1}\times N_{2}}}$$

Where:

$$Q_{1}=\frac{AUC}{2-AUC}$$

$$Q_{2}=\frac{2{AUC}^{2}}{1+AUC}$$

- SE(AUC) is the standard error of the AUC
- For the rationale of calculating the SE(AUC) from the above equations, please also refer to the following reference (Hanley JA et.al., The Meaning and Use of the Area under a Receiver Operating Characteristic (ROC) Curve, Radiology 1982).

1. Calculate the standard error of the risk difference from the standard error of the AUC

$$\mathrm{SE}\left( \mathrm{RD} \right)=SE(AUC)\times2$$

Where:

- SE(RD) is the standard error of the risk difference (RD)

1. Perform meta-analysis using the RD and the SE(RD) (similar to the categorical outcome), and calculate the NNT.

$$NNT=\frac{1}{RD.random}$$

Where:

- RD.random is a pooled risk difference in random-effect model

Categorical outcome (Secondary outcomes): NNH

1. Calculate the risk difference (RD)

$$RD=\frac{E_{e}}{N_{e}}-\frac{E_{c}}{N_{c}}=p_{e}-p_{c}$$

Where:

- RD is the risk difference between the experimental and the control groups.
- E_e_ and E_c_ are the number of events in the experimental and the control groups.
- N_e_ and N_c_ are the sample sizes of the experimental and the control groups.
- p_e_ and p_c_ are the proportion of events in the experimental and the control groups.

$$v=\frac{p_{e}(1-p_{c})}{n_{e}}+\frac{p_{c}(1-p_{c})}{n_{c}}$$

Where:

- v is the within-study variance.

1. Random-effects weights

$$w=\frac{1}{v+t^{2}}$$

Where:

- w is the weights in a random-effects model.
- t^2^ is the between-study variance (heterogeneity), estimated using the Paule-Mandel method.

1. Pooled estimate for risk difference

$$RD.random=\frac{\sum w_{i}\cdot RD_{i}}{\sum w_{i}}$$

$$NNH=\frac{1}{RD.random}$$

Where:

- NNH is a number needed to harm
- NNH was rounded up to the nearest integer.

NNH was calculated only when the results of main analysis results for the outcome is significant, due to a major discontinuity in NNH when the RD nears zero (Kraemer H.C et.al., 2006 Biol Psychiatry). Given this instability, calculating confidence intervals for NNH is not advisable. If individual patient-level data had been available, using Furukawa’s method (Furukawa TA et.al., Plos One 2011) to estimate NNT/NNH could have provided values that are more sensitive to the actual distribution of responders in the experimental and control groups. This might have resulted in NNT/NNH estimates that more accurately reflect the clinical reality of the dataset.

## **Text F. Comparison between meta and metafor packages**

In this study, the meta package (version 7.0-0) was used for all analyses. However, the metafor package is also widely utilized (Viechtbauer W et.al., 2010). To evaluate differences in outputs between the meta and metafor packages, we conducted additional analyses using the metafor package (version 4.6-0) and compared the results.

We present the outputs of four case studies:

(1) meta-analysis for continuous outcomes (outcome: CDR-SB, no modifier)

(2) meta-regression for continuous outcomes (outcome: CDR-SB, modifier: Drug)

(3) meta-analysis for categorical outcomes (outcome: ARIA-E, no modifier)

(4) meta-regression for categorical outcomes. (outcome: ARIA-E, modifier: Drug)

|  |  | **Mean difference** | **95％CI (Lower limit)** | **95％CI (Upper limit)** | **P Value** | **τ^2^** | **I^2^ (%)** |
| --- | --- | --- | --- | --- | --- | --- | --- |
| **Case study 1: outcome: change in CDR-SB, no modifier** | | | |  |  |  |  |
| Meta |  | -0.25 | -0.38 | -0.11 | <0.001 | 0.03 | 53.10 |
| Metafor |  | -0.25 | -0.38 | -0.11 | <0.001 | 0.03 | 53.56 |
| **Case study 2: outcome: change in CDR-SB, modifier: antibody** | | | |  |  |  |  |
| Meta | Lecanemab | -0.45 | -0.73 | -0.17 | 0.002 | 0.01 | 19.99 |
|  | Gantenerumab | 0.20 | -0.20 | 0.60 | 0.33 |  |  |
|  | Donanemab | -0.25 | -0.68 | 0.18 | 0.26 |  |  |
|  | Aducanumab | 0.25 | -0.11 | 0.61 | 0.17 |  |  |
|  | Solanezumab | 0.21 | -0.15 | 0.57 | 0.25 |  |  |
|  | Bapineuzumab | 0.39 | 0.04 | 0.75 | 0.03 |  |  |
| Metafor | Lecanemab | -0.45 | -0.73 | -0.17 | 0.00 | 0.01 | 19.99 |
|  | Gantenerumab | 0.20 | -0.20 | 0.60 | 0.33 |  |  |
|  | Donanemab | -0.25 | -0.68 | 0.18 | 0.26 |  |  |
|  | Aducanumab | 0.25 | -0.11 | 0.61 | 0.17 |  |  |
|  | Solanezumab | 0.21 | -0.15 | 0.57 | 0.25 |  |  |
|  | Bapineuzumab | 0.39 | 0.04 | 0.75 | 0.03 |  |  |
|  |  | **Risk Ratio** | **95％CI (Lower limit)** | **95％CI (Upper limit)** | **P Value** | **τ^2^** | **I^2^ (%)** |
| **Case study 3: outcome: ARIA-E, no modifier** | | |  |  |  |  |  |
| Meta |  | 9.79 | 5.32 | 18.01 | <0.001 | 0.87 | 59.90 |
| Metafor |  | 9.79 | 5.32 | 18.01 | 0.004 | 0.87 | 87.34 |
| **Case study 4: outcome: ARIA-E, modifier: antibody** | | | |  |  |  |  |
| Meta | Lecanemab | 2.02 | 0.60 | 3.44 | 0.01 | 0.45 | 70.70 |
|  | Gantenerumab | 0.24 | -1.51 | 1.99 | 0.79 |  |  |
|  | Donanemab | 0.44 | -1.55 | 2.43 | 0.67 |  |  |
|  | Aducanumab | 0.42 | -1.32 | 2.15 | 0.64 |  |  |
|  | Solanezumab | -1.65 | -3.70 | 0.41 | 0.12 |  |  |
|  | Bapineuzumab | 0.94 | -0.77 | 2.66 | 0.28 |  |  |
| Metafor | Lecanemab | 2.02 | 0.60 | 3.44 | 0.01 | 0.45 | 70.70 |
|  | Gantenerumab | 0.24 | -1.51 | 1.99 | 0.79 |  |  |
|  | Donanemab | 0.44 | -1.55 | 2.43 | 0.67 |  |  |
|  | Aducanumab | 0.42 | -1.32 | 2.15 | 0.64 |  |  |
|  | Solanezumab | -1.65 | -3.70 | 0.41 | 0.12 |  |  |
|  | Bapineuzumab | 0.94 | -0.77 | 2.66 | 0.28 |  |  |

CI: confidence interval, CDR-SB: Clinical Dementia Rating-Sum of Boxes, ARIA-E: amyloid-related imaging abnormalities with edema or effusion.

Overall, the main outcomes and 95% confidence intervals (mean differences for continuous outcomes and risk ratios for categorical outcomes), as well as the I^2^ and τ^2^ values, were identical or similar between the meta and metafor packages, except for the I^2^ values in case study 1 and 3.

Therefore, we additionally performed meta-analyses in primary and secondary endpoints using metafor package and summarized the I^2^ values below.

|  | **Meta** | **Metafor** |
| --- | --- | --- |
| **Primary endpoints** |  |  |
| CDR-SB | 53.10 | 53.56 |
| ADAS-Cog | 7.00 | 7.79 |
| **Secondary endpoints** |  |  |
| Death | 21.20 | 22.43 |
| SAE | 46.30 | 49.02 |
| ARIA-E | 59.90 | 87.34 |
| ARIA-H | 78.80 | 84.92 |
| Headache | 0 | 0 |
| Fall | 0 | 0 |
| Dizziness | 0 | 0 |

I^2^ values (%). CDR-SB: Clinical Dementia Rating-Sum of Boxes, ADAS-Cog: ARIA-E: amyloid-related imaging abnormalities with edema or effusion, ARIA-H: amyloid-related imaging abnormalities with hemorrhage.

The difference in output is derived from the difference in calculation methods. In Meta package, I^2^ is calculated based on the following equation (Schwarzar G et.al., Springer 2015. [doi:10.1007/978-3-319-21416-0](https://doi.org/10.1007%2F978-3-319-21416-0)).

$$I^{2}=\frac{Q-df}{Q} \times100$$

where Q represents Cochran’s Q statistic, and df denotes the degree of freedom.

On the other hand, I^2^ is calculated based on the following equation in Metafor package (The metafor Package: I^2^ for Multilevel and Multivariate Models. [Available from: <https://www.metafor-project.org/doku.php/tips%3Ai2_multilevel_multivariate>]).

$$I^{2} =\frac{\hat{\tau}^{2}}{\hat{\tau}^{2}+\tilde{\upsilon}}$$

Where $\hat{\tau}^{2}$ is the estimated value of τ^2^ and

$$\tilde{\upsilon}= \frac{df\sum\omega_{i}}{{(\sum\omega_{i})}^{2}-\sum\omega_{i}^{2}}$$

Where

$$\omega_{i}=\frac{1}{\upsilon_{i}}$$

is the inverse of the sampling variance of the i_th_ study.

Both approaches are described in the original publication where I^2^ was introduced by Higgins et.al., in 2002 for the first time (Higgins JPT et.al., Statist Med 2002). Using the criteria Higgins JPT et.al., proposed in the same paper, we judged the heterogeneity mild if I^2^ is less than 30%, moderate if I^2^ is 30-50%, and substantial if I^2^ is >50%. Using this criteria, there was no discrepancy in interpretation of heterogeneity based on the output from Meta and Metafor packages.

The R script used for this comparative analysis is available in github (<https://github.com/ChengF-Lab/Meta_ADAb>, R script name: s_meta_ab_antibody_Text.R).

## **Text G. Definition of serious adverse events (SAE)**

Following is the summary of the definition of SAE. For further information, please look at the protocols of each trial.

***GRADUATE I/ GRADUATE II***

A serious adverse event is any adverse event that meets any of the following criteria:

- Is fatal (i.e., the adverse event actually causes or leads to death)
- Is life threatening (i.e., the adverse event, in the view of the investigator, places the patient at immediate risk of death)

This does not include any adverse event that had it occurred in a more severe form or was allowed to continue might have caused death.

- Requires or prolongs inpatient hospitalization
- Results in persistent or significant disability/incapacity (i.e., the adverse event results in substantial disruption of the patient’s ability to conduct normal life functions)
- Is a significant medical event in the investigator's judgment (e.g., may jeopardize the patient or may require medical/surgical intervention to prevent one of the outcomes listed above)

***TRAILBLAZER-ALZ2***

Results in death, is life-threatening, required inpatient hospitalization or prolongation of existing hospitalization, results in persistent disability/incapacity, or based on other medical/scientific judgement.

***Clarity_AD***

A SAE is any untoward medical occurrence that at any dose:

- Results in death
- Is life-threatening (ie, the subject was at immediate risk of death from the adverse event (AE) as it

occurred; this does not include an event that, had it occurred in a more severe form or

was allowed to continue, might have caused death)

- Requires inpatient hospitalization or prolongation of existing hospitalization
- Results in persistent or significant disability/incapacity
- Is a congenital anomaly/birth defect (in the child of a subject who was exposed to the study drug)

Other important medical events that may not be immediately life-threatening or result in death or hospitalization but, when based on appropriate medical judgment, may jeopardize the subject or may require intervention to prevent one of the outcomes in the definition of SAE listed above should also be considered SAEs. Medical and scientific judgment should be exercised in deciding whether expedited reporting is appropriate in such situations. In addition to the above, events associated with special situations include pregnancy or exposure to study drug through breastfeeding; AEs associated with study drug overdose, misuse, abuse, or medication error. These events associated with special situations are to be captured using the SAE procedures but are to be considered as SAEs only if they meet 1 of the above criteria. All AEs associated with special situations are to be reported on the clinical research form whether or not they meet the criteria for SAEs.

The following hospitalizations are not considered to be SAEs because there is no “adverse event” (ie, there is no untoward medical occurrence) associated with the hospitalization:

- Hospitalizations for respite care
- Planned hospitalizations required by the protocol
- Hospitalization planned before informed consent (where the condition requiring the hospitalization has not changed post study drug administration)
- Hospitalization for administration of study drug or insertion of access for administration of study drug
- Hospitalization for routine maintenance of a device (eg, battery replacement) that was in place before study entry

***EMERGE/ENGAGE***

Results in death, in the view of the investigator, places the subject at immediate risk of death (a life-threatening event): However, this does not include an event that, had it occurred in a more severe form, might have caused death. Requires inpatient hospitalization or prolongation of existing hospitalization. Results in persistent or significant disability/incapacity. Results in a congenital anomaly/birth defect. An SAE may also be any other medically important event that, in the opinion of the Investigator, may jeopardize the subject or may require intervention to prevent one of the other outcomes listed in the definition above. (Examples of such medical events include allergic bronchospasm requiring intensive treatment in an emergency room or convulsions occurring at home that do not require an inpatient hospitalization.)

***EXPEDITION1/EXPEDITION2***

SAE collection begins after the patient has signed informed consent and has received study drug. If a patient experiences a SAE after signing informed consent, but before receiving study drug, the event will NOT be collected unless the investigator

feels the event may have been caused by a protocol procedure. Study site personnel must alert Lilly or its designee of any SAE within 24 hours of investigator awareness of the event via a sponsor-approved method. Alerts issued via telephone are to be immediately followed with official notification on study-specific SAE forms. A SAE is any AE from this study that results in one of the

following outcomes:

- Death
- Initial or prolonged inpatient hospitalization
- A life-threatening experience (that is, immediate risk of dying)
- Persistent or significant disability/incapacity
- Congenital anomaly/birth defect
- Considered significant by the investigator for any other reason

Important medical events that may not result in death, be life-threatening, or require hospitalization may be considered serious adverse drug events when, based upon appropriate medical judgment, they may jeopardize the patient or patient and may require medical or surgical intervention to prevent one of the outcomes listed in this definition. Serious adverse events occurring after a patient has been administered the last dose of

study drug will be collected for 30 days after the last dose of study drug, regardless of the investigator’s opinion of causation. Thereafter, SAEs are not required to be reported unless the investigator feels the events were related to either study drug, drug delivery system, or a protocol procedure.

***EXPEDITION 3***

An SAE is any AE from this study that results in one of the following outcomes:

- death
- initial or prolonged inpatient hospitalization
- a life-threatening experience (that is, immediate risk of dying)
- persistent or significant disability/incapacity
- congenital anomaly/birth defect
- considered significant by the investigator for any other reason.

Important medical events that may not result in death, be life-threatening, or require hospitalization may be considered serious adverse drug events when, based upon appropriate medical judgment, they may jeopardize the patient and may require medical or surgical intervention to prevent one of the outcomes listed in this definition.

***Study 300/301***

An SAE is one that meets one or more of the following:

- Is fatal;
- Is immediately life-threatening;
- Is permanently (or significantly) disabling;
- Requires hospitalization;
- Prolongs existing hospitalization;
- Is a congenital anomaly or birth defect (in an offspring);
- Is medically significant.

***Study 3001/3002***

SAEs were not clearly defined nor reported.

## **Text H. Definition of cerebral macrohemorrhage**

***GRADUATE I, II***

Intraparenchymal and subarachnoid hemorrhage.

***TRAILBLAZER-ALZ2***

Intracerebral hemorrhage >1cm.

***Clarity_AD***

“Macrohemorrhage”, that was >10mm at greatest diameter regardless of symptomatic or not.

***Study 3000/3001***

Intraparenchymal hemorrhage.

***Study 300/301***

“Cerebral hemorrhage” in treatment-emergent serious events.

# **Supplemental Tables**

## **Table A. Trials that were included in the analyses**

|  | CDR-SB | ADAS-Cog | Death | SAE | ARIA-E | ARIA-H | Headache | Fall | Dizziness | Cerebral hemorrhage |
| --- | --- | --- | --- | --- | --- | --- | --- | --- | --- | --- |
| GRADUATE I | y | y | y | y | y | y | y | y | y | y^*2^ |
| GRADUATE II | y | y | y | y | y | y | y | y | y | y^*2^ |
| TRAIBLAZER-ALZ2 | y | y | y | y | y | y | y | y | y | y^*3^ |
| Clarity_AD | y | y | y | y | y | y | y | y | y | y^*4^ |
| EMERGE | y | y | y | y | y | y | y | y | y | n |
| ENGAGE | y | y | y | y | y | y | y | y | y | n |
| EXPEDITION3 | y | y | y | y | y | n | y | y | y | n |
| EXPEDITION1 | y | y | y^*1^ | y^*1^ | y^*1^ | y^*1^ | y^*1^ | y^*1^ | y^*1^ | n |
| EXPEDITION2 | y | y | y^*1^ | y^*1^ | y^*1^ | y^*1^ | y^*1^ | y^*1^ | y^*1^ | n |
| Study3000 | y | y | y | n | y | n | n | n | n | y^*5^ |
| Study3001 | y | y | y | n | y | n | n | n | n | y^*5^ |
| Study300 | y | y | y | y | y | n | y | y | n | y^*6^ |
| Study301 | y | y | y | y | y | n | y | y | y | y^*6^ |

y: included in the analysis. n: data was not available, thus not included in the analysis. *1: pooled data for EXPEDITION 1 and 2 were included. *2: “Intraparenchymal and subarachnoid hemorrhage”. *3: Intracerebral hemorrhage >1cm. *4: Macrohemorrhage. *5: Intraparenchymal hemorrhage. *6: “Cerebral hemorrhage” in treatment-emergent serious events.

CDR-SB: Clinical Dementia Rating-Sum of Boxes, ADAS-Cog: Alzheimer's Disease Assessment Scale - Cognitive Subscale, SAE: Serious adverse event, ARIA-E: Amyloid-Related Imaging Abnormalities-Effusion, ARIA-H: Amyloid-Related Imaging Abnormalities-Hemorrhage.

## **Table B. Results of meta-regression analyses for death, fall, and dizziness.**

|  | **Death** |  | **Fall** |  | **Dizziness** |  |
| --- | --- | --- | --- | --- | --- | --- |
|  | **Risk Ratio (95%CI)** | **P value** | **Risk Ratio (95%CI)** | **P value** | **Risk Ratio (95%CI)** | **P value** |
| **Mean age** | 0.99 (0.77 to 1.26) | 0.91 | 0.96 (0.90 to 1.02) | 0.15 | 1.01 (0.93 to 1.10) | 0.84 |
| **Mean MMSE** | 0.89 (0.73 to 1.07) | 0.22 | 1.04 (1.00 to 1.08) | 0.08 | 0.99 (0.93 to 1.05) | 0.65 |
| **APOE4 carrier percentage** | 1.00 (0.99 to 1.02) | 0.72 | 1.00 (1.00 to 1.01) | 0.38 | 1.00 (0.99 to 1.02) | 0.67 |
| **Ab burden on PET** | 1.01 (0.93 to 1.09) | 0.84 | 1.00 (0.98 to 1.01) | 0.61 | 1.00 (0.98 to 1.02) | 0.72 |
| **AD-Stage** | **RR compared to placebo (95% CI)** | **P values for comparison with the reference** | **RR compared to placebo (95% CI)** | **P values for comparison with the reference** | **RR compared to placebo (95% CI)** | **P values for comparison with the reference** |
| Early (reference) | 0.86 (0.54 to 1.38) | NA | 1.06 (0.96 to 1.18) | NA | 1.07 (0.93 to 1.23) | NA |
| Mild-moderate | 1.18 (0.70 to 1.97) | 0.38 | 0.93 (0.79 to 1.10) | 0.18 | 1.07 (0.82 to 1.41) | 0.99 |
| **Drug** | **RR compared to placebo (95% CI)** | **P values for comparison with the reference** | **RR compared to placebo (95% CI)** | **P values for comparison with the reference** | **RR compared to placebo (95% CI)** | **P values for comparison with the reference** |
| Lecanemab (reference) | 0.86 (0.18 to 4.12) | NA | 1.08 (0.81 to 1.44) | NA | 1.06 (0.72 to 1.57) | NA |
| Gantenerumab | 0.71 (0.21 to 2.33) | 0.85 | 0.94 (0.74 to 1.21) | 0.48 | 1.14 (0.84 to 1.55) | 0.78 |
| Donanemab | 1.63 (0.41 to 6.51) | 0.54 | 1.06 (0.82 to 1.37) | 0.93 | 1.13 (0.77 to 1.65) | 0.83 |
| Aducanumab | 0.97 (0.23 to 4.12) | 0.91 | 1.19 (0.97 to 1.45) | 0.59 | 1.01 (0.80 to 1.27) | 0.81 |
| Solanezumab | 0.85 (0.33 to 2.18) | 0.99 | 0.97 (0.79 to 1.19) | 0.47 | 1.05 (0.82 to 1.34) | 0.95 |
| Bapineuzumab | 1.06 (0.46 to 2.45) | 0.81 | 0.98 (0.80 to 1.21) | 0.60 | 1.16 (0.76 to 1.77) | 0.77 |
| **Antibody type** | **RR compared to placebo (95% CI)** | **P values for comparison with the reference** | **RR compared to placebo (95% CI)** | **P values for comparison with the reference** | **RR compared to placebo (95% CI)** | **P values for comparison with the reference** |
| human (reference) | 0.77 (0.36 to 1.66) | NA | 1.09 (0.93 to 1.27) | NA | 1.05 (0.88 to 1.27) | NA |
| humanized | 1.06 (0.71 to 1.58) | 0.47 | 1.00 (0.90 to 1.11) | 0.36 | 1.08 (0.92 to 1.28) | 0.82 |
| **Binding mechanism** | **RR compared to placebo (95% CI)** | **P values for comparison with the reference** | **RR compared to placebo (95% CI)** | **P values for comparison with the reference** | **RR compared to placebo (95% CI)** | **P values for comparison with the reference** |
| Monomer (reference) | 0.88 (0.45 to 1.73) | NA | 0.96 (0.81 to 1.12) | NA | 1.05 (0.82 to 1.34) | NA |
| Oligomer or Aggregates | 0.94 (0.58 to 1.51) | 0.88 | 1.05 (0.94 to 1.18) | 0.33 | 1.07 (0.92 to 1.25) | 0.90 |
| Plaque only | 1.64 (0.58 to 4.59) | 0.32 | 1.06 (0.83 to 1.36) | 0.49 | 1.13 (0.77 to 1.65) | 0.74 |
| **Biological effect** | **RR compared to placebo (95% CI)** | **P values for comparison with the reference** | **RR compared to placebo (95% CI)** | **P values for comparison with the reference** | **RR compared to placebo (95% CI)** | **P values for comparison with the reference** |
| Yes (reference) | 1.04 (0.67 to 1.59) | NA | 1.06 (0.95 to 1.17) | NA | 1.08 (0.93 to 1.24) | NA |
| No | 0.88 (0.45 to 1.73) | 0.69 | 0.96 (0.82 to 1.12) | 0.31 | 1.05 (0.82 to 1.34) | 0.86 |

For the categorical modifiers (AD-stage, drug, antibody type, binding mechanism and biological effect), risk ratio compared to placebo and p-values for comparison with the reference group are are shown. Reference groups were shown in the table. MMSE: mini-mental state examination, AD; Alzheimer’s Disease, RR: risk ratio.

## **Table C. Summary of amyloid PET**

| **Trial name** | **Antibody** | **Study population** | **Tracer** | **Unit** | **Timing** | **Outcome** | **Analytical methods** | **Results** | **Interpretation** |
| --- | --- | --- | --- | --- | --- | --- | --- | --- | --- |
| GRADUATE I [13] | Gantenerumab | Antibody:65  Control:58 | ^18^F-florbetapen or ^18^F-flutemetamol | centiloids | 0, 52, 104, 116 weeks | Change from baseline to week 116 in centiloid. | MMRM  Adjusting for treatment arm, type of tracer, baseline centiloid, baseline centiloid-by-visit and treatment-by-visit interaction. | Mean difference:  -66.44 (95%CI:  -74.71,  -58.16) centiloids | Decrease |
| GRADUATE II [13] | Gantenerumab | Antibody:58  Control:56 | ^18^F-florbetapen or ^18^F-flutemetamol | centiloids | 0, 52, 104, 116 weeks | Change from baseline to week 116 in centiloid. | MMRM  Adjusting for treatment arm, type of tracer, baseline centiloid, baseline centiloid-by-visit and treatment-by-visit interaction. | Mean difference:  -56.46 (95%CI:  -64.36, -48.00) centiloids | Decrease |
| TRAILBLAZER-ALZ2 [4] | Donanemab | Antibody: 765  Control: 812 | ^18^F-florbetapir | centiloids | 0, 24, 52, 76 weeks | Change from baseline to the post-baseline visit of the amyloid imaging in centiloid. | MMRM model with fixed effects of treatment, visit, and treatment-by-visit interaction, and age at baseline. | At 76 weeks.  Mean change: Antibody: 87.0 (95%CI:  -88.90,  -85.17)  Control: 0.67 (95%CI:  -2.45, 1.11) centiloids | Decrease |
| Clarity_AD [2] | Lecanemab | Antibody:354  Placebo:344 | ^18^F-florbetapir,  ^18^F-flutemetamol,  or ^18^F-flutemetamol | centiloids | 0,3,6,12,18 months | Change from baseline at 18 months in amyloid burden on PET as measured in centiloids | MMRM including baseline value as a covariate, with treatment group, visit, use of AD symptomatic medication at baseline, APOE4 carrier status, geographical region, baseline value-by-visit, treatment group-by-visit interaction as fixed effects. | Mean difference:  -59.12 (95%CI: -62.64, -55.60; P<0.001) centiloids | Decrease |
| EMERGE [3] | Aducanumab | Low-dose: 293  High-dose: 293  Control: 287 | ^18^F-florbetapir,  ^18^F-flutemetamol,  or ^18^F-florbetaben | SUVR | 0,56,78 weeks | Adjusted mean change from baseline between high-dose Aducanumab and Placebo | MMRM adjusted for fixed effects of treatment, visit, treatment by visit interaction, baseline SUVR, baseline SUVR by visit interaction, baseline MMSE, baseline APOE4 status, and baseline age. | Mean difference:  High-dose:  -0.28 (95%CI, -0.31, -0.25; P<0.001)  For the low-dose group, only a line graph was provided. | Low-dose: Decrease,  High-dose: Decrease |
| ENGAGE [3] | Aducanumab | Low-dose: 331  High-dose: 281  Control: 333 | ^18^F-florbetapir,  ^18^F-flutemetamol,  or ^18^F-florbetaben | SUVR | 0,56,78 weeks | Adjusted mean change from baseline between high-dose Aducanumab and Placebo | MMRM adjusted for fixed effects of treatment, visit, treatment by visit interaction, baseline SUVR, baseline SUVR by visit interaction, baseline MMSE, baseline APOE4 status, and baseline age. | Mean difference:  High-dose:  -0.23 (95%CI, -0.26, -0.21; P<0.001)  For the low-dose group, only a line graph was provided. | Low-dose: Decrease,  High-dose: Decrease |
| EXPEDITION3 [16] | Solanezumab | Not mentioned clearly. | ^18^F-florbetapir | SUVR | 0, 80 weeks | Change in SUVR, reference to the cerebellum and normalized subject-specific white matter region, from baseline to post-baseline assessment | ANCOVA model containing terms for baseline SUVR, treatment, and age at baseline. | Mean SUVR change:  Antibody:  0.02 $\pm$ 0.002  Placebo: 0.02 $\pm$ 0.002 p=0.13 | N.S |
| EXPEDITION1 [17] | Solanezumab | 169 patients | ^18^F-florbetapir | SUVR | 0,80 weeks, or at an early termination | The annualized change in the composite summary SUVR of^18^F-florbetapir. | ANCOVA model including baseline florbetapir F-18 level, pooled investigator, treatment, MMSE stratification factor at Visit1 (mild or moderate), and age at baseline. | Not reported in detail. | N.S |
| EXPEDITION2 [17] | Solanezumab | 97 patients | ^18^F-florbetapir | SUVR | 0,80 weeks, or at an early termination | The annualized change in the composite summary SUVR of ^18^F-florbetapir | ANCOVA model including baseline florbetapir F-18 level, pooled investigator, treatment, MMSE stratification factor at Visit1 (mild or moderate), and age at baseline. | Not reported in detail. | N.S |
| Study 3000 [32] | Bapineuzumab | Antibody: 17  Placebo: 13 | Pittsburgh Compound B  (PIB-PET) | SUVR | 0,45,71 weeks | Change from baseline to week71. | Not clearly described. | P=0.65  Line graphs are provided. | N.S |
| Study 3001 [32] | Bapineuzumab | Antibody: 32  Placebo: 24 | Pittsburgh Compound B  (PIB-PET) | SUVR | 0,45,71 weeks | Change from baseline to week71. | Not clearly described. | P=0.16  Line graphs are provided. | N.S |
| Study 301 [33] | Bapineuzumab | Antibody  Low-dose: 12  High-dose: 12  Placebo: 15 | Pittsburgh Compound B  (PIB-PET) | SUVR | 0,45,71 weeks | Change in SUVR average from baseline to week 71. | MMRM including the baseline ADAS-Cog 11 or DAD value, MMSE score stratum, baseline cholinesterase inhibitor or memantine use stratum, APOE E4 copy number stratum, and schedule visit. | Low-dose: P=0.19,  High-dose: P=0.47  Line graphs are provided. | Low-dose: N.S,  High-dose: N.S |
| Study 302 [33] | Bapineuzumab | Antibody:75  Placebo: 40 | Pittsburgh Compound B  (PIB-PET) | SUVR | 0,45,71 weeks | Change in SUVR average from baseline to week 71. | MMRM including the baseline ADAS-Cog 11 or DAD value, MMSE score stratum, baseline cholinesterase inhibitor or memantine use stratum, APOE E4 copy number stratum, and schedule visit. | Difference:  -0.10  SUVR, P=0.004, Line graphs are provided. | Decrease |

In the result section, “decrease” indicates that the level of Ab or tau uptake is significantly decrease in the treatment (antibody) group compared to the placebo group.

PET: positron emission tomography, SUVR: standardized uptake value ratios, MMRM: Mixed-effects model for repeated measures, ANCOVA: Analysis of covariance, NA: not applicable, N.S: non-significant.

## **Table D. Summary of tau PET results**

| **Trial name** | **Antibody** | **Study population** | **Tracer** | **Unit** | **Timing** | **Outcome** | **Analytical methods** | **Results** | **Interpretation** |
| --- | --- | --- | --- | --- | --- | --- | --- | --- | --- |
| GRADUATE I [13] | Gantenerumab | Antibody: 109  Placebo: 92 | ^18^F-GTP | SUVR | 0, 52, and 116 weeks | Change from baseline in cortical SUVR | MMRM adjusting for treatment arm, visit, APOE4 status (as categorical: carrier vs non-carrier), baseline median SUVR, baseline SUVR-by-visit, study, study-by-visit and treatment-by-visit interaction. | Mean difference  Medial:0.01 (95%CI, -0.03, 0.05)  Temporal:0.01(95%CI, -0.04, 0.05)  Frontal: 0.00 *1  Parietal: 0.00 *1 | N.S (Medical, Temporal, and Frontal composite) |
| GRADUATE II [13] | Gantenerumab | Pool*2 | ^18^F-GTP | SUVR | Pool*2 | Pool*2 | Pool*2 | Pool*2 | Pool*2 |
| TRAILBLAZER-ALZ2 [4] | Donanemab | Antibody:578  Placebo:654 | ^18^F-flortaucipir | SUVR | 0, 76 weeks | Difference in Change from baseline in tau imaging parameters (including global and regional tau SUVR). | Analysis of covariance (ANCOVA) model, adjusted by baseline tau SUVR, and age at baseline. | Difference in change in the combined population:  -0.004 (95%CI: -0.01, 0.01; P=0.45). | N.S |
| Clarity_AD [2] | Lecanemab | NR  (it will be reported in a separate paper) | NR | NR | NR | NR | MMRM (it will be reported in a separate paper) | NR | NR |
| EMERGE [3] | Aducanumab | Low-dose: 14  High-dose: 11  Placebo: 12 | ^18^F-MK-6240 | SUVR  Six composite regions of interests normalized to cerebellar cortex. | 0,78 weeks | Change from baseline in each composite regions (frontal, temporal, medial temporal, parietal, cingulate, occipital). | ANCOVA model including treatment group, baseline SUVR value, APOE4 status as covariates. | Since only bar graphs were provided, detailed numerical values were not extracted in this study. | Low-dose: Decrease in Medial temporal, N.S in other areas.  High-dose: Decrease in Medial temporal, temporal, and frontal regions, and N.S in other areas. |
| ENGAGE [3] | Aducanumab | Pool*3 | ^18^F-MK-6240 | SUVR | Pool*3 | Pool*3 | Pool*3 | Pool*3 | Pool*3 |
| EXPEDITION3 [16] | Solanezumab | Not mentioned clearly. | ^18^F-flortaucipir | SUVR | 0,40,80 weeks. | Change in SUVR normalized to bimodal white matter region from baseline in least-square mean at Week 80. | MMRM model adjusted for baseline SUVR, treatment, scan order, age at baseline, and treatment-and-visit-interaction. | Mean SUVR change:  Antibody: 0.05 $\pm$ 0.01, Placebo: 0.05 $\pm$ 0.01. p=0.69 | N.S |
| EXPEDITION1 [17] | Solanezumab | NR | NR | NR | NR | NR | NR | NR | NR |
| EXPEDITION2 [17] | Solanezumab | NR | NR | NR | NR | NR | NR | NR | NR |
| Study 3000 [32] | Bapineuzumab | NR | NR | NR | NR | NR | NR | NR | NR |
| Study 3001 [32] | Bapineuzumab | NR | NR | NR | NR | NR | NR | NR | NR |
| Study 301 [33] | Bapineuzumab | NR | NR | NR | NR | NR | NR | NR | NR |
| Study 302 [33] | Bapineuzumab | NR | NR | NR | NR | NR | NR | NR | NR |

*1: 95%CIs were not reported in mean difference in the frontal and parietal cortex. *2: pooled data for GRADUATE1 and GRADUATE 2 was reported. *3: pooled data of EMERGE and ENGAGE was reported.

In the result section, “decrease” indicates that the level of Ab or tau uptake is significantly decrease in the treatment (antibody) group compared to the placebo group.

PET: positron emission tomography, SUVR: standardized uptake value ratios, MMRM: mixed model for repeated measures, ANCOVA: Analysis of covariance, NA: not applicable, N.S: non-significant.

## **Table E. Summary of cerebrospinal fluid biomarker results**

| **Trial name** | **Antibody** | **Study population** | **Endpoint** | **Analytical method** | **Aβ40** | **Aβ42** | **p-tau181** |
| --- | --- | --- | --- | --- | --- | --- | --- |
| GRADUATE I [13] | Gantenerumab | **Aβ40:**  Antibody 161  Placebo 154  **Aβ42:**  Antibody 162  Placebo 153  **p-tau 181:**  Antibody 159  Placebo 155 | Mean change from baseline to week 116. | ANCOVA analysis adjusting for treatment arm, study, APOE4 status, baseline biomarker level. | ↓  Baseline Mean (pg/mL)  Antibody: 16213  Placebo: 15951  Difference  -10.4%, 95%CI (-18.3, -1.80) | ↑  Baseline Mean (pg/mL)  Antibody: 567  Placebo: 561  Difference:  37.4%, 95%CI (29.8, 48.3) | ↓  Baseline (pg/mL)  Antibody: 32  Placebo: 32  Difference: -23.8 (-28.1, -19.3) |
| GRADUATE II [13] | Gantenerumab | Pool*^1^ | Pool*^1^ | Pool*^1^ | Pool*^1^ | Pool*^1^ | Pool*^1^ |
| TRAILBLAZER-ALZ2 [4] | Donanemab | NR | NR | NR | NR | NR | NR |
| Clarity_AD [2] | Lecanemab | **Aβ40:**  Antibody 104  Placebo 108  **Aβ42:**  Antibody 134  Placebo 135  **p-tau 181:**  Antibody 134  Placebo 137 | Change from baseline at 18 months | MMRM including baseline value as a covariate, with treatment group, visit, use of AD symptomatic medication at baseline, APOE4 carrier status, geographical region, baseline value-by-visit, treatment group-by-visit interaction as fixed effects. | ↓  Since Only line graphs were provided, detailed numerical values were not extracted in this study. | ↑  Since Only line graphs were provided, detailed numerical values were not extracted in this study. | ↓  Since Only line graphs were provided, detailed numerical values were not extracted in this study. |
| EMERGE [3] | Aducanumab | Antibody  Low-dose: 33  High-dose: 17  Placebo: 28 | Change from baseline value at week 78. | ANCOVA model including treatment groups, baseline biomarker value, APOE4 status and baseline age. | NR | Low-dose: ↑,  High-dose: ↑  Since only bar graphs were provided, detailed numerical values were not extracted in this study. | Low-dose: ↓,  High-dose: ↓  Since only bar graphs were provided, detailed numerical values were not extracted in this study. |
| ENGAGE [3] | Aducanumab | Antibody  Low-dose: 20  High-dose: 18  Placebo: 15 | Change from baseline value at week 78. | ANCOVA model including treatment groups, baseline biomarker value, APOE4 status and baseline age. | NR | Low-dose: N.S,  High-dose: ↑  Since only bar graphs were provided, detailed numerical values were not extracted in this study. | Low-dose: N.S,  High-dose: N.S  Since only line graphs were provided, detailed numerical values were not extracted in this study. |
| EXPEDITION3 [16] | Solanezumab | Antibody:  Placebo: | Change from baseline at week 80. | ANCOVA model containing terms for baseline treatment, and age at baseline. | ↑  P<0.01  Bar graphs are shown. | ↑  P<0.01  Bar graphs are shown. | NR |
| EXPEDITION1 [17] | Solanezumab | Antibody: 20  Placebo: 25 | Change from baseline at week 80. | ANCOVA model with fixed effects of baseline CSF, pooled investigator, treatment, MMSE stratification factor at visit 1 (mild or moderate), and age at baseline. | ↑  Mean difference, 95%CI  3227.6 (1253.6, 5201.5) pg/mL | ↑  Mean difference  713.7, 95%CI (309.1,1118.4) pg/mL | NR |
| EXPEDITION2 [17] | Solanezumab | Antibody: 44  Placebo: 32 | Change from baseline at week 80. | ANCOVA model with fixed effects of baseline CSF, pooled investigator, treatment, MMSE stratification factor at visit 1 (mild or moderate), and age at baseline. | ↑  Mean difference  3033.1, 95%CI (1628.4, 4437.9) pg/mL | ↑  Mean Difference  402.8, 95%CI (307.7, 497.8) pg/mL | NR |
| Study 3000 [32] | Bapineuzumab | Antibody: 43  Placebo: 33 | Change from baseline to week 71. | Not clearly described. | NR | NR | N.S  P=0.09  Line graphs are provided. |
| Study 3001 [32] | Bapineuzumab | Antibody: 76  Placebo: 62 | Change from baseline to week 71. | Not clearly described. | NR | NR | N.S  P=0.62  Line graphs are provided. |
| Study 301 [33] | Bapineuzumab | Antibody:  Low-dose: 47  High-dose: 54  Placebo: 77 | Change from baseline at week 71. | ANCOVA model with terms of treatment, baseline, MMSE score stratum, baseline cholinesterase inhibitor or memantine use stratum, and APOE E4 copy number stratum. | NR | NR | Low-dose: N.S  0.05 pg/mL, P=0.98,  High-dose: ↓  Difference:  -6.19 pg/mL, P=0.01 |
| Study 302 [33] | Bapineuzumab | Antibody:127  Placebo:85 | Change from baseline at week 71. | ANCOVA model with terms of treatment, baseline, MMSE score stratum, baseline cholinesterase inhibitor or memantine use stratum, and APOE E4 copy number stratum. | NR | NR | ↓  Difference: -6.75 pg/mL, P=0.01 |

*1 For GRADUATE I and II, pooled data were published for cerebrospinal fluid. “↑” indicates that the level of the biomarker is significantly increased in the treatment (antibody) group compared to that of placebo group. “↓” indicates that the level of biomarker is significantly decrease in the treatment (antibody) group compared to the placebo group.

Aβ: Amyloid beta, MMRM: Mixed-effects model for repeated measures, ANCOVA: Analysis of covariance, NR: not reported, N.S: non-significant.

## **Table F. Summary of plasma biomarker results**

| **Trial name** | **Antibody** | **Study population** | **Endpoint** | **Analytical method** | **Aβ** | **Aβ 40** | **Aβ 42** | **Aβ 42/40 ratio** | **p-tau181** | **p-tau 217** |
| --- | --- | --- | --- | --- | --- | --- | --- | --- | --- | --- |
| GRADUATE I [13] | Gantenerumab | Antibody: 496  Placebo: 483 | Change from baseline to week 116. | MMRM adjusted for treatment arm, visit, treatment-by-visit interaction, APOE4 carrier status, baseline biomarker and biomarker biomarker-by-visit. | NR | NR | ↑  Baseline (pg/mL):  Antibody: 30.23  Placebo: 29.89  Difference at week 116  60.0, 95%CI (54.6, 65.7) % | NR | ↓  Baseline (pg/mL):  Antibody:1.58  Placebo: 1.55  Difference at week 116  -24.0, 95%CI (26.5, -21.4) % | NR |
| GRADUATE II [13] | Gantenerumab | Antibody: 497  Placebo: 475 | Change from baseline to week 116. | MMRM adjusted for treatment arm, visit, treatment-by-visit interaction, APOE4 carrier status, baseline biomarker and biomarker biomarker-by-visit. | NR | NR | ↑  Baseline (pg/mL):  Antibody: 31.01  Placebo: 31.72  Difference at week 116  51.8, 95%CI (46.7, 57.1) % | NR | ↓  Baseline (pg/mL):  Antibody:1.58  Placebo: 1.65  Difference at week 116  -21.0, 95%CI (-23.5, -18.4) % | NR |
| TRAILBLAZER-ALZ2 [4] | Donanemab | Antibody: 758  Placebo:  786 | Change from baseline at 76 weeks. | MMRM adjusted for fixed, categorical effects of treatment, visit, and treatment-by-visit interaction, and baseline value, baseline value-by-visit interaction and age at baseline. | NR | NR | NR | NR | NR | ↓  The difference in LSM change in tau SUVR (log10-based) vs placebo was -0.22, 95%CI (-0.24, -0.20), P<.001 in the combined population.  (The text is presented as is.) |
| Clarity_AD [2] | Lecanemab | Antibody: 746  Placebo: 752 | Change from baseline in 18 months. | MMRM including baseline value as a covariate, with treatment group, visit, use of AD symptomatic medication at baseline, APOE4 carrier status, geographical region, baseline value-by-visit, treatment group-by-visit interaction as fixed effects. | NR | NR | NR | ↑  Since Only line graphs were provided, detailed numerical values were not extracted in this study. | ↓  Since Only line graphs were provided, detailed numerical values were not extracted in this study. | NR |
| EMERGE [3] | Aducanumab | Antibody  Low-dose: 293  High-dose: 290  Placebo: 287 | Change from baseline at week 78. | Not specified precisely.  An MMRM model or an analysis of covariance may have been used. | NR | NR | NR | NR | Low-dose: ↓  (only line graph shown, more modest decrease compared to that in high-dose), High-dose: ↓  -0.67, 95%CI (-0.86, -0.47) pg/mL | NR |
| ENGAGE [3] | Aducanumab | Antibody  Low-dose: 331  High-dose: 281  Placebo: 333 | Change from baseline at week 78. | Not specified precisely.  An MMRM model or an analysis of covariance may have been used. | NR | NR | NR | NR | Low-dose: ↓ (only line graph shown, more modest decrease compared to that in high-dose),  High-dose: ↓  -0.78, 95%CI (-0.93, -0.62) pg/mL | NR |
| EXPEDITION3 [16] | Solanezumab | **Aβ40**:  Antibody: 1016  Placebo: 1050  **Aβ42**:  Antibody: 1030  Placebo: 1050 | Change from baseline to 4, 12,28,52, and 80 weeks. | MMRM to compare change from baseline to 4, 12, 28, 52, and 80 weeks after randomization. | NR | ↑  P<0.01  (Only P-value was reported.) | ↑  P<0.01  (Only P-value was reported.) | NR | NR | NR |
| EXPEDITION1 [17] | Solanezumab | Antibody:506  Placebo:506 | Change from baseline to 4,8,12,28,52,64 and 80weeks after randomization. | MMRM including baseline plasma Aβ, investigator, treatment, visit, treatment-by-visit interaction, and age at baseline. | NR | ↑  P<0.001  (Only P-value was reported.) | ↑  P<0.001  (Only P-value was reported.) | NR | NR | NR |
| EXPEDITION2 [17] | Solanezumab | Antibody:521  Placebo: 519 | Change from baseline to 4,8,12,28,52,64 and 80weeks after randomization. | MMRM including baseline plasma Aβ, investigator, treatment, visit, treatment-by-visit interaction, and age at baseline. | NR | ↑  P<0.001  Only P-value was reported) | ↑  P<0.001  Only P-value was reported) | NR | NR | NR |
| Study 3000 [32] | Bapineuzumab | Antibody: 28  Placebo: 31 | Change from baseline to week 71. | Not clearly described. | ↑  Pooled difference, 941.86pg/mL, P<0.001  Line graphs are provided. | NR | NR | NR | NR | NR |
| Study 3001 [32] | Bapineuzumab | Antibody:18  Placebo: 58 | Change from baseline to week 71. | Not clearly described. | ↑  Difference: 827.29 pg/mL, P<0.001  Line graphs are provided. | NR | NR | NR | NR | NR |
| Study 301 [33] | Bapineuzumab | NR | NR | NR | NR | NR | NR | NR | NR | NR |
| Study 302 [33] | Bapineuzumab | NR | NR | NR | NR | NR | NR | NR | NR | NR |

“↑” indicates that the level of the biomarker is significantly increased in the treatment (antibody) group compared to that of placebo group. “↓” indicates that the level of biomarker is significantly decrease in the treatment (antibody) group compared to the placebo group.

Aβ: Amyloid beta, MMRM: Mixed-effects model for repeated measures. NR: not reported, N.S: non-significant.

## **Table G. Number needed to treat/harm**

|  | **RD (95%CI)** | **Number needed to treat/harm** |
| --- | --- | --- |
| CDR-SB | -0.14 (-0.58,0.31) | 8 |
| ADAS-Cog | -0.28 (-0.71, 0.15) | 4 |
| Death | 0.001 (-0.002, 0.003) | NA* |
| Serious adverse events | 0.003 (-0.01, 0.02) | NA* |
| ARIA-E | 0.15 (0.10, 0.21) | 7 |
| ARIA-H | 0.10 (0.06, 0.14) | 10 |
| Headache | 0.02 (0.01, 0.03) | 54 |
| Fall | 0.003 (-0.01, 0.01) | NA* |
| Dizziness | 0.005 (-0.004, 0.01) | NA* |
| Cerebral microhemorrhage | 0.002 (0.000, 0.004) | NA* |

*For the secondary outcomes, number needed to harm is reported when the result of main analysis is statistically significant.

RD: risk difference, ARIA-E: amyloid-related imaging abnormalities with edema or effusion, ARIA-H: amyloid-related imaging abnormalities with hemorrhage, NA: not applicable.
