# Supplementary figures and images for: Efficacy and safety of passive immunotherapies targeting amyloid beta in Alzheimer’s disease: A systematic review and meta-analysis
Source: PLoS Med. 2025 Mar 31;22(3):e1004568. doi: 10.1371/journal.pmed.1004568 (PMC12002640; doi:10.1371/journal.pmed.1004568)

(a) CDR-SB

Egger's test  $P=0.04^*$

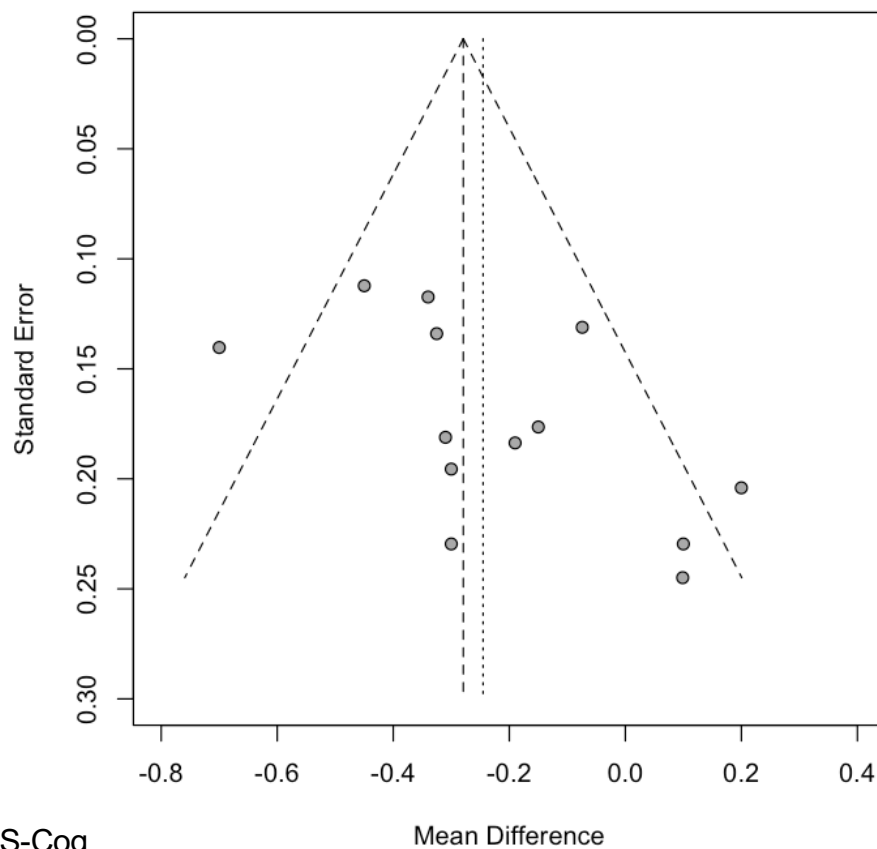

(b) ADAS-Cog

Egger's test  $P=0.75$

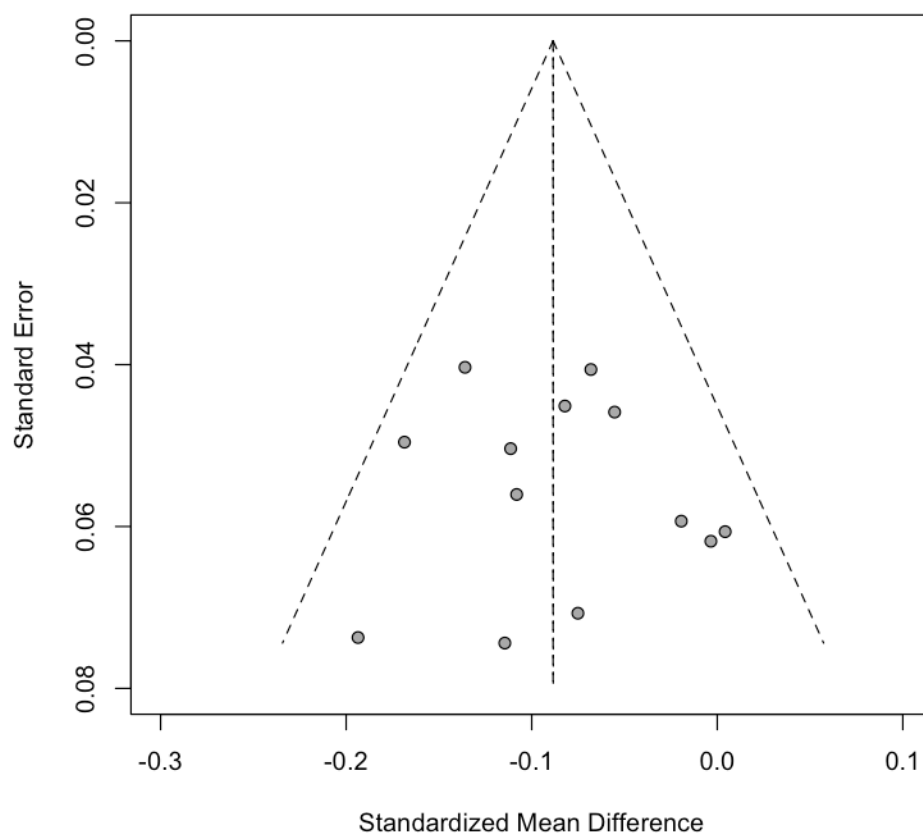

S1 Figure: Funnel plots for efficacy endpoints.

Supplement: S1 Fig — (a) The Clinical Dementia Rating-Sum of Boxes (CDR-SB) and (b) Alzheimer’s Disease Assessment Scale-Cognitive Subscale (ADAS-Cog). Filled circles represent estimated treatment effect (risk ratio) and its precision (standard error) for each individual study. In addition to individual study results, the fixed effect estimate (vertical dashed line) with 95% confidence interval limits (diagonal dashed lines) and the random effects estimate (vertical dotted line) are shown in the figures. Also, P-values of Egger’s test are shown. *P-value < 0.05. (PDF) [file pmed.1004568.s002.pdf]

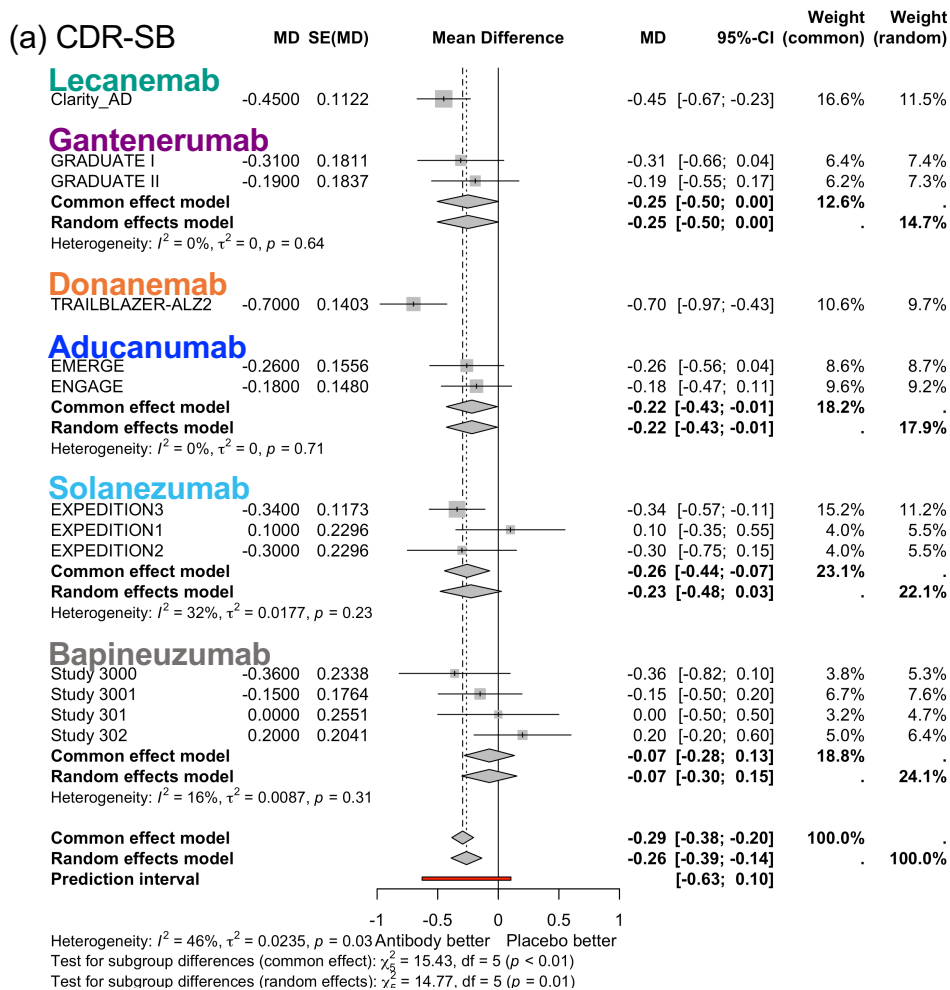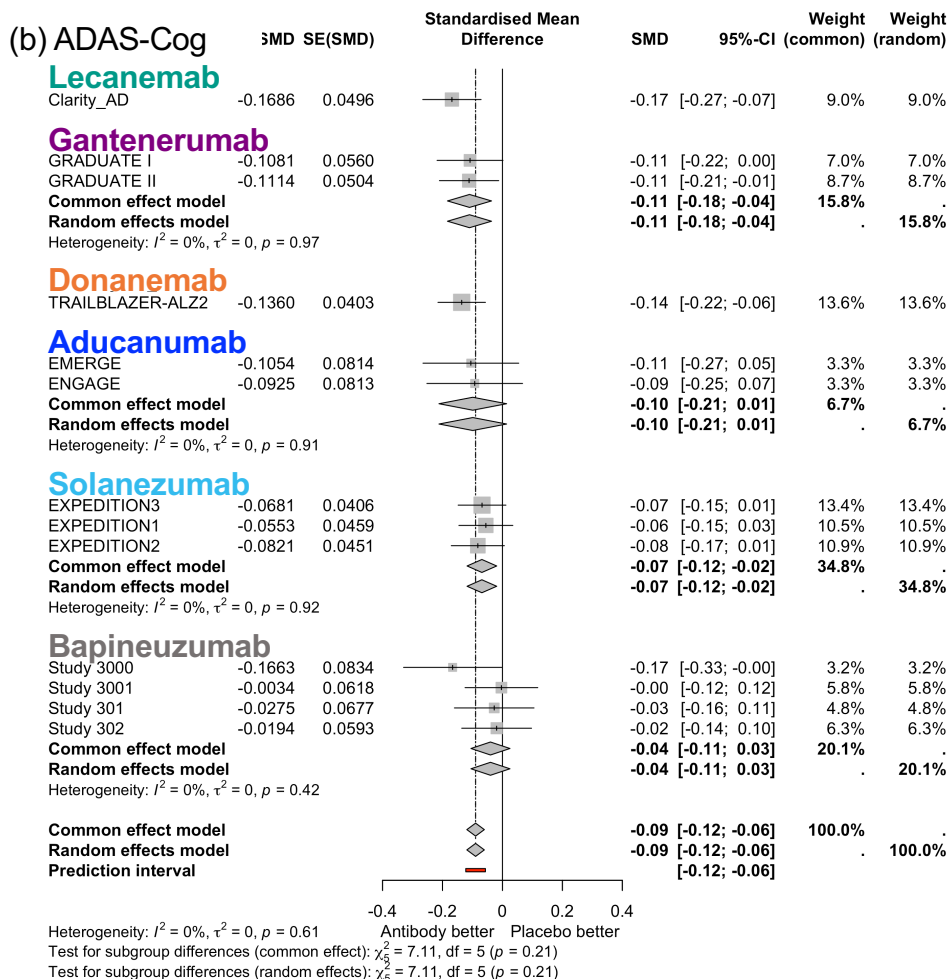

S37 Figure: Forest plots for efficacy outcomes (low-dose populations).

Supplement: S37 Fig — (a) The Clinical Dementia Rating-Sum of Boxes (CDR-SB) and (b) Alzheimer’s Disease Assesment Scale-Cognitive Subscale (ADAS-Cog). (PDF) [file pmed.1004568.s038.pdf]

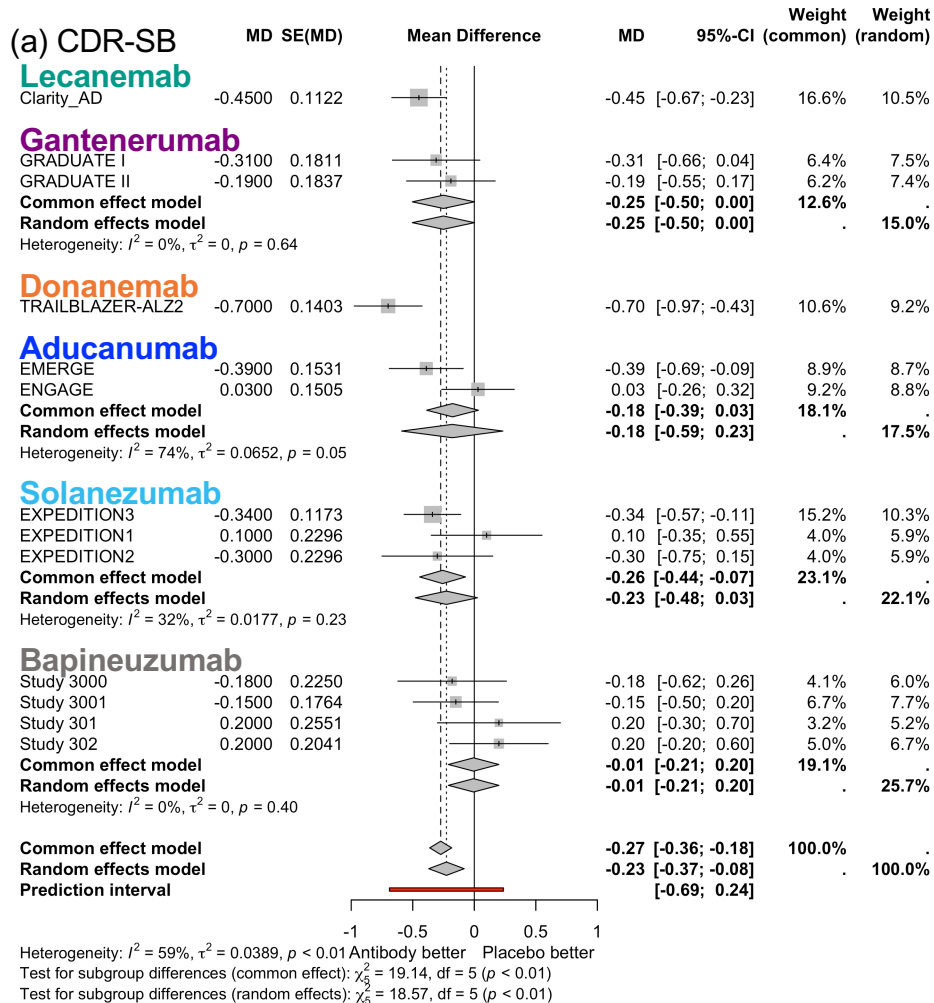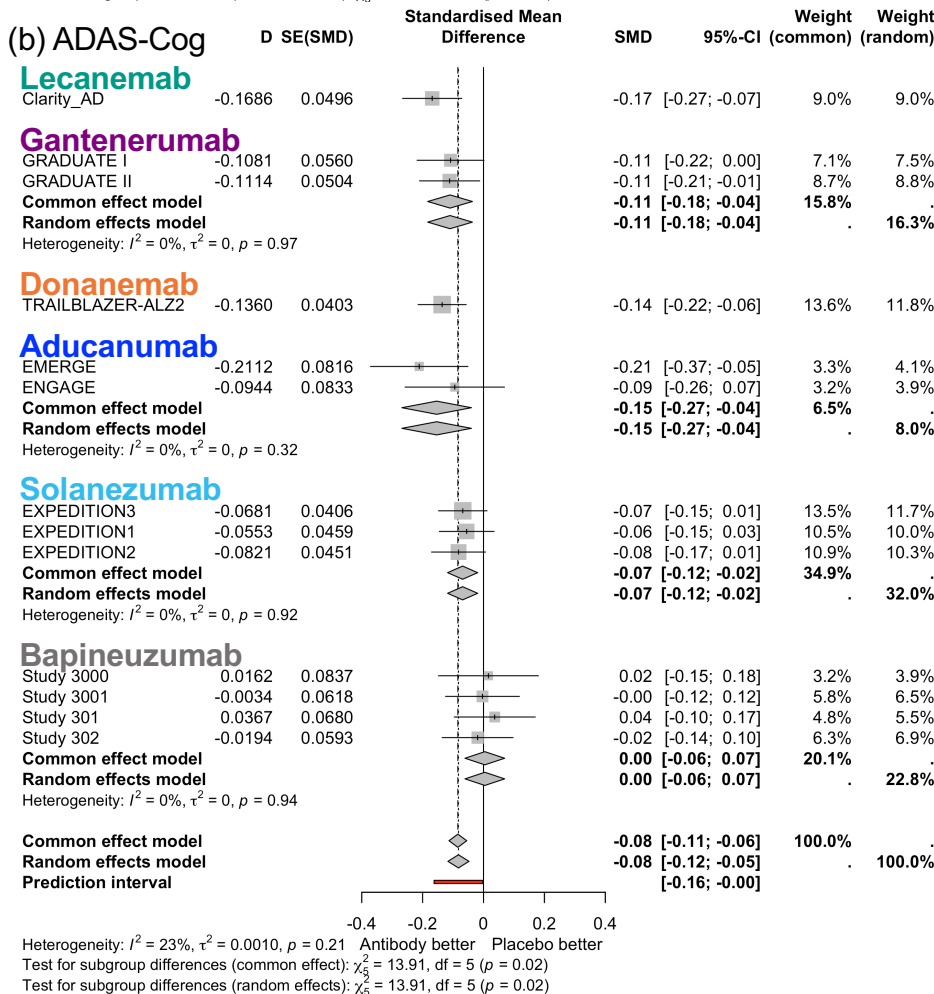

S41 Figure: Forest plots for efficacy outcomes (high-dose populations).

Supplement: S41 Fig — (a) The Clinical Dementia Rating-Sum of Boxes (CDR-SB) and (b) Alzheimer’s Disease Assesment Scale-Cognitive Subscale (ADAS-Cog). (PDF) [file pmed.1004568.s042.pdf]
