# Supplementary material for: Efficacy and safety of passive immunotherapies targeting amyloid beta in Alzheimer’s disease: A systematic review and meta-analysis
Source: PLoS Med. 2025 Mar 31;22(3):e1004568. doi: 10.1371/journal.pmed.1004568 (PMC12002640; doi:10.1371/journal.pmed.1004568)
Supplement: S3 Fig — The size of the bubbles shows the inverse of the variance of the mean difference in each trial, with larger bubbles indicating trials with higher precision. The P-values from the meta-regression analysis are also reported. CDR-SB, the Clinical Dementia Rating-Sum of Boxes; MMSE, Mini-Mental State Examination; PET, positron emission tomography. (PDF) [file pmed.1004568.s004.pdf]

CDR-SB

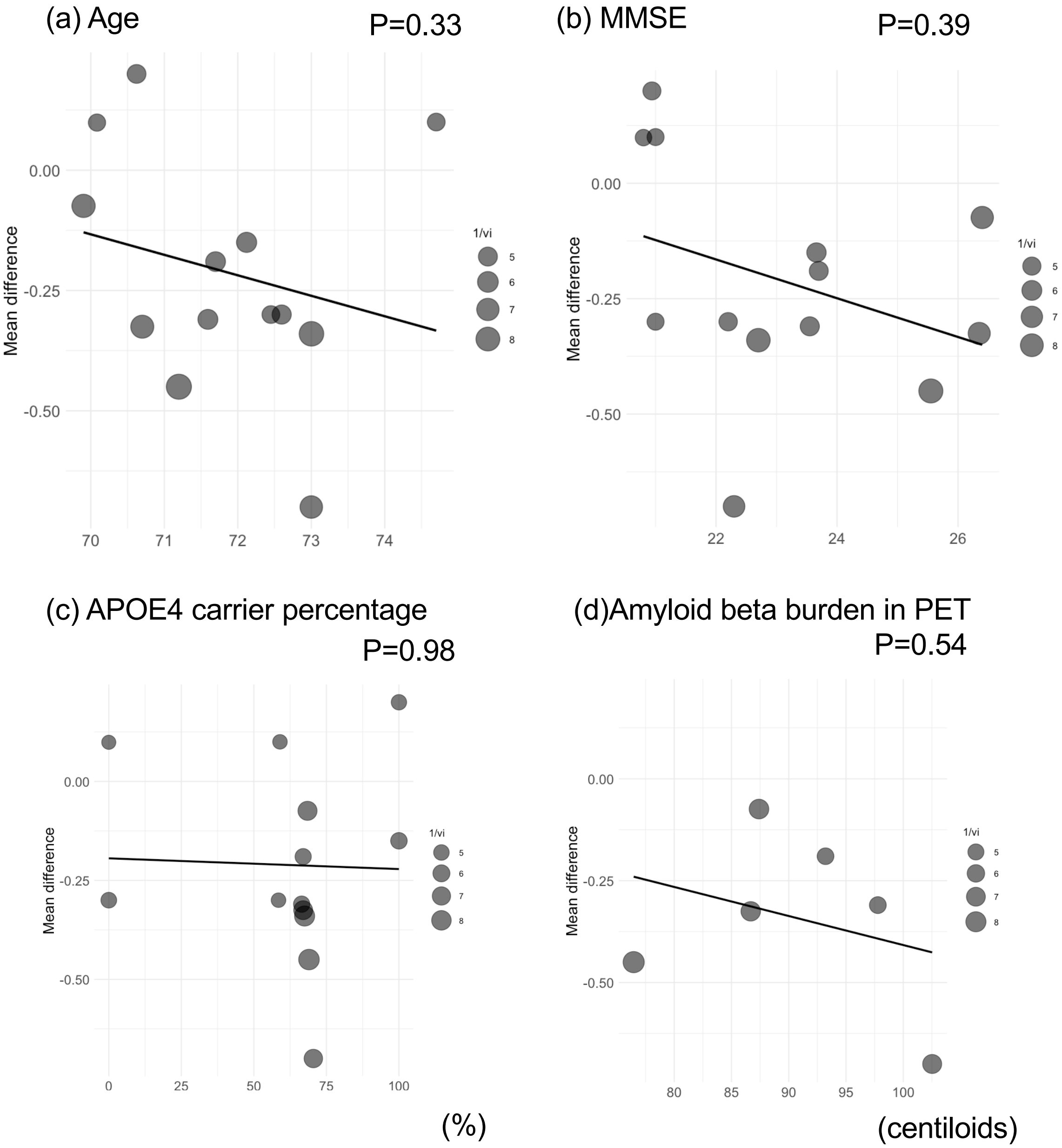

S3 Figure: Meta-regression of CDR-SB mean difference by (a)mean age, (b)MMSE score, (c)ApoE4 carrier percentage, and (d)amyloid beta burden in PET.
