# Supplementary material for: Efficacy and safety of passive immunotherapies targeting amyloid beta in Alzheimer’s disease: A systematic review and meta-analysis
Source: PLoS Med. 2025 Mar 31;22(3):e1004568. doi: 10.1371/journal.pmed.1004568 (PMC12002640; doi:10.1371/journal.pmed.1004568)
Supplement: S7 Fig — (PDF) [file pmed.1004568.s008.pdf]

Serious Adverse Event

Study	Experimental Events	Experimental Total	Control Events	Control Total	RR	95%-CI	Weight (common)	Weight (random)
GRADUATE I	76	503	95	481	0.77	[0.58; 1.01]	7.5%	8.2%
GRADUATE II	61	501	63	474	0.92	[0.66; 1.27]	5.0%	6.4%
TRAILBLAZER-ALZ2	148	853	138	874	1.10	[0.89; 1.36]	10.5%	10.9%
Clarity_AD	126	898	101	897	1.25	[0.98; 1.59]	7.8%	9.3%
EMERGE_high	145	1091	81	547	0.90	[0.70; 1.15]	8.3%	9.1%
ENGAGE_high	155	1107	70	540	1.08	[0.83; 1.40]	7.3%	8.6%
EXPEDITION3	175	1054	202	1067	0.88	[0.73; 1.05]	15.5%	12.4%
EXPEDITION1 & 2	261	1027	263	1025	0.99	[0.85; 1.15]	20.4%	14.5%
Study 301	148	666	108	524	1.08	[0.87; 1.34]	9.3%	10.5%
Study 302	176	673	89	448	1.32	[1.05; 1.65]	8.3%	10.2%
**Common effect model**		**8373**		**6877**	**1.02**	**[0.95; 1.09]**	**100.0%**	**.**
**Random effects model**					**1.02**	**[0.92; 1.13]**	**.**	**100.0%**
**Prediction interval**						**[0.77; 1.35]**		

Heterogeneity: $I^2 = 46\%$, $\tau^2 = 0.0124$, $p = 0.05$

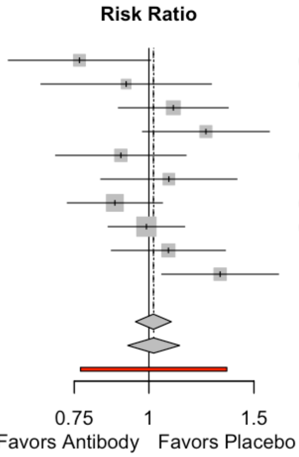

Gantenerumab Donanemab Lecanemab Aducanumab Solanezumab Beprineuzumab

S7 Figure: Forest plot showing the results of meta-analysis for serious adverse events.
