# Supplementary material for: Efficacy and safety of passive immunotherapies targeting amyloid beta in Alzheimer’s disease: A systematic review and meta-analysis
Source: PLoS Med. 2025 Mar 31;22(3):e1004568. doi: 10.1371/journal.pmed.1004568 (PMC12002640; doi:10.1371/journal.pmed.1004568)
Supplement: S9 Fig — The size of the bubbles shows the inverse of the variance of the log-transformed risk ratio in each trial, with larger bubbles indicating trials with higher precision. The p-values for comparison with the reference group (shown as “ref”) from the meta-regression analysis are also reported on the top of the bubble plots. AD, Alzheimer’s Disease. (PDF) [file pmed.1004568.s010.pdf]

Death

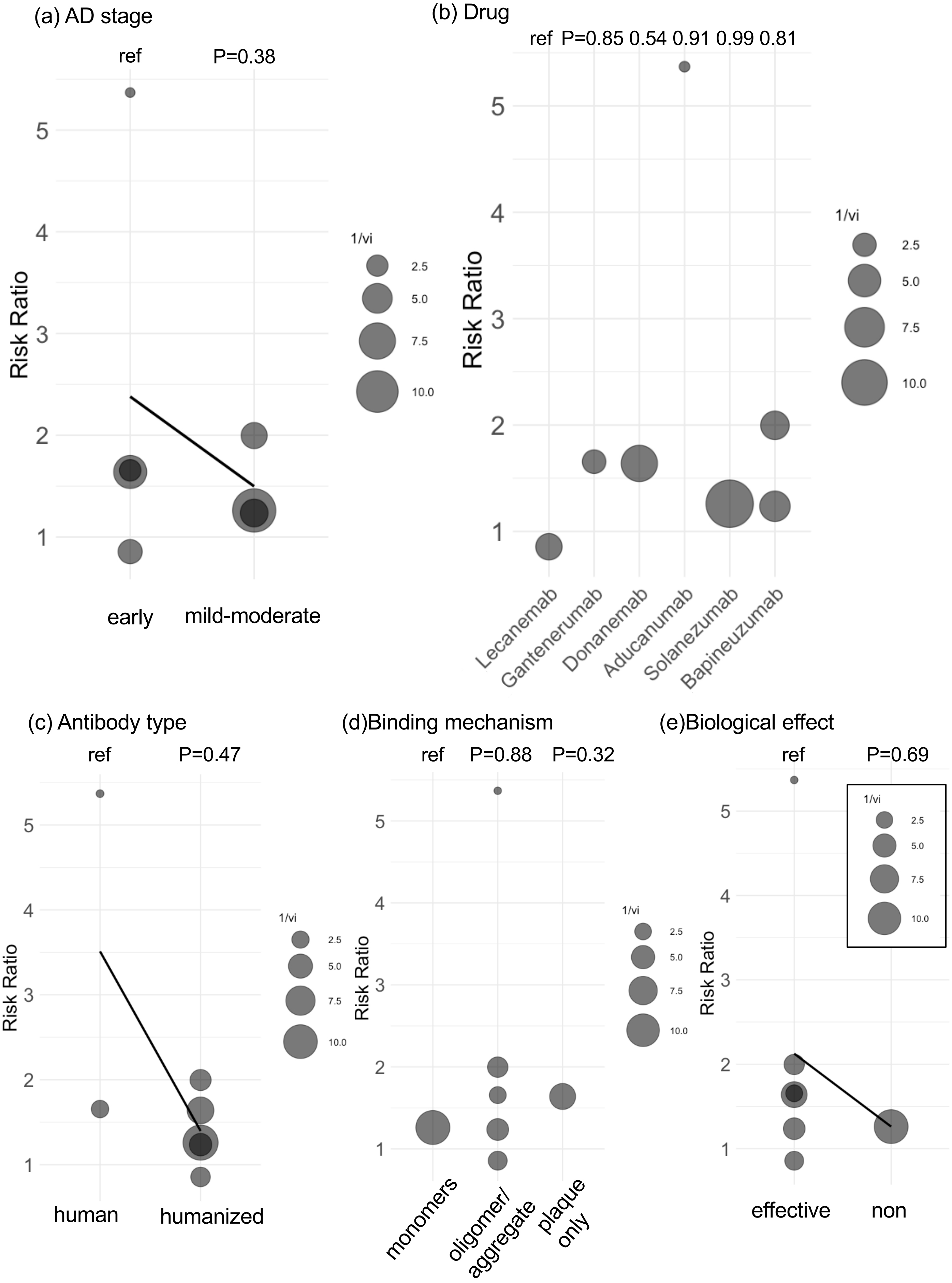

S9 Figure: Bubble plot showing the results of meta-regression of death, by (a)AD stage, (b)Drug, (c)Antibody type, (d)binding mechanism, and (e) biological effect.
