# Supplementary material for: Efficacy and safety of passive immunotherapies targeting amyloid beta in Alzheimer’s disease: A systematic review and meta-analysis
Source: PLoS Med. 2025 Mar 31;22(3):e1004568. doi: 10.1371/journal.pmed.1004568 (PMC12002640; doi:10.1371/journal.pmed.1004568)
Supplement: S13 Fig — The size of the bubbles shows the inverse of the variance of the log-transformed risk ratio in each trial, with larger bubbles indicating trials with higher precision. The p-values for comparison with the reference group (shown as “ref”) from the meta-regression analysis are also reported on the top of the bubble plots. ARIA-E, amyloid-related imaging abnormalities with edema; AD, Alzheimer’s Disease. (PDF) [file pmed.1004568.s014.pdf]

ARIA-E

(a) AD stage

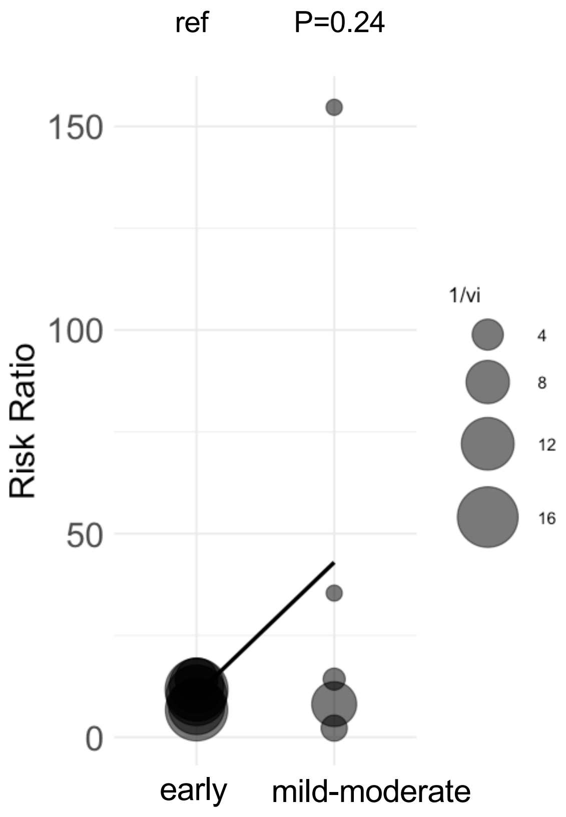

(b) Drug

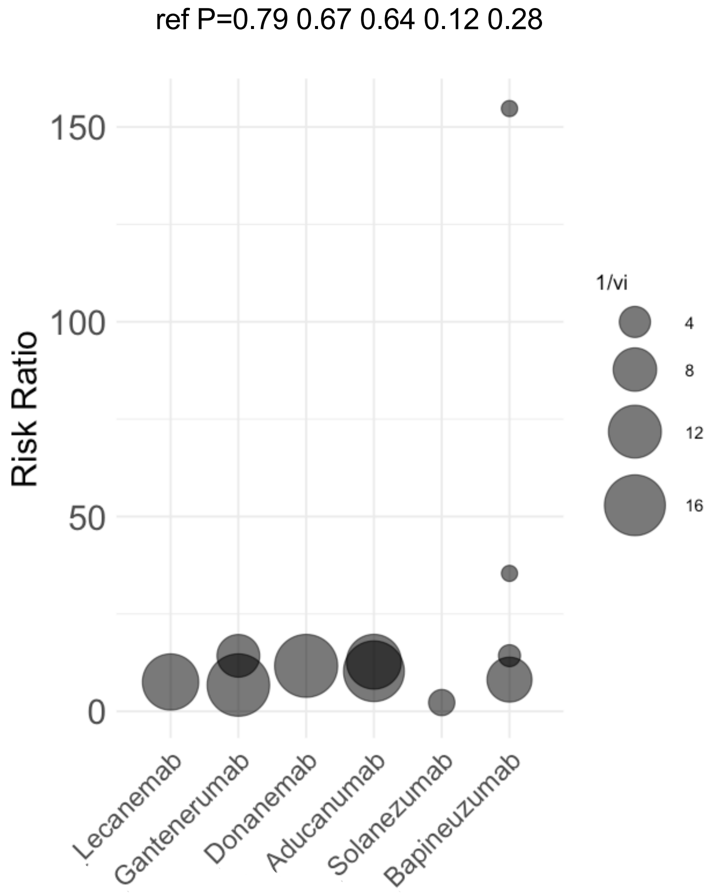

(c) Antibody type

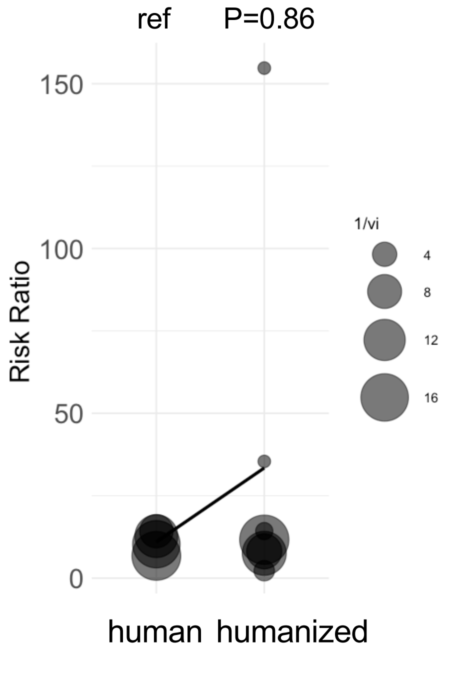

S13 Figure: Bubble plots showing the results of meta-regression of the occurrence of ARIA-E, by (a)AD stage, (b)drug, and (c)antibody type.
