# Supplementary material for: Efficacy and safety of passive immunotherapies targeting amyloid beta in Alzheimer’s disease: A systematic review and meta-analysis
Source: PLoS Med. 2025 Mar 31;22(3):e1004568. doi: 10.1371/journal.pmed.1004568 (PMC12002640; doi:10.1371/journal.pmed.1004568)
Supplement: S14 Fig — The size of the bubbles shows the inverse of the variance of the log-transformed risk ratio in each trial, with larger bubbles indicating trials with higher precision. The bubble plots on the left includes all trials, while the bubble plots on the right shows only trials with a risk ratio of up to 50. The p-values for comparison with the reference group (shown as “ref”) from the meta-regression analysis are also reported on the top of the bubble plots. *P-value < 0.05. ARIA-E, amyloid-related imaging abnormalities with edema; AD, Alzheimer’s Disease. (PDF) [file pmed.1004568.s015.pdf]

ARIA-E

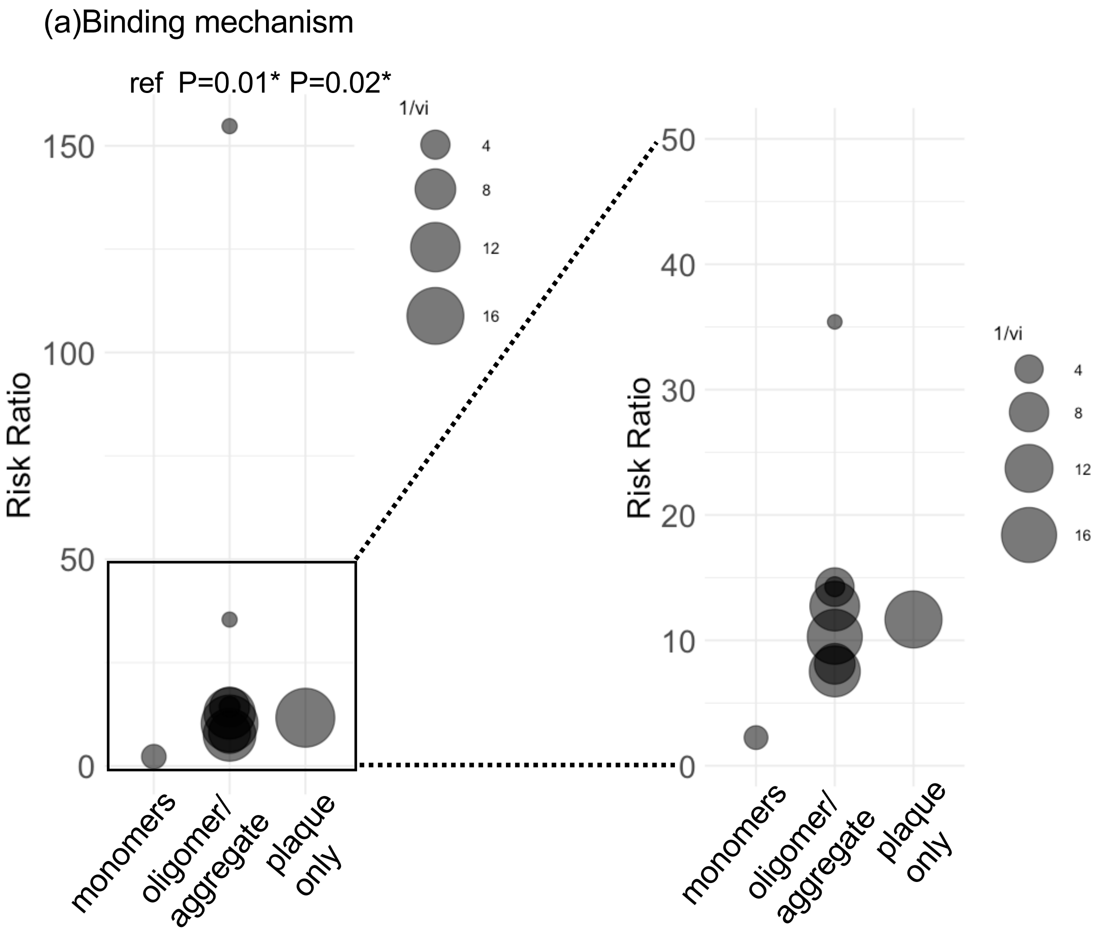

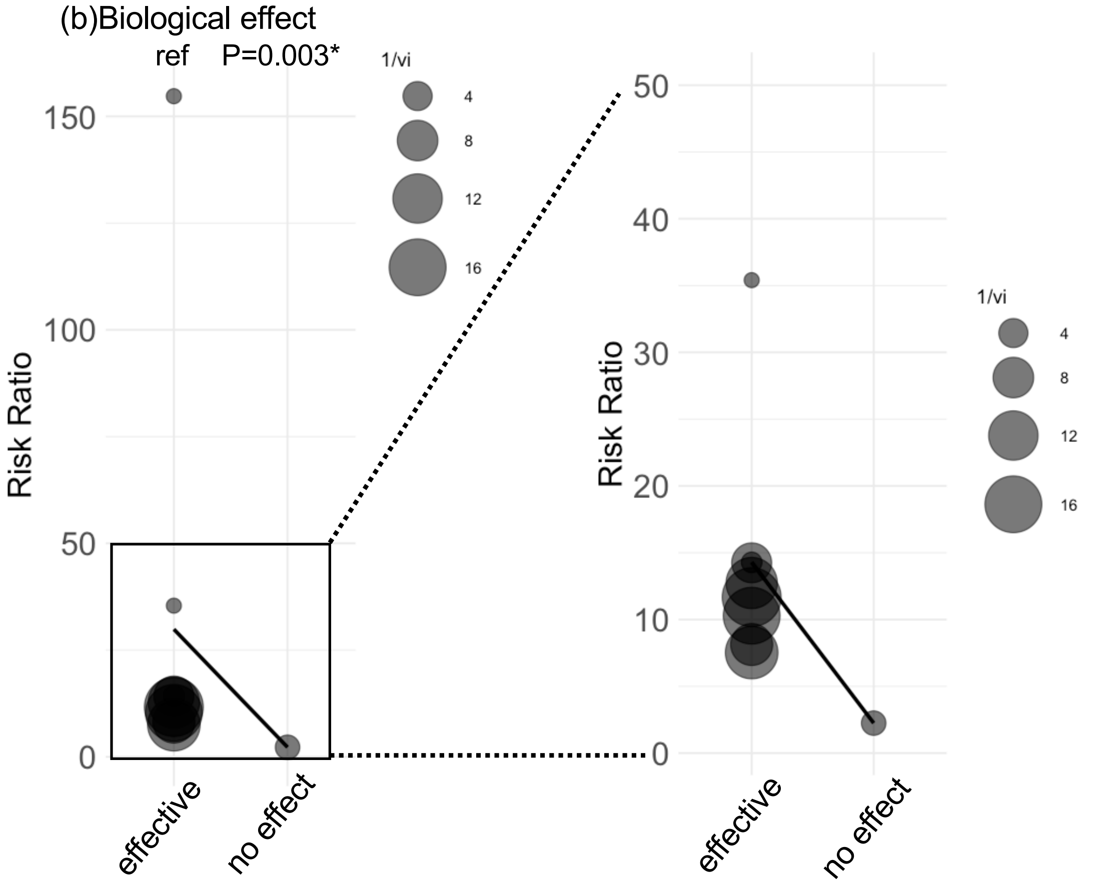

S14 Figure: Bubble plots showing the results of meta-regression of the occurrence of ARIA-E, by (a)binding mechanism and (b) biological effect.
