# Supplementary material for: Efficacy and safety of passive immunotherapies targeting amyloid beta in Alzheimer’s disease: A systematic review and meta-analysis
Source: PLoS Med. 2025 Mar 31;22(3):e1004568. doi: 10.1371/journal.pmed.1004568 (PMC12002640; doi:10.1371/journal.pmed.1004568)
Supplement: S15 Fig — The size of the bubbles shows the inverse of the variance of the log-transformed risk ratio in each trial, with larger bubbles indicating trials with higher precision. The p-values from the meta-regression analysis are also reported. MMSE, Mini-Mental State Examination; PET, positron emission tomography. (PDF) [file pmed.1004568.s016.pdf]

Headache

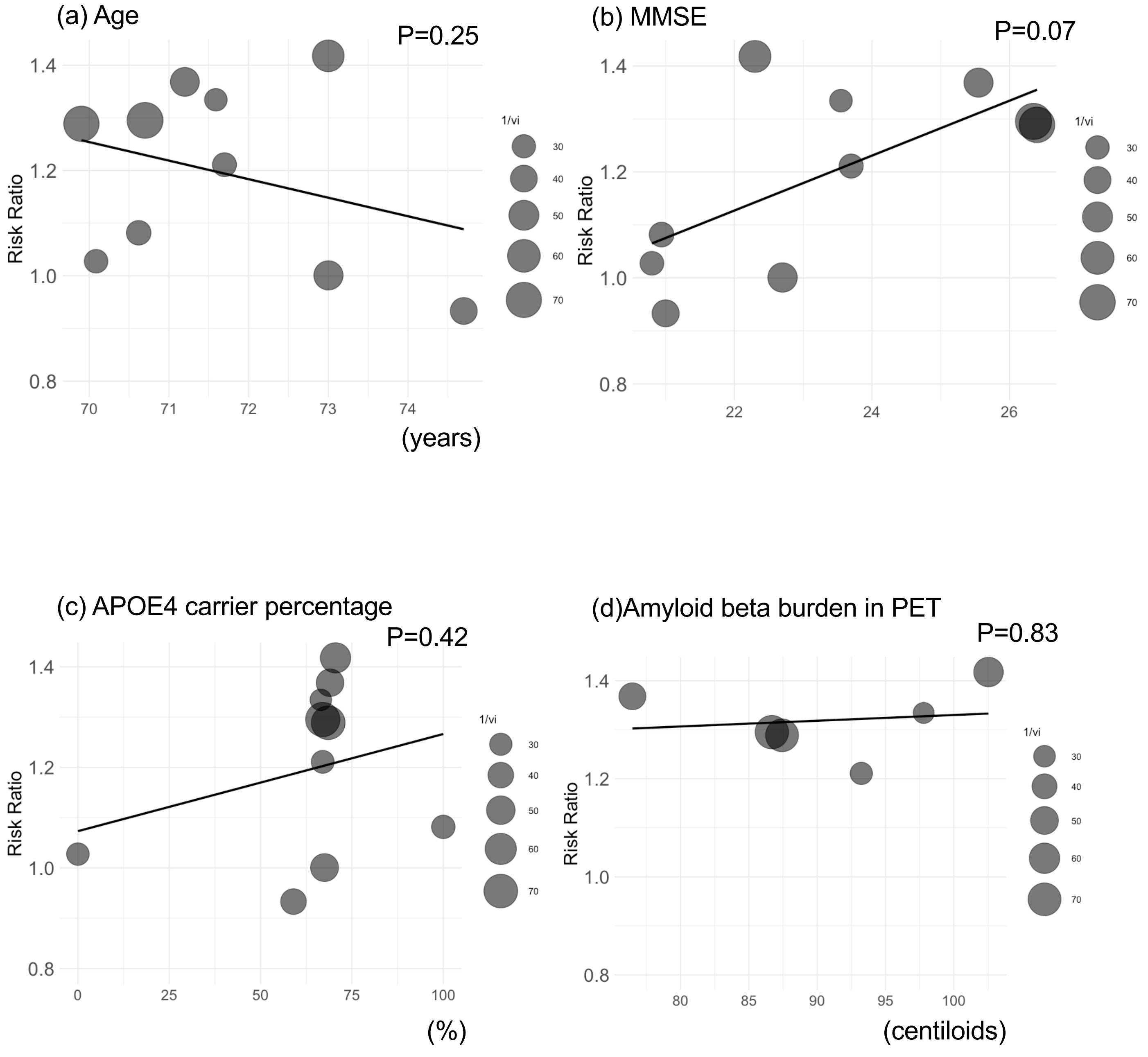

S15 Figure: Bubble plots showing the results of meta-regression of the occurrence of headache, by (a)mean age, (b)MMSE score, (c)ApoE4 carrier percentage, and (d)amyloid beta burden in PET.
