# Supplementary material for: Efficacy and safety of passive immunotherapies targeting amyloid beta in Alzheimer’s disease: A systematic review and meta-analysis
Source: PLoS Med. 2025 Mar 31;22(3):e1004568. doi: 10.1371/journal.pmed.1004568 (PMC12002640; doi:10.1371/journal.pmed.1004568)
Supplement: S21 Fig — (PDF) [file pmed.1004568.s022.pdf]

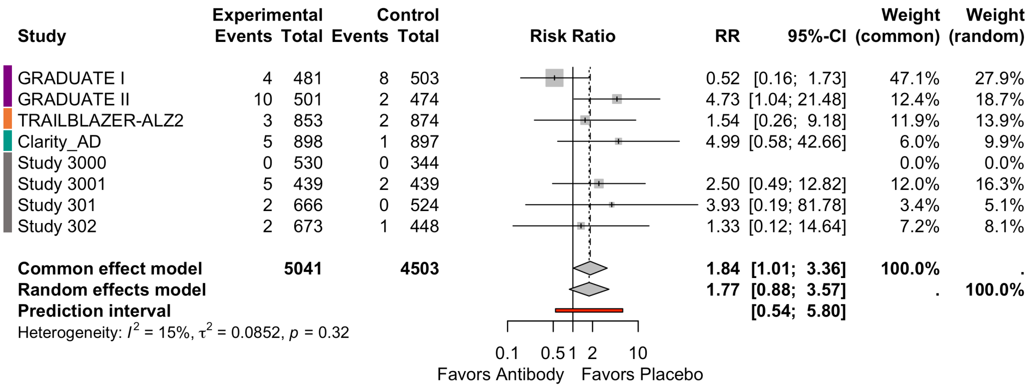

Ganntenerumab Donanemab Lecanemab Beprineuzumab

S21 Figure: Forest plot for cerebral macrohemorrhage.
