# Supplementary material for: Efficacy and safety of passive immunotherapies targeting amyloid beta in Alzheimer’s disease: A systematic review and meta-analysis
Source: PLoS Med. 2025 Mar 31;22(3):e1004568. doi: 10.1371/journal.pmed.1004568 (PMC12002640; doi:10.1371/journal.pmed.1004568)
Supplement: S23 Fig — (a) Death, (b) Serious Adverse Event, (c) ARIA-E, (d) ARIA-H. ARIA-E: Amyloid-Related Imaging Abnormalities-Effusion, ARIA-H, Amyloid-Related Imaging Abnormalities-Hemorrhage. (PDF) [file pmed.1004568.s024.pdf]

(a) Death

Study	Experimental Events	Experimental Total	Control Events	Control Total	RR	95%-CI	Weight (common)	Weight (random)
GRADUATE I	3	503	10	481	0.29	[0.08; 1.04]	16.8%	8.7%
GRADUATE II	7	501	4	474	1.66	[0.49; 5.62]	6.8%	9.5%
TRAILBLAZER-ALZ2	16	853	10	874	1.64	[0.75; 3.59]	16.2%	18.4%
Clarity_AD	6	898	7	897	0.86	[0.29; 2.54]	11.5%	11.5%
EMERGE_high	6	1091	5	547	0.60	[0.18; 1.96]	11.0%	10.0%
ENGAGE_high	5	1107	0	540	5.37	[0.30; 96.91]	1.1%	2.0%
Study 3000	2	530	3	344	0.43	[0.07; 2.58]	6.0%	4.9%
Study 3001	4	654	4	439	0.67	[0.17; 2.67]	7.9%	7.7%
Study 301	11	666	7	524	1.24	[0.48; 3.17]	12.9%	14.3%
Study 302	15	673	5	448	2.00	[0.73; 5.46]	9.9%	12.9%
**Common effect model**		**7476**		**5568**	**1.09**	**[0.77; 1.54]**	**100.0%**	.
**Random effects model**					**1.04**	**[0.69; 1.58]**	.	**100.0%**
**Prediction interval**						**[0.46; 2.38]**		

Heterogeneity: $I^2 = 18\%$, $\tau^2 = 0.0833$, $p = 0.28$

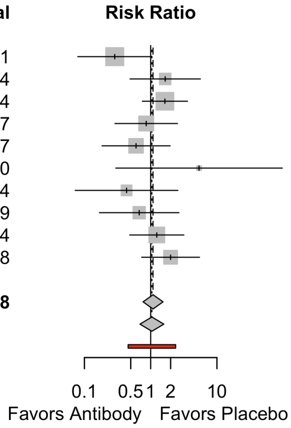

(b) Serious adverse events

Study	Experimental Events	Experimental Total	Control Events	Control Total	RR	95%-CI	Weight (common)	Weight (random)
GRADUATE I	76	503	95	481	0.77	[0.58; 1.01]	11.7%	11.3%
GRADUATE II	61	501	63	474	0.92	[0.66; 1.27]	7.8%	9.1%
TRAILBLAZER-ALZ2	148	853	138	874	1.10	[0.89; 1.36]	16.4%	14.6%
Clarity_AD	126	898	101	897	1.25	[0.98; 1.59]	12.2%	12.8%
EMERGE_high	145	1091	81	547	0.90	[0.70; 1.15]	13.0%	12.4%
ENGAGE_high	155	1107	70	540	1.08	[0.83; 1.40]	11.4%	11.8%
Study 301	148	666	108	524	1.08	[0.87; 1.34]	14.6%	14.2%
Study 302	176	673	89	448	1.32	[1.05; 1.65]	12.9%	13.9%
**Common effect model**		**6292**		**4785**	**1.06**	**[0.97; 1.16]**	**100.0%**	.
**Random effects model**					**1.05**	**[0.93; 1.19]**	.	**100.0%**
**Prediction interval**						**[0.75; 1.47]**		

Heterogeneity: $I^2 = 47\%$, $\tau^2 = 0.0150$, $p = 0.07$

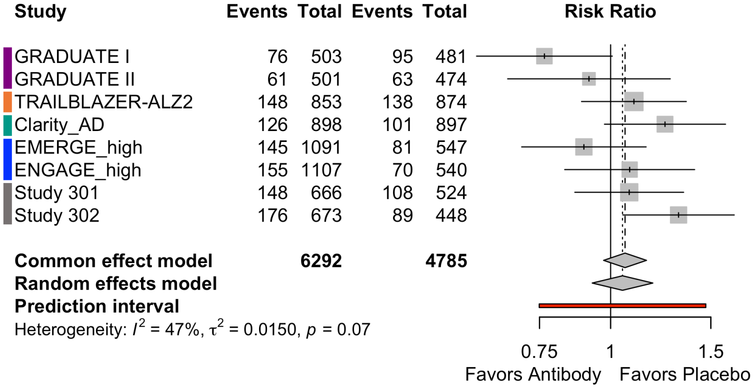

(c) ARIA-E

Study	Experimental Events	Experimental Total	Control Events	Control Total	RR	95%-CI	Weight (common)	Weight (random)
GRADUATE I	119	497	8	476	14.25	[7.04; 28.83]	7.2%	10.8%
GRADUATE II	128	496	18	470	6.74	[4.18; 10.86]	16.3%	13.7%
TRAILBLAZER-ALZ2	205	853	18	874	11.67	[7.28; 18.72]	15.7%	13.8%
Clarity_AD	113	898	15	897	7.52	[4.43; 12.79]	13.2%	13.0%
EMERGE_high	328	1078	13	544	12.73	[7.39; 21.95]	15.2%	12.8%
ENGAGE_high	340	1099	16	532	10.29	[6.30; 16.80]	19.0%	13.5%
Study 3000	44	530	2	344	14.28	[3.48; 58.52]	2.1%	5.0%
Study 3001	109	654	9	439	8.13	[4.16; 15.87]	9.5%	11.3%
Study 301	45	666	1	524	35.41	[4.90; 256.00]	1.0%	3.0%
Study 302	103	448	1	673	154.73	[21.67; 1104.90]	0.7%	3.0%
**Common effect model**		**7219**		**5773**	**11.36**	**[9.32; 13.85]**	**100.0%**	.
**Random effects model**					**11.24**	**[7.74; 16.31]**	.	**100.0%**
**Prediction interval**						**[3.63; 34.80]**		

Heterogeneity: $I^2 = 42\%$, $\tau^2 = 0.2040$, $p = 0.08$

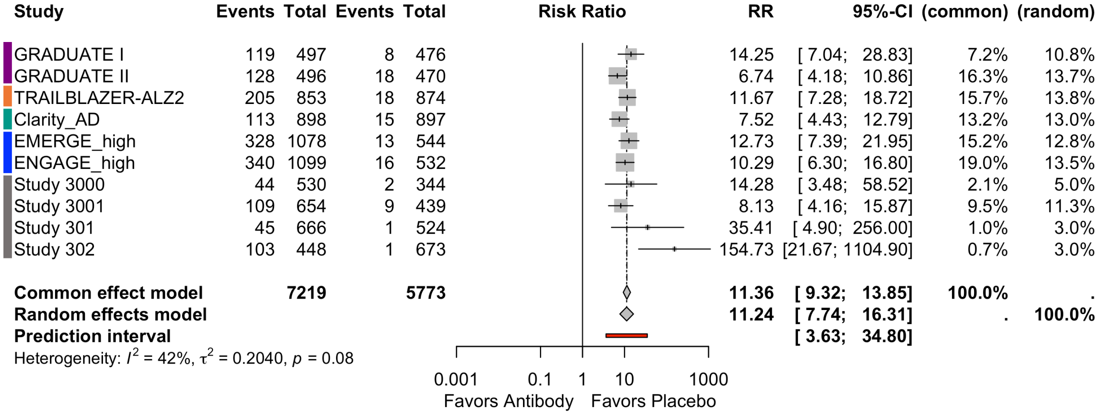

(d) ARIA-H

Study	Experimental Events	Experimental Total	Control Events	Control Total	RR	95%-CI	Weight (common)	Weight (random)
GRADUATE I	118	497	59	476	1.92	[1.44; 2.55]	14.6%	16.0%
GRADUATE II	109	496	57	470	1.81	[1.35; 2.43]	14.2%	15.3%
TRAILBLAZER-ALZ2	268	853	119	874	2.31	[1.90; 2.80]	28.5%	25.2%
Clarity_AD	155	898	81	897	1.91	[1.49; 2.46]	19.7%	18.8%
EMERGE	195	1078	37	544	2.66	[1.90; 3.72]	11.9%	12.7%
ENGAGE	198	1099	34	532	2.82	[1.99; 4.00]	11.1%	12.0%
**Common effect model**		**4921**		**3793**	**2.20**	**[1.97; 2.46]**	**100.0%**	**--**
**Random effects model**					**2.17**	**[1.89; 2.50]**	**--**	**100.0%**
**Prediction interval**						**[1.55; 3.05]**		

Heterogeneity: $I^2 = 30\%$, $\tau^2 = 0.0099$, $p = 0.21$

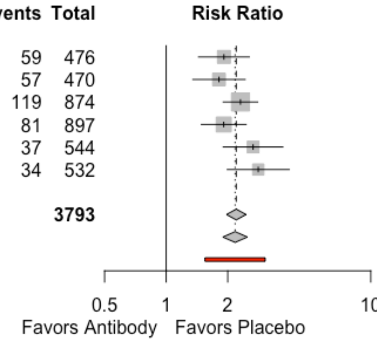

Gantenerumab **Donanemab** **Lecanemab** **Aducanumab** **Beprineuzumab**

S23 Figure: Sensitivity analysis 1 (excluding solanezumab). Forest plots for safety outcomes.
