# Supplementary material for: Efficacy and safety of passive immunotherapies targeting amyloid beta in Alzheimer’s disease: A systematic review and meta-analysis
Source: PLoS Med. 2025 Mar 31;22(3):e1004568. doi: 10.1371/journal.pmed.1004568 (PMC12002640; doi:10.1371/journal.pmed.1004568)
Supplement: S24 Fig — (a) Headache, (b) Fall, and (c) Dizziness. (PDF) [file pmed.1004568.s025.pdf]

(a) Headache

Study	Experimental Events	Experimental Total	Control Events	Control Total	RR	95%-CI	Weight (common)	Weight (random)
GRADUATE I	60	503	43	481	1.33	[0.92; 1.93]	7.5%	7.5%
GRADUATE II	64	501	50	474	1.21	[0.86; 1.72]	8.8%	8.6%
TRAILBLAZER-ALZ2	119	853	86	874	1.42	[1.09; 1.84]	14.5%	15.3%
Clarity_AD	100	898	73	897	1.37	[1.03; 1.82]	12.5%	12.6%
EMERGE_high	217	1091	84	547	1.30	[1.03; 1.63]	19.1%	19.7%
ENGAGE_high	214	1107	81	540	1.29	[1.02; 1.63]	18.6%	19.0%
Study 301	64	666	49	524	1.03	[0.72; 1.46]	9.3%	8.3%
Study 302	78	673	48	448	1.08	[0.77; 1.52]	9.8%	9.0%
**Common effect model**		**6292**		**4785**	**1.27**	**[1.15; 1.41]**	**100.0%**	**.**
**Random effects model**					**1.27**	**[1.15; 1.41]**	**.**	**100.0%**
**Prediction interval**						**[1.12; 1.44]**		

Heterogeneity: $I^2 = 0\%$, $\tau^2 = 0$, $p = 0.85$

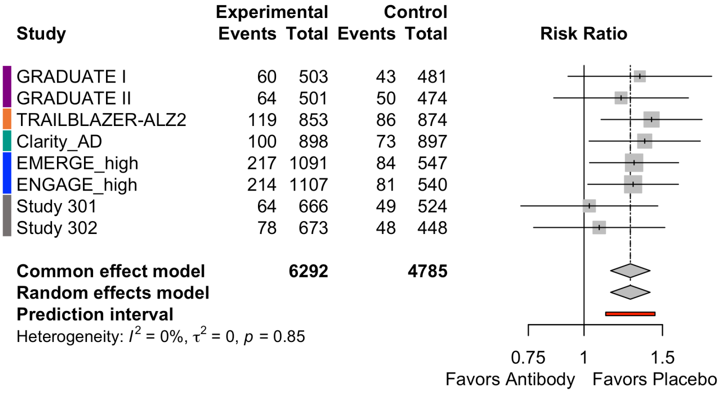

(b) Fall

Study	Experimental Events	Experimental Total	Control Events	Control Total	RR	95%-CI	Weight (common)	Weight (random)
GRADUATE I	63	503	62	481	0.97	[0.70; 1.35]	9.9%	9.6%
GRADUATE II	50	501	52	474	0.91	[0.63; 1.31]	8.3%	7.6%
TRAILBLAZER-ALZ2	114	853	110	874	1.06	[0.83; 1.36]	16.9%	17.3%
Clarity_AD	93	898	86	897	1.08	[0.82; 1.43]	13.4%	13.3%
EMERGE_high	144	1091	71	547	1.02	[0.78; 1.33]	14.7%	14.7%
ENGAGE_high	166	1107	57	540	1.42	[1.07; 1.88]	11.9%	12.9%
Study 301	86	666	73	524	0.93	[0.69; 1.24]	12.7%	12.3%
Study 302	100	673	64	448	1.04	[0.78; 1.39]	12.0%	12.3%
**Common effect model**		**6292**		**4785**	**1.06**	**[0.96; 1.17]**	**100.0%**	**.**
**Random effects model**					**1.06**	**[0.95; 1.17]**	**.**	**100.0%**
**Prediction interval**						**[0.93; 1.20]**		

Heterogeneity: $I^2 = 0\%$, $\tau^2 = 0$, $p = 0.54$

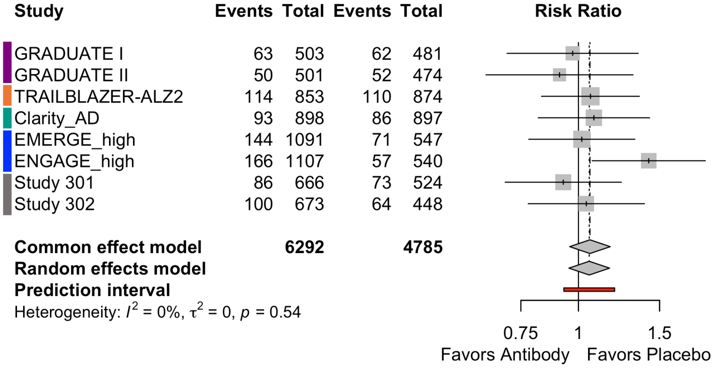

(c) Dizziness

Study	Experimental Events	Experimental Total	Control Events	Control Total	RR	95%-CI	Weight (common)	Weight (random)
GRADUATE I	45	503	41	481	1.05	[0.70; 1.57]	12.6%	12.6%
GRADUATE II	39	501	29	474	1.27	[0.80; 2.02]	8.9%	9.6%
TRAILBLAZER-ALZ2	53	853	48	874	1.13	[0.77; 1.65]	14.2%	14.3%
Clarity_AD	49	898	46	897	1.06	[0.72; 1.57]	13.8%	13.4%
EMERGE_high	97	1091	44	547	1.11	[0.79; 1.55]	17.6%	17.7%
ENGAGE_high	103	1107	54	540	0.93	[0.68; 1.27]	21.8%	21.0%
Study 302	54	673	31	448	1.16	[0.76; 1.77]	11.2%	11.4%
**Common effect model**		**5626**		**4261**	**1.08**	**[0.94; 1.25]**	**100.0%**	**.**
**Random effects model**					**1.08**	**[0.93; 1.24]**	**.**	**100.0%**
**Prediction interval**						**[0.89; 1.30]**		

Heterogeneity: $I^2 = 0\%$, $\tau^2 = 0$, $p = 0.96$

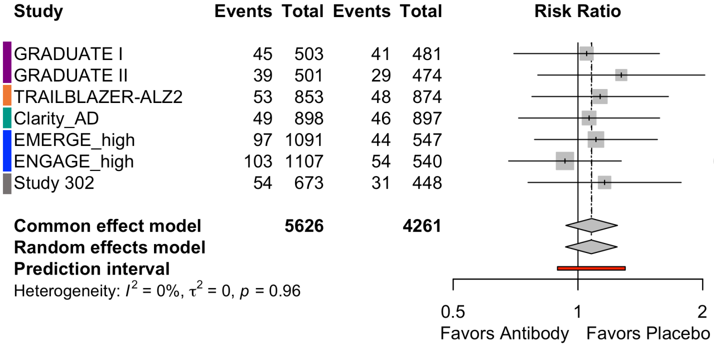

Gantenerumab **Donanemab** **Lecanemab** **Aducanumab** **Beprineuzumab**

S24 Figure: Sensitivity analysis 1 (excluding solanezumab). Forest plots for safety outcomes related to clinical symptoms.
