# Supplementary material for: Efficacy and safety of passive immunotherapies targeting amyloid beta in Alzheimer’s disease: A systematic review and meta-analysis
Source: PLoS Med. 2025 Mar 31;22(3):e1004568. doi: 10.1371/journal.pmed.1004568 (PMC12002640; doi:10.1371/journal.pmed.1004568)
Supplement: S25 Fig — (a) The Clinical Dementia Rating-Sum of Boxes (CDR-SB) and (b) Alzheimer’s Disease Assessment Scale-Cognitive Subscale (ADAS-Cog). (PDF) [file pmed.1004568.s026.pdf]

(a) CDR-SB

Study	MD	SE(MD)	MD	95%-CI	Weight (common)	Weight (random)
**Lecanemab**						
Clarity_AD	-0.4500	0.1122	-0.45	[-0.67; -0.23]	19.1%	14.8%
**Gantenerumab**						
GRADUATE I	-0.3100	0.1811	-0.31	[-0.66; 0.04]	7.4%	9.4%
GRADUATE II	-0.1900	0.1837	-0.19	[-0.55; 0.17]	7.1%	9.3%
**Common effect model**			**-0.25**	**[-0.50; 0.00]**	**14.5%**	.
**Random effects model**			**-0.25**	**[-0.50; 0.00]**	.	**18.7%**
Heterogeneity: $I^2 = 0\%$, $\tau^2 = 0$, $p = 0.64$						
**Donanemab**						
TRAILBLAZER-ALZ2	-0.7000	0.1403	-0.70	[-0.97; -0.43]	12.2%	12.3%
**Aducanumab**						
EMERGE	-0.3252	0.1340	-0.33	[-0.59; -0.06]	13.4%	12.9%
ENGAGE	-0.0742	0.1311	-0.07	[-0.33; 0.18]	14.0%	13.1%
**Common effect model**			**-0.20**	**[-0.38; -0.01]**	**27.5%**	.
**Random effects model**			**-0.20**	**[-0.44; 0.05]**	.	**25.9%**
Heterogeneity: $I^2 = 44\%$, $\tau^2 = 0.0139$, $p = 0.18$						
**Solanezumab**						
EXPEDITION3	-0.3400	0.1173	-0.34	[-0.57; -0.11]	17.5%	14.3%
EXPEDITION1	0.1000	0.2296	0.10	[-0.35; 0.55]	4.6%	7.0%
EXPEDITION2	-0.3000	0.2296	-0.30	[-0.75; 0.15]	4.6%	7.0%
**Common effect model**			**-0.26**	**[-0.44; -0.07]**	**26.7%**	.
**Random effects model**			**-0.23**	**[-0.48; 0.03]**	.	**28.2%**
Heterogeneity: $I^2 = 32\%$, $\tau^2 = 0.0177$, $p = 0.23$						
**Common effect model**			**-0.33**	**[-0.43; -0.23]**	**100.0%**	.
**Random effects model**			**-0.31**	**[-0.46; -0.17]**	.	**100.0%**
**Prediction interval**				**[-0.71; 0.08]**		

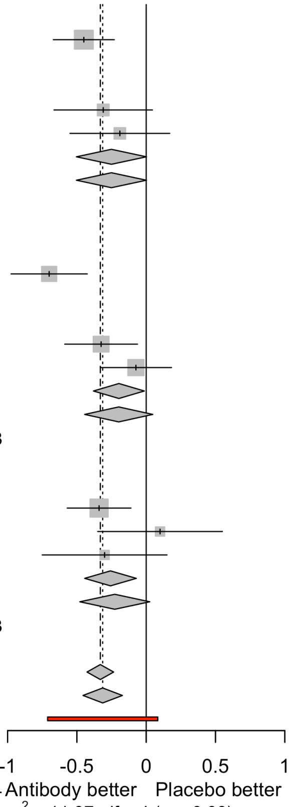

Heterogeneity: $I^2 = 50\%$, $\tau^2 = 0.0230$, $p = 0.04$

Test for subgroup differences (common effect): $\chi^2_4 = 11.07$, df = 4 ($p = 0.03$)

Test for subgroup differences (random effects): $\chi^2_4 = 9.96$, df = 4 ($p = 0.04$)

(b) ADAS-Cog

Study	SMD	SE(SMD)	SMD	95%-CI	Weight (common)	Weight (random)
**Lecanemab**						
Clarity_AD	-0.1686	0.0496	-0.17	[-0.27; -0.07]	11.1%	11.1%
**Gantenerumab**						
GRADUATE I	-0.1081	0.0560	-0.11	[-0.22; 0.00]	8.7%	8.7%
GRADUATE II	-0.1114	0.0504	-0.11	[-0.21; -0.01]	10.7%	10.7%
**Common effect model**			**-0.11**	**[-0.18; -0.04]**	**19.4%**	.
**Random effects model**			**-0.11**	**[-0.18; -0.04]**	.	**19.4%**
Heterogeneity: $I^2 = 0\%$, $\tau^2 = 0$, $p = 0.97$						
**Donanemab**						
TRAILBLAZER-ALZ2	-0.1360	0.0403	-0.14	[-0.22; -0.06]	16.7%	16.7%
**Aducanumab**						
EMERGE	-0.1936	0.0737	-0.19	[-0.34; -0.05]	5.0%	5.0%
ENGAGE	-0.1145	0.0744	-0.11	[-0.26; 0.03]	4.9%	4.9%
**Common effect model**			**-0.15**	**[-0.26; -0.05]**	**9.9%**	.
**Random effects model**			**-0.15**	**[-0.26; -0.05]**	.	**9.9%**
Heterogeneity: $I^2 = 0\%$, $\tau^2 = 0$, $p = 0.45$						
**Solanezumab**						
EXPEDITION3	-0.0681	0.0406	-0.07	[-0.15; 0.01]	16.5%	16.5%
EXPEDITION1	-0.0553	0.0459	-0.06	[-0.15; 0.03]	12.9%	12.9%
EXPEDITION2	-0.0821	0.0451	-0.08	[-0.17; 0.01]	13.4%	13.4%
**Common effect model**			**-0.07**	**[-0.12; -0.02]**	**42.8%**	.
**Random effects model**			**-0.07**	**[-0.12; -0.02]**	.	**42.8%**
Heterogeneity: $I^2 = 0\%$, $\tau^2 = 0$, $p = 0.92$						
**Common effect model**			**-0.11**	**[-0.14; -0.08]**	**100.0%**	.
**Random effects model**			**-0.11**	**[-0.14; -0.08]**	.	**100.0%**
**Prediction interval**				**[-0.15; -0.07]**		

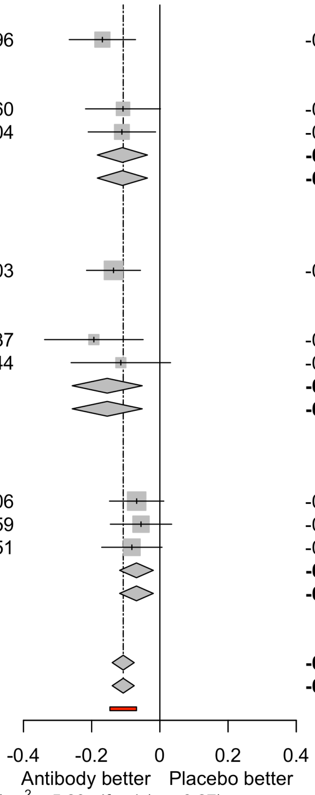

Heterogeneity: $I^2 = 0\%$, $\tau^2 = 0$, $p = 0.65$

Test for subgroup differences (common effect): $\chi^2_4 = 5.20$, df = 4 ($p = 0.27$)

Test for subgroup differences (random effects): $\chi^2_4 = 5.20$, df = 4 ($p = 0.27$)

S25 Figure: Sensitivity analysis 2 (excluding bapineuzumab). Forest plots for efficacy outcomes.
