# Supplementary material for: Efficacy and safety of passive immunotherapies targeting amyloid beta in Alzheimer’s disease: A systematic review and meta-analysis
Source: PLoS Med. 2025 Mar 31;22(3):e1004568. doi: 10.1371/journal.pmed.1004568 (PMC12002640; doi:10.1371/journal.pmed.1004568)
Supplement: S26 Fig — (a) Death, (b) Serious Adverse Event, (c) ARIA-E, (d) ARIA-H. ARIA-E: Amyloid-Related Imaging Abnormalities-Effusion, ARIA-H, Amyloid-Related Imaging Abnormalities-Hemorrhage. (PDF) [file pmed.1004568.s027.pdf]

(a) Death

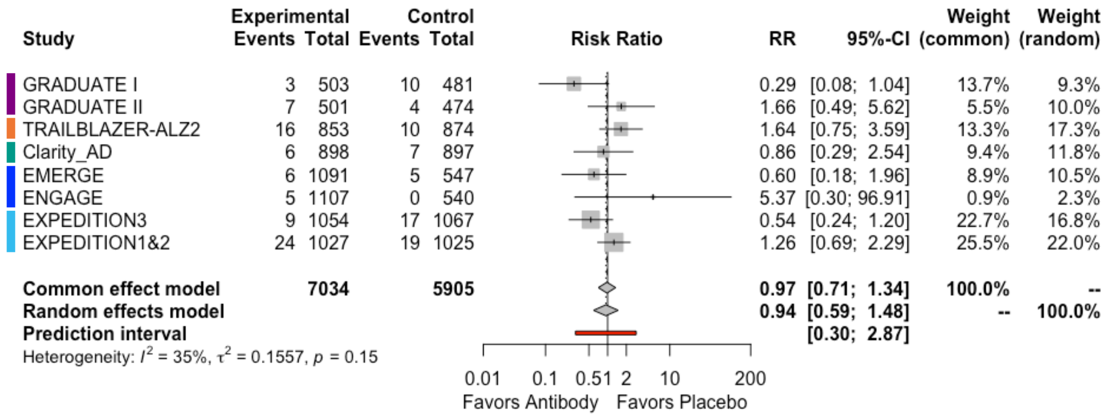

(b) Serious adverse events

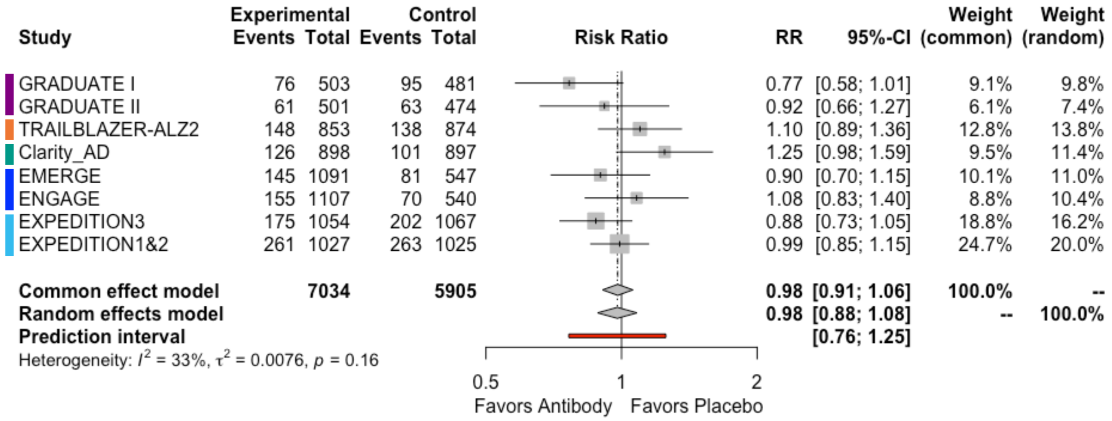

(c) ARIA-E

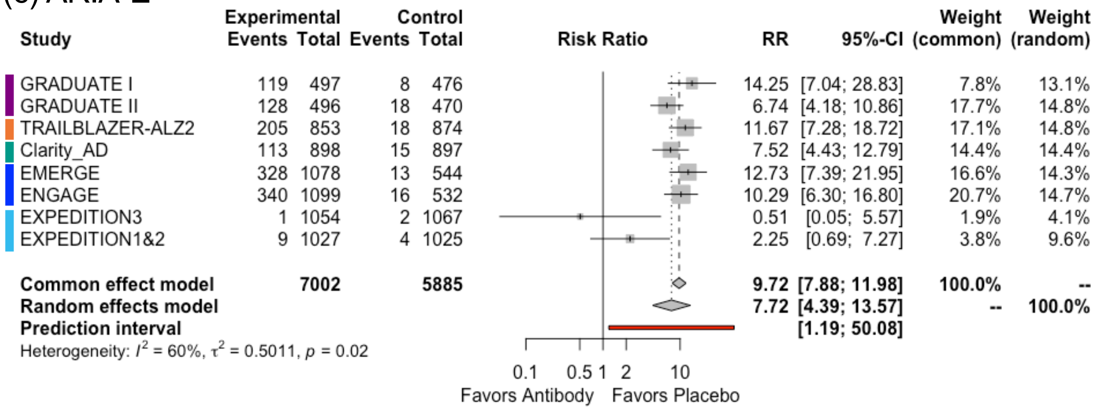

S26 Figure: Sensitivity analysis 2 (excluding bapineuzumab). Forest plots for safety outcomes.
