# Supplementary material for: Efficacy and safety of passive immunotherapies targeting amyloid beta in Alzheimer’s disease: A systematic review and meta-analysis
Source: PLoS Med. 2025 Mar 31;22(3):e1004568. doi: 10.1371/journal.pmed.1004568 (PMC12002640; doi:10.1371/journal.pmed.1004568)
Supplement: S27 Fig — (a) Headache, (b) Fall, and (c) Dizziness. (PDF) [file pmed.1004568.s028.pdf]

(a) Headache

Study	Experimental Events	Experimental Total	Control Events	Control Total	RR	95%-CI	Weight (common)	Weight (random)
GRADUATE I	60	503	43	481	1.33	[0.92; 1.93]	6.9%	7.3%
GRADUATE II	64	501	50	474	1.21	[0.86; 1.72]	8.0%	8.2%
TRAILBLAZER-ALZ2	119	853	86	874	1.42	[1.09; 1.84]	13.3%	14.3%
Clarity_AD	100	898	73	897	1.37	[1.03; 1.82]	11.4%	11.9%
EMERGE	217	1091	84	547	1.30	[1.03; 1.63]	17.5%	18.2%
ENGAGE	214	1107	81	540	1.29	[1.02; 1.63]	17.0%	17.6%
EXPEDITION3	87	1054	88	1067	1.00	[0.75; 1.33]	13.7%	12.2%
EXPEDITION1&2	72	1027	77	1025	0.93	[0.68; 1.27]	12.1%	10.3%
**Common effect model**		**7034**		**5905**	**1.23**	**[1.12; 1.36]**	**100.0%**	**--**
**Random effects model**					**1.23**	**[1.11; 1.36]**	**--**	**100.0%**
**Prediction interval**						**[1.06; 1.43]**		

Heterogeneity: $I^2 = 4\%$, $\tau^2 = 0.0009$, $p = 0.40$

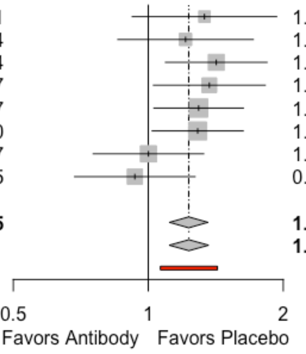

(b) Fall

Study	Experimental Events	Experimental Total	Control Events	Control Total	RR	95%-CI	Weight (common)	Weight (random)
GRADUATE I	63	503	62	481	0.97	[0.70; 1.35]	8.5%	8.6%
GRADUATE II	50	501	52	474	0.91	[0.63; 1.31]	7.1%	6.9%
TRAILBLAZER-ALZ2	114	853	110	874	1.06	[0.83; 1.36]	14.5%	14.8%
Clarity_AD	93	898	86	897	1.08	[0.82; 1.43]	11.5%	11.7%
EMERGE	144	1091	71	547	1.02	[0.78; 1.33]	12.6%	12.8%
ENGAGE	166	1107	57	540	1.42	[1.07; 1.88]	10.2%	11.3%
EXPEDITION3	162	1054	161	1067	1.02	[0.83; 1.25]	21.3%	21.0%
EXPEDITION1&2	92	1027	107	1025	0.86	[0.66; 1.12]	14.3%	12.8%
**Common effect model**		**7034**		**5905**	**1.04**	**[0.94; 1.14]**	**100.0%**	**--**
**Random effects model**					**1.04**	**[0.94; 1.14]**	**--**	**100.0%**
**Prediction interval**						**[0.89; 1.21]**		

Heterogeneity: $I^2 = 7\%$, $\tau^2 = 0.0016$, $p = 0.38$

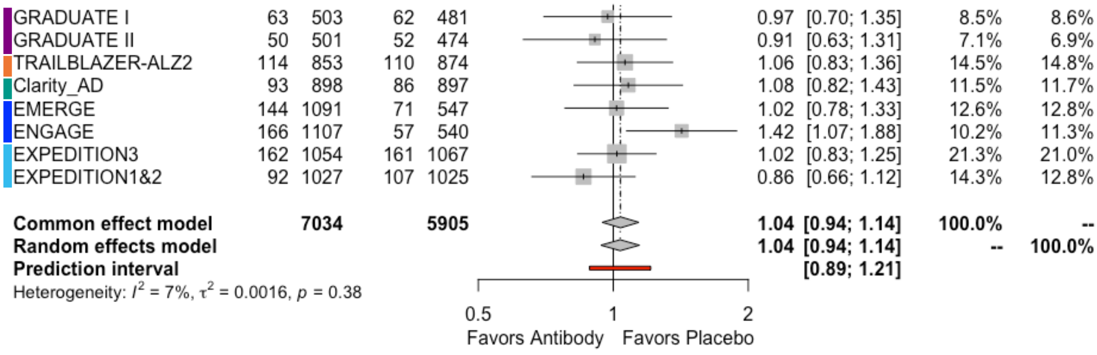

(c) Dizziness

Study	Experimental Events	Experimental Total	Control Events	Control Total	RR	95%-CI	Weight (common)	Weight (random)
GRADUATE I	45	503	41	481	1.05	[0.70; 1.57]	10.2%	10.3%
GRADUATE II	39	501	29	474	1.27	[0.80; 2.02]	7.3%	7.8%
TRAILBLAZER-ALZ2	53	853	48	874	1.13	[0.77; 1.65]	11.5%	11.8%
Clarity_AD	49	898	46	897	1.06	[0.72; 1.57]	11.2%	11.0%
EMERGE	97	1091	44	547	1.11	[0.79; 1.55]	14.3%	14.5%
ENGAGE	103	1107	54	540	0.93	[0.68; 1.27]	17.7%	17.2%
EXPEDITION3	62	1054	58	1067	1.08	[0.76; 1.53]	14.0%	14.0%
EXPEDITION1&2	58	1027	57	1025	1.02	[0.71; 1.45]	13.9%	13.4%
**Common effect model**		**7034**		**5905**	**1.06**	**[0.93; 1.21]**	**100.0%**	**--**
**Random effects model**					**1.06**	**[0.93; 1.21]**	**--**	**100.0%**
**Prediction interval**						**[0.90; 1.25]**		

Heterogeneity: $I^2 = 0\%$, $\tau^2 = 0$, $p = 0.98$

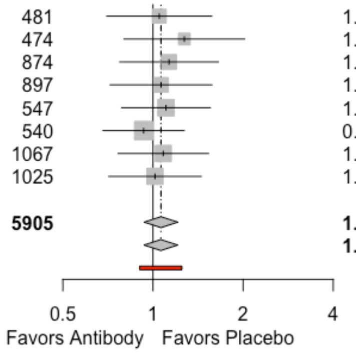

Gantenerumab **Donanemab** **Lecanemab** **Aducanumab** **Solanezumab**

S27 Figure: Sensitivity analysis 2 (excluding bapineuzumab). Forest plots for safety outcomes related to clinical symptoms.
