# Supplementary material for: Efficacy and safety of passive immunotherapies targeting amyloid beta in Alzheimer’s disease: A systematic review and meta-analysis
Source: PLoS Med. 2025 Mar 31;22(3):e1004568. doi: 10.1371/journal.pmed.1004568 (PMC12002640; doi:10.1371/journal.pmed.1004568)
Supplement: S28 Fig — (a) The Clinical Dementia Rating-Sum of Boxes (CDR-SB) and (b) Alzheimer’s Disease Assessment Scale-Cognitive Subscale (ADAS-Cog). (PDF) [file pmed.1004568.s029.pdf]

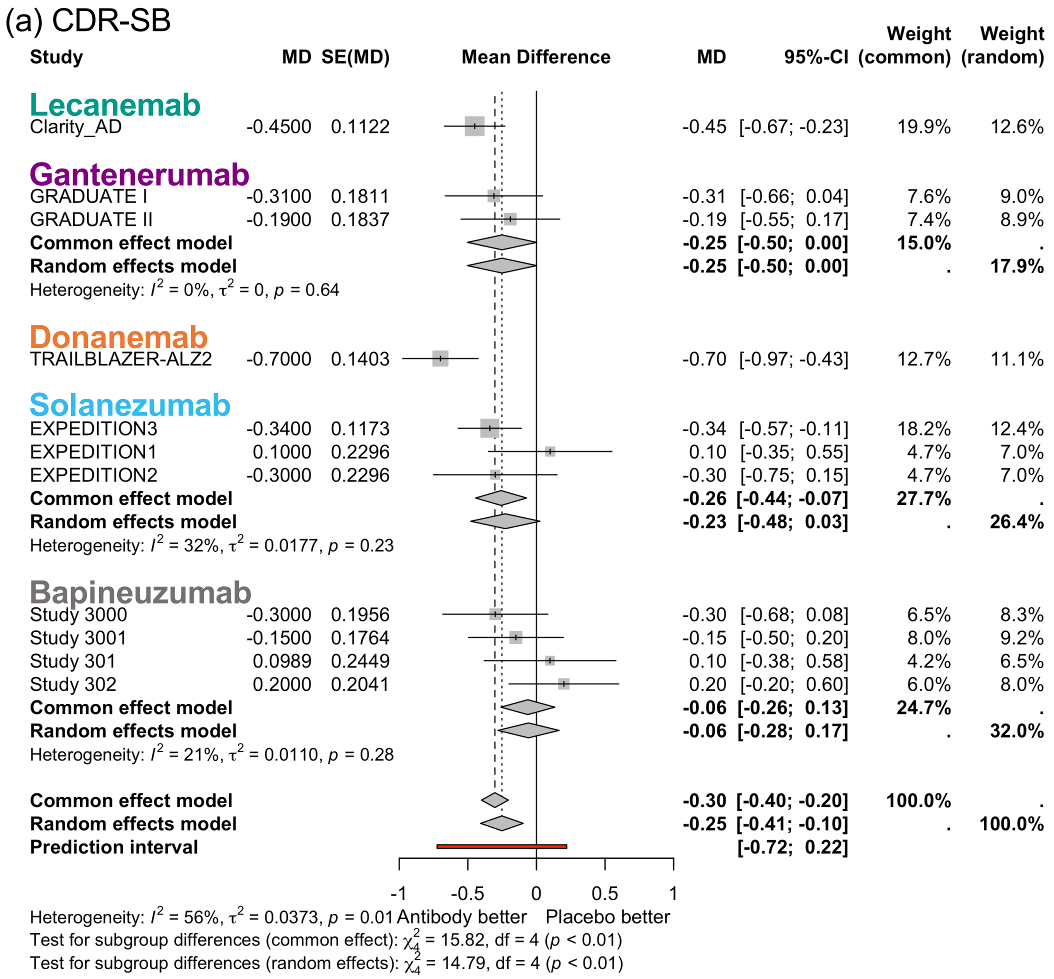


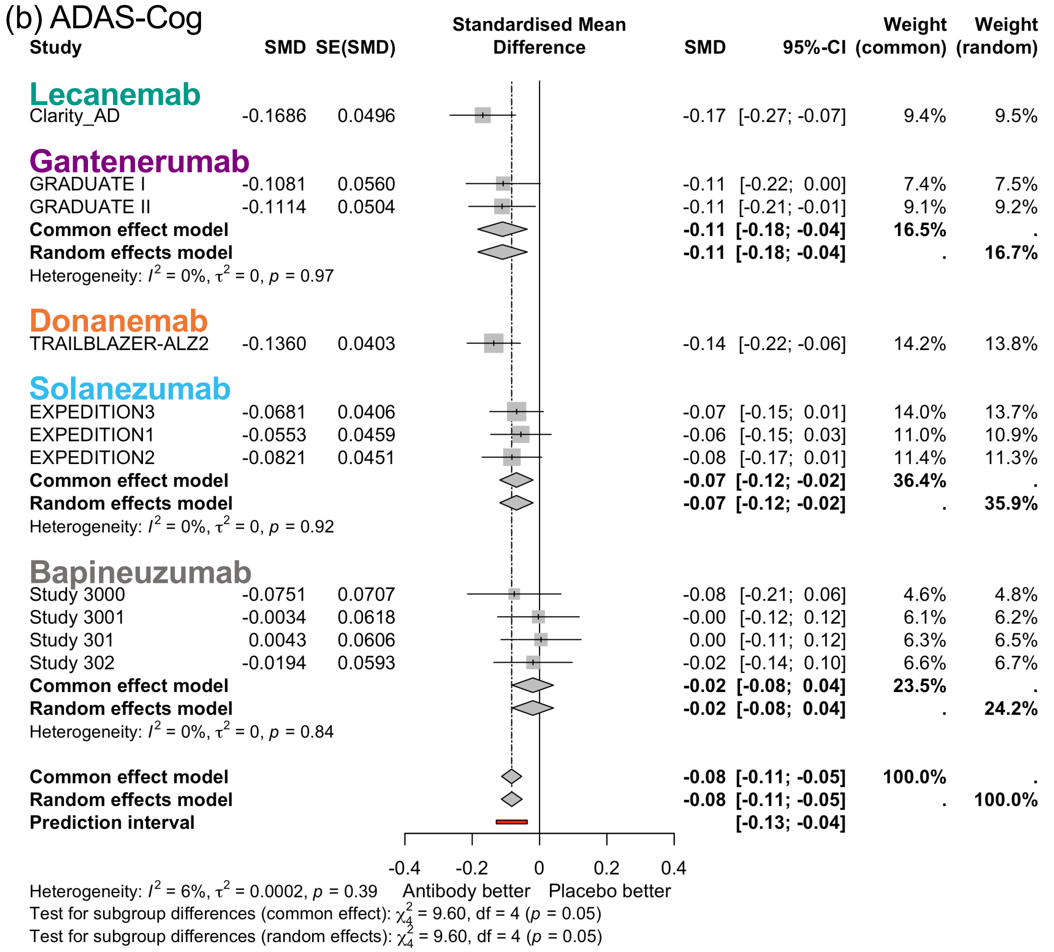


S28 Figure: Sensitivity analysis 3 (excluding aducanumab). Forest plots for efficacy outcomes.
