# Supplementary material for: Efficacy and safety of passive immunotherapies targeting amyloid beta in Alzheimer’s disease: A systematic review and meta-analysis
Source: PLoS Med. 2025 Mar 31;22(3):e1004568. doi: 10.1371/journal.pmed.1004568 (PMC12002640; doi:10.1371/journal.pmed.1004568)
Supplement: S29 Fig — (a) Death, (b) Serious Adverse Event, (c) ARIA-E, (d) ARIA-H. ARIA-E: Amyloid-Related Imaging Abnormalities-Effusion, ARIA-H: Amyloid-Related Imaging Abnormalities-Hemorrhage. (PDF) [file pmed.1004568.s030.pdf]

(a) Death

Study	Experimental Events	Experimental Total	Control Events	Control Total	RR	95%-CI	Weight (common)	Weight (random)
GRADUATE I	3	503	10	481	0.29	[0.08; 1.04]	11.4%	6.7%
GRADUATE II	7	501	4	474	1.66	[0.49; 5.62]	4.6%	7.3%
TRAILBLAZER-ALZ2	16	853	10	874	1.64	[0.75; 3.59]	11.1%	14.0%
Clarity_AD	6	898	7	897	0.86	[0.29; 2.54]	7.8%	8.8%
EXPEDITION3	9	1054	17	1067	0.54	[0.24; 1.20]	18.9%	13.6%
EXPEDITION1 & 2	24	1027	19	1025	1.26	[0.69; 2.29]	21.3%	19.3%
Study 3000	2	530	3	344	0.43	[0.07; 2.58]	4.1%	3.8%
Study 3001	4	654	4	439	0.67	[0.17; 2.67]	5.4%	5.9%
Study 301	11	666	7	524	1.24	[0.48; 3.17]	8.8%	10.9%
Study 302	15	673	5	448	2.00	[0.73; 5.46]	6.7%	9.9%
**Common effect model**		**7359**		**6573**	**1.02**	**[0.77; 1.37]**	**100.0%**	**.**
**Random effects model**					**1.01**	**[0.70; 1.45]**	**.**	**100.0%**
**Prediction interval**						**[0.45; 2.24]**		

Heterogeneity: $I^2 = 24\%$, $\tau^2 = 0.0860$, $p = 0.22$

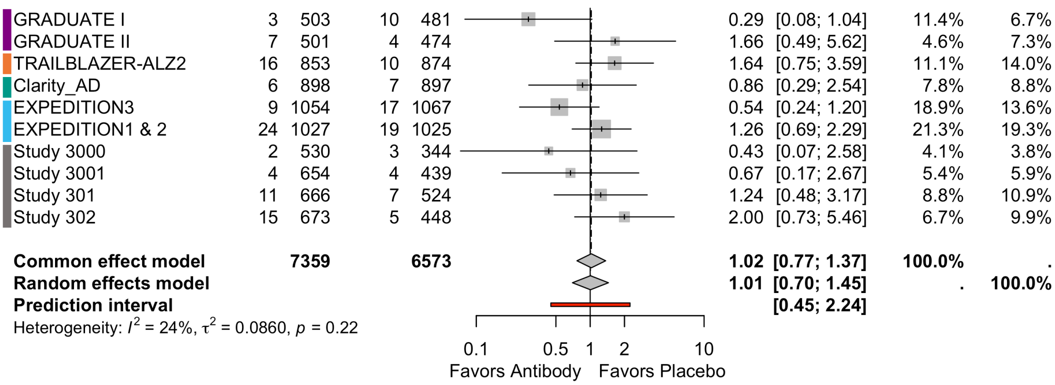

(b) Serious adverse events

Study	Experimental Events	Experimental Total	Control Events	Control Total	RR	95%-CI	Weight (common)	Weight (random)
GRADUATE I	76	503	95	481	0.77	[0.58; 1.01]	8.9%	10.4%
GRADUATE II	61	501	63	474	0.92	[0.66; 1.27]	5.9%	8.4%
TRAILBLAZER-ALZ2	148	853	138	874	1.10	[0.89; 1.36]	12.5%	13.2%
Clarity_AD	126	898	101	897	1.25	[0.98; 1.59]	9.3%	11.6%
EXPEDITION3	175	1054	202	1067	0.88	[0.73; 1.05]	18.4%	14.6%
EXPEDITION1 & 2	261	1027	263	1025	0.99	[0.85; 1.15]	24.1%	16.6%
Study 301	148	666	108	524	1.08	[0.87; 1.34]	11.1%	12.8%
Study 302	176	673	89	448	1.32	[1.05; 1.65]	9.8%	12.5%
**Common effect model**		**6175**		**5790**	**1.02**	**[0.95; 1.10]**	**100.0%**	**.**
**Random effects model**					**1.03**	**[0.91; 1.16]**	**.**	**100.0%**
**Prediction interval**						**[0.72; 1.47]**		

Heterogeneity: $I^2 = 55\%$, $\tau^2 = 0.0175$, $p = 0.03$

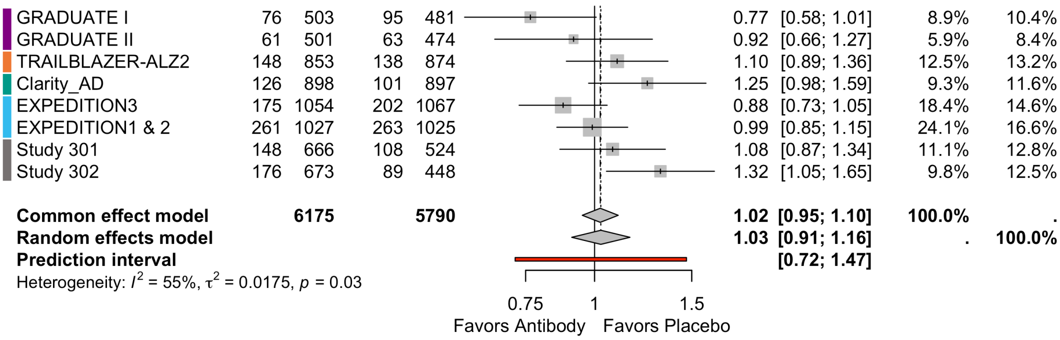

(c) ARIA-E

Study	Experimental Events	Experimental Total	Control Events	Control Total	RR	95%-CI	Weight (common)	Weight (random)
GRADUATE I	119	497	8	476	14.25	[7.04; 28.83]	10.1%	11.7%
GRADUATE II	128	496	18	470	6.74	[4.18; 10.86]	22.9%	12.4%
TRAILBLAZER-ALZ2	205	853	18	874	11.67	[7.28; 18.72]	22.1%	12.4%
Clarity_AD	113	898	15	897	7.52	[4.43; 12.79]	18.6%	12.2%
EXPEDITION3	1	1054	2	1067	0.51	[0.05; 5.57]	2.5%	5.9%
EXPEDITION1 & 2	9	1027	4	1025	2.25	[0.69; 7.27]	5.0%	10.1%
Study 3000	44	530	2	344	14.28	[3.48; 58.52]	3.0%	9.2%
Study 3001	109	654	9	439	8.13	[4.16; 15.87]	13.4%	11.8%
Study 301	45	666	1	524	35.41	[4.90; 256.00]	1.4%	7.1%
Study 302	103	448	1	673	154.73	[21.67; 1104.90]	1.0%	7.2%
**Common effect model**		**7123**		**6789**	**10.64**	**[8.49; 13.33]**	**100.0%**	**.**
**Random effects model**					**9.47**	**[4.30; 20.89]**	**.**	**100.0%**
**Prediction interval**						**[0.61; 147.89]**		

Heterogeneity: $I^2 = 65\%$, $\tau^2 = 1.2573$, $p < 0.01$

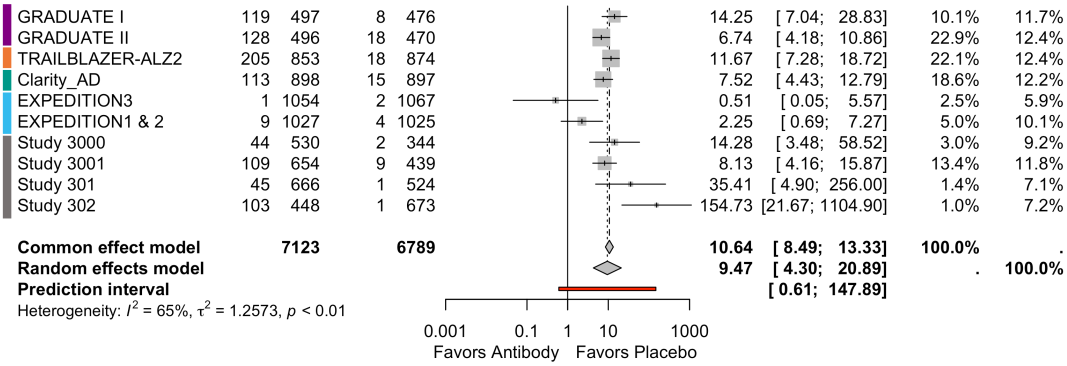

(d) ARIA-H

Study	Experimental Events	Experimental Total	Control Events	Control Total	RR	95%-CI	Weight (common)	Weight (random)
GRADUATE I	118	497	59	476	1.92	[1.44; 2.55]	16.1%	19.9%
GRADUATE II	109	496	57	470	1.81	[1.35; 2.43]	15.6%	19.7%
TRAILBLAZER-ALZ2	268	853	119	874	2.31	[1.90; 2.80]	31.4%	21.8%
Clarity_AD	155	898	81	897	1.91	[1.49; 2.46]	21.6%	20.6%
EXPEDITION1 & 2	50	1027	57	1025	0.88	[0.60; 1.27]	15.2%	18.0%
**Common effect model**		**3771**		**3742**	**1.86**	**[1.66; 2.09]**	**100.0%**	**.**
**Random effects model**					**1.71**	**[1.25; 2.36]**	**.**	**100.0%**
**Prediction interval**						**[0.52; 5.59]**		

Heterogeneity: $I^2 = 81\%$, $\tau^2 = 0.1117$, $p < 0.01$

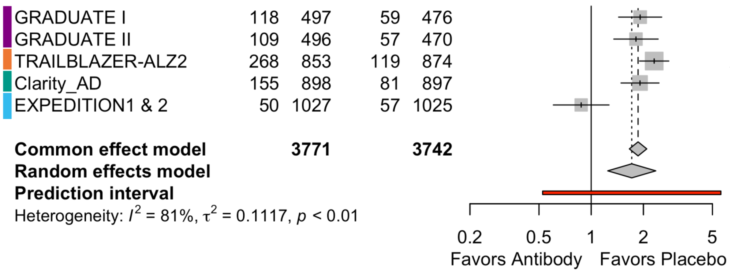

Gantenerumab **Donanemab** **Lecanemab** **Solanezumab** **Beprineuzumab**

S29 Figure: Sensitivity analysis 3 (excluding aducanumab). Forest plots for safety outcomes.
