# Supplementary material for: Efficacy and safety of passive immunotherapies targeting amyloid beta in Alzheimer’s disease: A systematic review and meta-analysis
Source: PLoS Med. 2025 Mar 31;22(3):e1004568. doi: 10.1371/journal.pmed.1004568 (PMC12002640; doi:10.1371/journal.pmed.1004568)
Supplement: S30 Fig — (a) Headache, (b) Fall, and (c) Dizziness. (PDF) [file pmed.1004568.s031.pdf]

## (a) Headache

| Study | Experimental Events | Experimental Total | Control Events | Control Total | RR | 95%-CI | Weight (common) | Weight (random) |
|---|---|---|---|---|---|---|---|---|
| GRADUATE I | 60 | 503 | 43 | 481 | 1.33 | [0.92; 1.93] | 8.3% | 9.1% |
| GRADUATE II | 64 | 501 | 50 | 474 | 1.21 | [0.86; 1.72] | 9.7% | 10.3% |
| TRAILBLAZER-ALZ2 | 119 | 853 | 86 | 874 | 1.42 | [1.09; 1.84] | 16.0% | 17.4% |
| Clarity_AD | 100 | 898 | 73 | 897 | 1.37 | [1.03; 1.82] | 13.8% | 14.7% |
| EXPEDITION3 | 87 | 1054 | 88 | 1067 | 1.00 | [0.75; 1.33] | 16.5% | 15.0% |
| EXPEDITION1 & 2 | 72 | 1027 | 77 | 1025 | 0.93 | [0.68; 1.27] | 14.5% | 12.8% |
| Study 301 | 64 | 666 | 49 | 524 | 1.03 | [0.72; 1.46] | 10.3% | 10.0% |
| Study 302 | 78 | 673 | 48 | 448 | 1.08 | [0.77; 1.52] | 10.9% | 10.8% |
| **Common effect model** | | **6175** | | **5790** | **1.17** | **[1.05; 1.30]** | **100.0%** | **.** |
| **Random effects model** | | | | | **1.17** | **[1.04; 1.31]** | **.** | **100.0%** |
| **Prediction interval** | | | | | | **[0.97; 1.40]** | | |

Heterogeneity: $I^2 = 9\%$, $\tau^2 = 0.0022$, $p = 0.36$

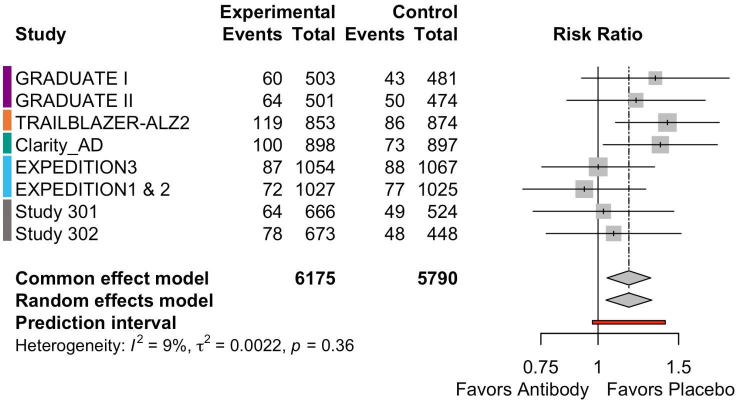


## (b) Fall

| Study | Experimental Events | Experimental Total | Control Events | Control Total | RR | 95%-CI | Weight (common) | Weight (random) |
|---|---|---|---|---|---|---|---|---|
| GRADUATE I | 63 | 503 | 62 | 481 | 0.97 | [0.70; 1.35] | 8.6% | 8.5% |
| GRADUATE II | 50 | 501 | 52 | 474 | 0.91 | [0.63; 1.31] | 7.2% | 6.8% |
| TRAILBLAZER-ALZ2 | 114 | 853 | 110 | 874 | 1.06 | [0.83; 1.36] | 14.7% | 15.3% |
| Clarity_AD | 93 | 898 | 86 | 897 | 1.08 | [0.82; 1.43] | 11.7% | 11.8% |
| EXPEDITION3 | 162 | 1054 | 161 | 1067 | 1.02 | [0.83; 1.25] | 21.7% | 22.7% |
| EXPEDITION1 & 2 | 92 | 1027 | 107 | 1025 | 0.86 | [0.66; 1.12] | 14.5% | 13.1% |
| Study 301 | 86 | 666 | 73 | 524 | 0.93 | [0.69; 1.24] | 11.1% | 10.9% |
| Study 302 | 100 | 673 | 64 | 448 | 1.04 | [0.78; 1.39] | 10.4% | 10.9% |
| **Common effect model** | | **6175** | | **5790** | **0.99** | **[0.90; 1.09]** | **100.0%** | **.** |
| **Random effects model** | | | | | **0.99** | **[0.90; 1.09]** | **.** | **100.0%** |
| **Prediction interval** | | | | | | **[0.88; 1.12]** | | |

Heterogeneity: $I^2 = 0\%$, $\tau^2 = 0$, $p = 0.93$

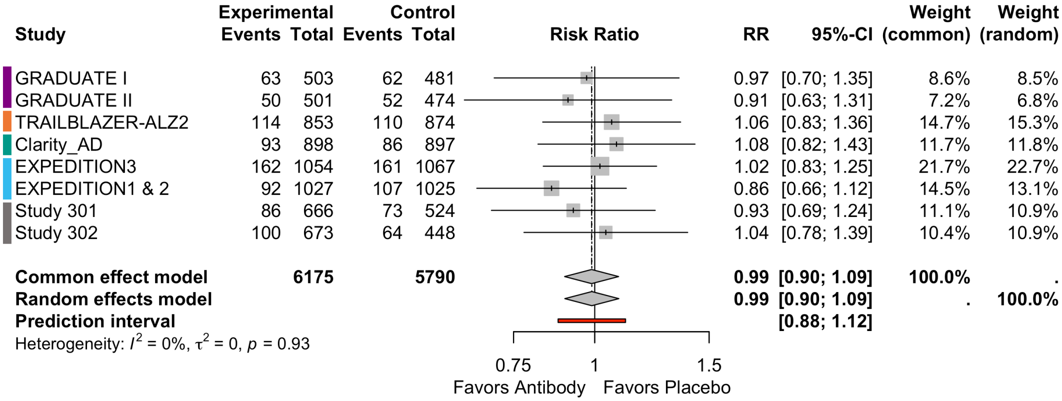


## (c) Dizziness

| Study | Experimental Events | Experimental Total | Control Events | Control Total | RR | 95%-CI | Weight (common) | Weight (random) |
|---|---|---|---|---|---|---|---|---|
| GRADUATE I | 45 | 503 | 41 | 481 | 1.05 | [0.70; 1.57] | 13.2% | 13.3% |
| GRADUATE II | 39 | 501 | 29 | 474 | 1.27 | [0.80; 2.02] | 9.4% | 10.1% |
| TRAILBLAZER-ALZ2 | 53 | 853 | 48 | 874 | 1.13 | [0.77; 1.65] | 15.0% | 15.1% |
| Clarity_AD | 49 | 898 | 46 | 897 | 1.06 | [0.72; 1.57] | 14.5% | 14.2% |
| EXPEDITION3 | 62 | 1054 | 58 | 1067 | 1.08 | [0.76; 1.53] | 18.2% | 18.0% |
| EXPEDITION1 & 2 | 58 | 1027 | 57 | 1025 | 1.02 | [0.71; 1.45] | 18.0% | 17.2% |
| Study 302 | 54 | 673 | 31 | 448 | 1.16 | [0.76; 1.77] | 11.7% | 12.0% |
| **Common effect model** | | **5509** | | **5266** | **1.10** | **[0.95; 1.27]** | **100.0%** | **.** |
| **Random effects model** | | | | | **1.10** | **[0.95; 1.27]** | **.** | **100.0%** |
| **Prediction interval** | | | | | | **[0.90; 1.33]** | | |

Heterogeneity: $I^2 = 0\%$, $\tau^2 = 0$, $p = 0.99$

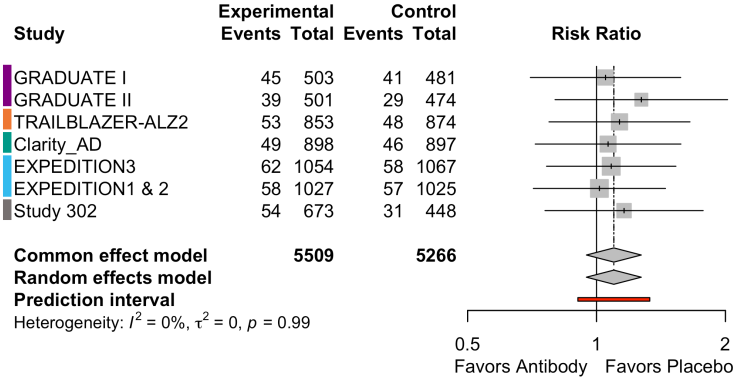


**Gantenerumab** **Donanemab** **Lecanemab** **Solanezumab** **Beprineuzumab**

S30 Figure: Sensitivity analysis 3 (excluding aducanumab). Forest plots for safety outcomes related to clinical symptoms.
