# Supplementary material for: Efficacy and safety of passive immunotherapies targeting amyloid beta in Alzheimer’s disease: A systematic review and meta-analysis
Source: PLoS Med. 2025 Mar 31;22(3):e1004568. doi: 10.1371/journal.pmed.1004568 (PMC12002640; doi:10.1371/journal.pmed.1004568)
Supplement: S32 Fig — (a) Death, (b) Serious Adverse Event, (c) ARIA-E, (d) ARIA-H. ARIA-E, Amyloid-Related Imaging Abnormalities-Effusion; ARIA-H, Amyloid-Related Imaging Abnormalities-Hemorrhage. (PDF) [file pmed.1004568.s033.pdf]

(a) Death

Study	Experimental Events	Experimental Total	Control Events	Control Total	RR	95%-CI	Weight (common)	Weight (random)
GRADUATE I	3	503	10	481	0.29	[0.08; 1.04]	26.5%	15.7%
GRADUATE II	7	501	4	474	1.66	[0.49; 5.62]	10.7%	16.8%
TRAILBLAZER-ALZ2	16	853	10	874	1.64	[0.75; 3.59]	25.6%	26.4%
Clarity_AD	6	898	7	897	0.86	[0.29; 2.54]	18.2%	19.3%
EMERGE	6	1091	5	547	0.60	[0.18; 1.96]	17.3%	17.5%
ENGAGE	5	1107	0	540	5.37	[0.30; 96.91]	1.7%	4.3%
**Common effect model**		**4953**		**3813**	**1.03**	**[0.66; 1.59]**	**100.0%**	**--**
**Random effects model**					**0.97**	**[0.52; 1.84]**	**--**	**100.0%**
**Prediction interval**						**[0.19; 4.93]**		

Heterogeneity: $I^2 = 37\%$, $\tau^2 = 0.2367$, $p = 0.16$

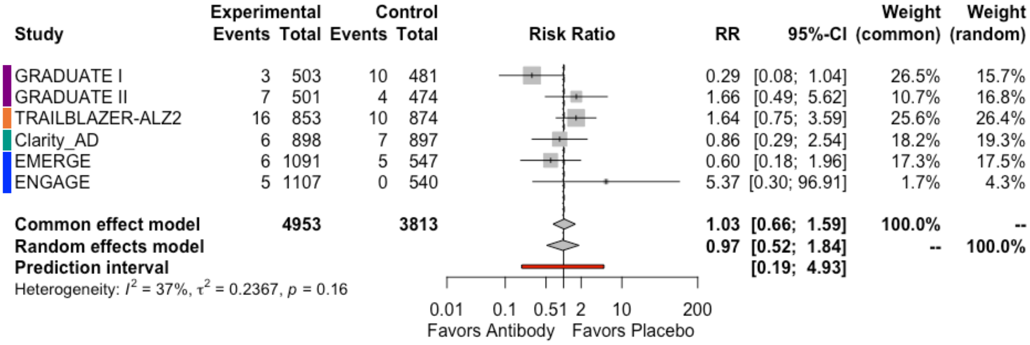

(b) Serious adverse events

Study	Experimental Events	Experimental Total	Control Events	Control Total	RR	95%-CI	Weight (common)	Weight (random)
GRADUATE I	76	503	95	481	0.77	[0.58; 1.01]	16.2%	15.6%
GRADUATE II	61	501	63	474	0.92	[0.66; 1.27]	10.8%	12.4%
TRAILBLAZER-ALZ2	148	853	138	874	1.10	[0.89; 1.36]	22.7%	20.6%
Clarity_AD	126	898	101	897	1.25	[0.98; 1.59]	16.8%	17.8%
EMERGE	145	1091	81	547	0.90	[0.70; 1.15]	17.9%	17.2%
ENGAGE	155	1107	70	540	1.08	[0.83; 1.40]	15.7%	16.4%
**Common effect model**		**4953**		**3813**	**1.01**	**[0.91; 1.12]**	**100.0%**	**--**
**Random effects model**					**1.00**	**[0.87; 1.15]**	**--**	**100.0%**
**Prediction interval**						**[0.69; 1.46]**		

Heterogeneity: $I^2 = 43\%$, $\tau^2 = 0.0132$, $p = 0.12$

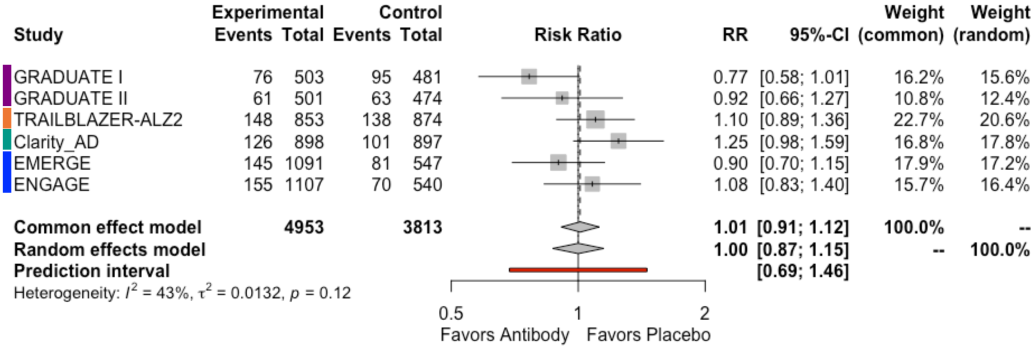

(c) ARIA-E

Study	Experimental Events	Experimental Total	Control Events	Control Total	RR	95%-CI	Weight (common)	Weight (random)
GRADUATE I	119	497	8	476	14.25	[7.04; 28.83]	8.3%	9.9%
GRADUATE II	128	496	18	470	6.74	[4.18; 10.86]	18.8%	19.6%
TRAILBLAZER-ALZ2	205	853	18	874	11.67	[7.28; 18.72]	18.1%	19.9%
Clarity_AD	113	898	15	897	7.52	[4.43; 12.79]	15.3%	16.4%
EMERGE	328	1078	13	544	12.73	[7.39; 21.95]	17.6%	15.6%
ENGAGE	340	1099	16	532	10.29	[6.30; 16.80]	21.9%	18.7%
**Common effect model**		**4921**		**3793**	**10.21**	**[8.23; 12.66]**	**100.0%**	**--**
**Random effects model**					**9.85**	**[7.81; 12.42]**	**--**	**100.0%**
**Prediction interval**						**[6.29; 15.43]**		

Heterogeneity: $I^2 = 15\%$, $\tau^2 = 0.0122$, $p = 0.32$

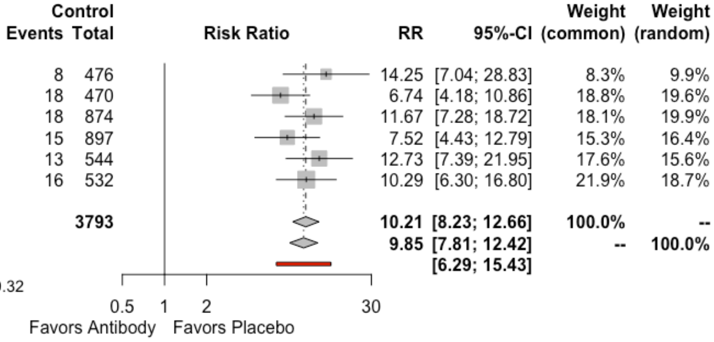

Gantenerumab **Donanemab** **Lecanemab** **Aducanumab**

S32 Figure: Sensitivity analysis 4 (excluding solanezumab and bapineuzumab). Forest plots for safety outcomes.
