# Supplementary material for: Efficacy and safety of passive immunotherapies targeting amyloid beta in Alzheimer’s disease: A systematic review and meta-analysis
Source: PLoS Med. 2025 Mar 31;22(3):e1004568. doi: 10.1371/journal.pmed.1004568 (PMC12002640; doi:10.1371/journal.pmed.1004568)
Supplement: S33 Fig — (a) Headache, (b) Fall, and (c) Dizziness. (PDF) [file pmed.1004568.s034.pdf]

(a) Headache

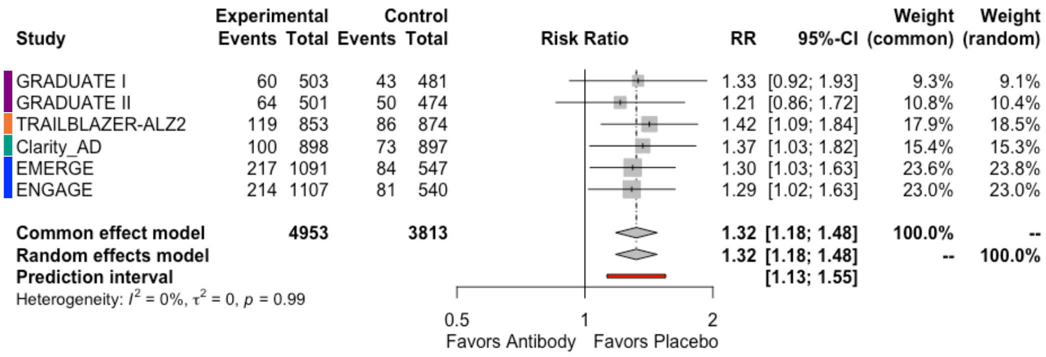


(b) Fall

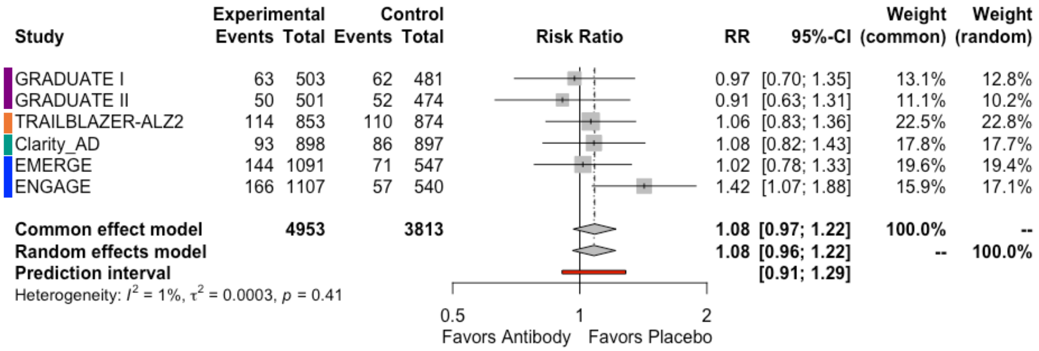


(c) Dizziness

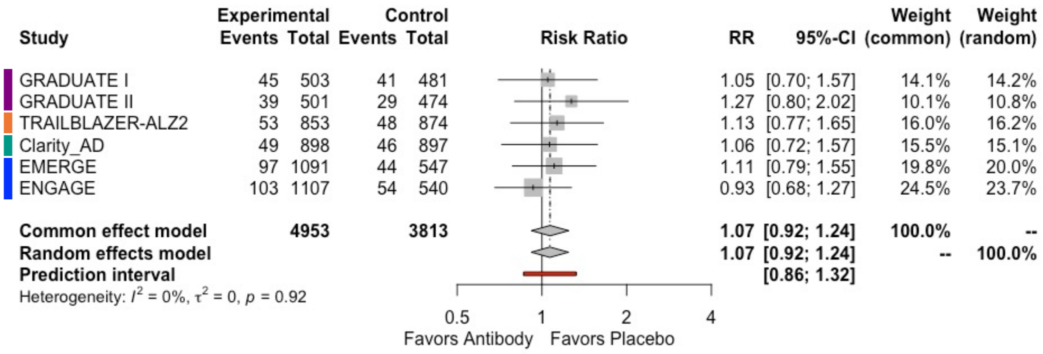


**Gantenerumab** **Donanemab** **Lecanemab** **Aducanumab**

S33 Figure: Sensitivity analysis 4 (excluding solanezumab and bapineuzumab). Forest plots for safety outcomes related to clinical symptoms.
