# Supplementary material for: Efficacy and safety of passive immunotherapies targeting amyloid beta in Alzheimer’s disease: A systematic review and meta-analysis
Source: PLoS Med. 2025 Mar 31;22(3):e1004568. doi: 10.1371/journal.pmed.1004568 (PMC12002640; doi:10.1371/journal.pmed.1004568)
Supplement: S35 Fig — (a) Death, (b)Serious Adverse Event, (c) ARIA-E, (d) ARIA-H. ARIA-E, Amyloid-Related Imaging Abnormalities-Effusion, ARIA-H, Amyloid-Related Imaging Abnormalities-Hemorrhage. (PDF) [file pmed.1004568.s036.pdf]

(a) Death

Study	Experimental Events	Experimental Total	Control Events	Control Total	RR	95%-CI	Weight (common)	Weight (random)
GRADUATE I	3	503	10	481	0.29	[0.08; 1.04]	32.8%	20.8%
GRADUATE II	7	501	4	474	1.66	[0.49; 5.62]	13.2%	21.9%
TRAILBLAZER-ALZ2	16	853	10	874	1.64	[0.75; 3.59]	31.6%	32.5%
Clarity_AD	6	898	7	897	0.86	[0.29; 2.54]	22.4%	24.8%
**Common effect model**		**2755**		**2726**	**1.02**	**[0.63; 1.67]**	**100.0%**	**.**
**Random effects model**					**0.97**	**[0.45; 2.11]**	**.**	**100.0%**
**Prediction interval**						**[0.05; 18.64]**		

Heterogeneity: $I^2 = 48\%$, $\tau^2 = 0.3160$, $p = 0.12$

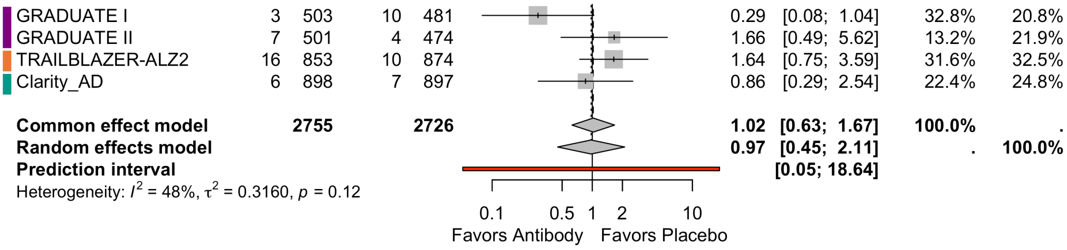

(b) Serious adverse events

Study	Experimental Events	Experimental Total	Control Events	Control Total	RR	95%-CI	Weight (common)	Weight (random)
GRADUATE I	76	503	95	481	0.77	[0.58; 1.01]	24.3%	24.1%
GRADUATE II	61	501	63	474	0.92	[0.66; 1.27]	16.2%	20.4%
TRAILBLAZER-ALZ2	148	853	138	874	1.10	[0.89; 1.36]	34.1%	29.0%
Clarity_AD	126	898	101	897	1.25	[0.98; 1.59]	25.3%	26.4%
**Common effect model**		**2755**		**2726**	**1.03**	**[0.90; 1.16]**	**100.0%**	**.**
**Random effects model**					**1.00**	**[0.81; 1.24]**	**.**	**100.0%**
**Prediction interval**						**[0.43; 2.35]**		

Heterogeneity: $I^2 = 61\%$, $\tau^2 = 0.0276$, $p = 0.05$

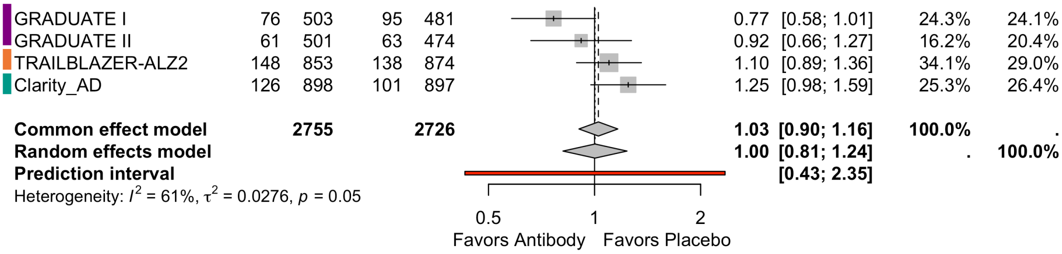

(c) ARIA-E

Study	Experimental Events	Experimental Total	Control Events	Control Total	RR	95%-CI	Weight (common)	Weight (random)
GRADUATE I	119	497	8	476	14.25	[7.04; 28.83]	13.7%	17.0%
GRADUATE II	128	496	18	470	6.74	[4.18; 10.86]	31.1%	28.7%
TRAILBLAZER-ALZ2	205	853	18	874	11.67	[7.28; 18.72]	29.9%	29.1%
Clarity_AD	113	898	15	897	7.52	[4.43; 12.79]	25.2%	25.2%
**Common effect model**		**2744**		**2717**	**9.44**	**[7.26; 12.28]**	**100.0%**	**.**
**Random effects model**					**9.23**	**[6.61; 12.88]**	**.**	**100.0%**
**Prediction interval**						**[2.94; 28.93]**		

Heterogeneity: $I^2 = 35\%$, $\tau^2 = 0.0416$, $p = 0.20$

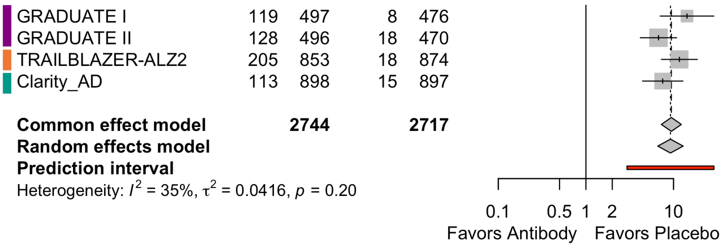

(d) ARIA-H

Study	Experimental Events	Experimental Total	Control Events	Control Total	RR	95%-CI	Weight (common)	Weight (random)
GRADUATE I	118	497	59	476	1.92	[1.44; 2.55]	19.0%	18.5%
GRADUATE II	109	496	57	470	1.81	[1.35; 2.43]	18.4%	17.5%
TRAILBLAZER-ALZ2	268	853	119	874	2.31	[1.90; 2.80]	37.0%	40.2%
Clarity_AD	155	898	81	897	1.91	[1.49; 2.46]	25.5%	23.8%
**Common effect model**		**2744**		**2717**	**2.04**	**[1.80; 2.31]**	**100.0%**	**.**
**Random effects model**					**2.04**	**[1.81; 2.31]**	**.**	**100.0%**
**Prediction interval**						**[1.56; 2.68]**		

Heterogeneity: $I^2 = 0\%$, $\tau^2 = 0$, $p = 0.46$

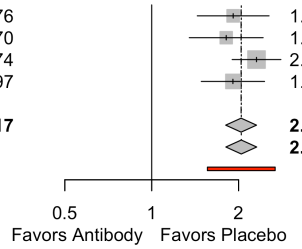

Gantenerumab **Donanemab** **Lecanemab**

S35 Figure: Sensitivity analysis 5 (excluding solanezumab, bapineuzumab, and aducanumab). Forest plots for safety outcomes.
