# Supplementary material for: Efficacy and safety of passive immunotherapies targeting amyloid beta in Alzheimer’s disease: A systematic review and meta-analysis
Source: PLoS Med. 2025 Mar 31;22(3):e1004568. doi: 10.1371/journal.pmed.1004568 (PMC12002640; doi:10.1371/journal.pmed.1004568)
Supplement: S36 Fig — (a) Headache, (b) Fall, and (c) Dizziness. (PDF) [file pmed.1004568.s037.pdf]

(a) Headache

Study	Experimental Events	Experimental Total	Control Events	Control Total	RR	95%-CI	Weight (common)	Weight (random)
GRADUATE I	60	503	43	481	1.33	[0.92; 1.93]	17.4%	17.1%
GRADUATE II	64	501	50	474	1.21	[0.86; 1.72]	20.3%	19.5%
TRAILBLAZER-ALZ2	119	853	86	874	1.42	[1.09; 1.84]	33.5%	34.7%
Clarity_AD	100	898	73	897	1.37	[1.03; 1.82]	28.8%	28.6%
**Common effect model**		**2755**		**2726**	**1.35**	**[1.16; 1.57]**	**100.0%**	**.**
**Random effects model**					**1.35**	**[1.15; 1.57]**	**.**	**100.0%**
**Prediction interval**						**[0.96; 1.89]**		

Heterogeneity: $I^2 = 0\%$, $\tau^2 = 0$, $p = 0.91$

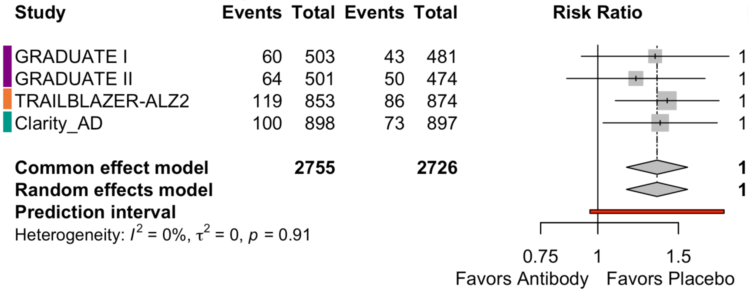

(b) Fall

Study	Experimental Events	Experimental Total	Control Events	Control Total	RR	95%-CI	Weight (common)	Weight (random)
GRADUATE I	63	503	62	481	0.97	[0.70; 1.35]	20.3%	20.1%
GRADUATE II	50	501	52	474	0.91	[0.63; 1.31]	17.2%	16.0%
TRAILBLAZER-ALZ2	114	853	110	874	1.06	[0.83; 1.36]	34.9%	36.1%
Clarity_AD	93	898	86	897	1.08	[0.82; 1.43]	27.6%	27.8%
**Common effect model**		**2755**		**2726**	**1.02**	**[0.88; 1.18]**	**100.0%**	**.**
**Random effects model**					**1.02**	**[0.88; 1.18]**	**.**	**100.0%**
**Prediction interval**						**[0.74; 1.41]**		

Heterogeneity: $I^2 = 0\%$, $\tau^2 = 0$, $p = 0.87$

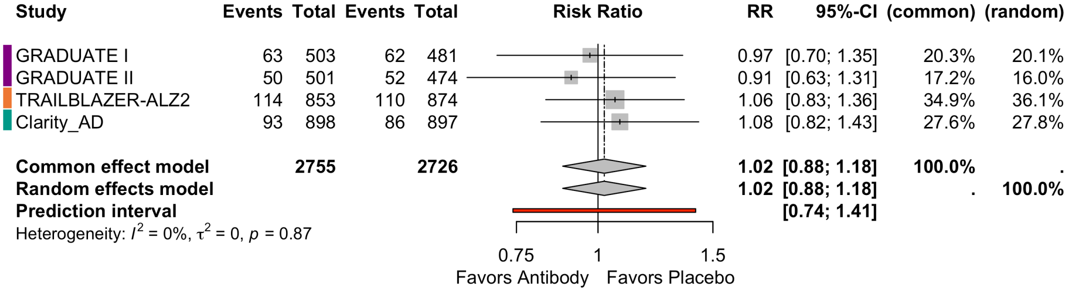

(c) Dizziness

Study	Experimental Events	Experimental Total	Control Events	Control Total	RR	95%-CI	Weight (common)	Weight (random)
GRADUATE I	45	503	41	481	1.05	[0.70; 1.57]	25.4%	25.2%
GRADUATE II	39	501	29	474	1.27	[0.80; 2.02]	18.0%	19.2%
TRAILBLAZER-ALZ2	53	853	48	874	1.13	[0.77; 1.65]	28.7%	28.7%
Clarity_AD	49	898	46	897	1.06	[0.72; 1.57]	27.9%	26.9%
**Common effect model**		**2755**		**2726**	**1.12**	**[0.91; 1.37]**	**100.0%**	**.**
**Random effects model**					**1.12**	**[0.91; 1.37]**	**.**	**100.0%**
**Prediction interval**						**[0.72; 1.74]**		

Heterogeneity: $I^2 = 0\%$, $\tau^2 = 0$, $p = 0.93$

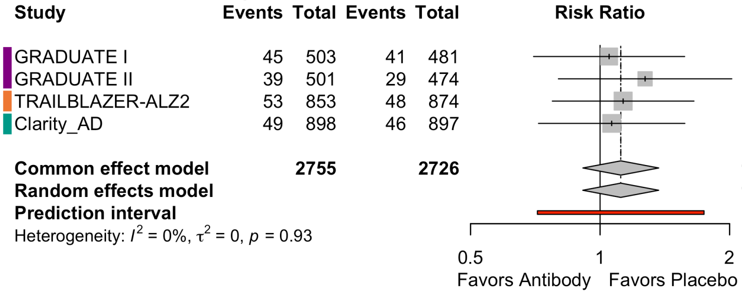

Gantenerumab **Donanemab** **Lecanemab**

S36 Figure: Sensitivity analysis 5 (excluding solanezumab, bapineuzumab, and aducanumab). Forest plots for safety outcomes related to clinical symptoms.
