# Supplementary material for: Efficacy and safety of passive immunotherapies targeting amyloid beta in Alzheimer’s disease: A systematic review and meta-analysis
Source: PLoS Med. 2025 Mar 31;22(3):e1004568. doi: 10.1371/journal.pmed.1004568 (PMC12002640; doi:10.1371/journal.pmed.1004568)
Supplement: S38 Fig — (a) Death, (b) Serious Adverse Event, (c) ARIA-E, (d) ARIA-H. ARIA-E, Amyloid-Related Imaging Abnormalities-Effusion; ARIA-H, Amyloid-Related Imaging Abnormalities-Hemorrhage. (PDF) [file pmed.1004568.s039.pdf]

(a) Death

Study	Experimental Events	Experimental Total	Control Events	Control Total	RR	95%-CI	Weight (common)	Weight (random)
GRADUATE I	3	503	10	481	0.29	[0.08; 1.04]	11.1%	7.4%
GRADUATE II	7	501	4	474	1.66	[0.49; 5.62]	4.5%	8.0%
TRAILBLAZER-ALZ2	16	853	10	874	1.64	[0.75; 3.59]	10.7%	13.5%
Clarity_AD	6	898	7	897	0.86	[0.29; 2.54]	7.6%	9.3%
EMERGE_low	0	544	5	547	0.09	[0.01; 1.65]	6.0%	1.9%
ENGAGE_low	3	549	0	540	6.89	[0.36; 132.98]	0.5%	1.8%
EXPEDITION3	9	1054	17	1067	0.54	[0.24; 1.20]	18.4%	13.2%
EXPEDITION1 & 2	24	1027	19	1025	1.26	[0.69; 2.29]	20.7%	17.0%
Study 3000	1	267	3	344	0.43	[0.04; 4.11]	2.8%	3.0%
Study 3001	4	654	4	439	0.67	[0.17; 2.67]	5.2%	6.7%
Study 301	4	337	7	524	0.89	[0.26; 3.01]	6.0%	8.0%
Study 302	15	673	5	448	2.00	[0.73; 5.46]	6.5%	10.3%
**Common effect model**		**7860**		**7660**	**0.98**	**[0.73; 1.31]**	**100.0%**	.
**Random effects model**					**0.96**	**[0.63; 1.45]**	.	**100.0%**
**Prediction interval**						**[0.34; 2.67]**		

Heterogeneity: $I^2 = 30\%$, $\tau^2 = 0.1677$, $p = 0.15$

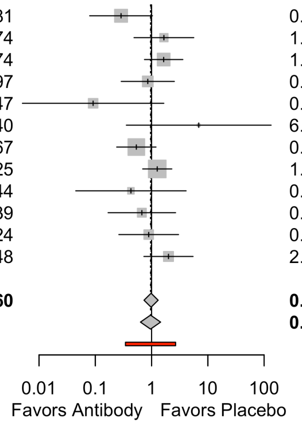

(b) Serious Adverse Event

Study	Experimental Events	Experimental Total	Control Events	Control Total	RR	95%-CI	Weight (common)	Weight (random)
GRADUATE I	76	503	95	481	0.77	[0.58; 1.01]	8.1%	8.5%
GRADUATE II	61	501	63	474	0.92	[0.66; 1.27]	5.4%	6.7%
TRAILBLAZER-ALZ2	148	853	138	874	1.10	[0.89; 1.36]	11.3%	11.4%
Clarity_AD	126	898	101	897	1.25	[0.98; 1.59]	8.4%	9.8%
EMERGE_low	72	544	81	547	0.89	[0.67; 1.20]	6.7%	7.8%
ENGAGE_low	76	549	70	540	1.07	[0.79; 1.44]	5.9%	7.5%
EXPEDITION3	175	1054	202	1067	0.88	[0.73; 1.05]	16.6%	13.1%
EXPEDITION1 & 2	261	1027	263	1025	0.99	[0.85; 1.15]	21.8%	15.4%
Study 301	72	337	108	524	1.04	[0.80; 1.35]	7.0%	8.9%
Study 302	176	673	89	448	1.32	[1.05; 1.65]	8.9%	10.7%
**Common effect model**		**6939**		**6877**	**1.01**	**[0.94; 1.09]**	**100.0%**	.
**Random effects model**					**1.01**	**[0.92; 1.12]**	.	**100.0%**
**Prediction interval**						**[0.77; 1.34]**		

Heterogeneity: $I^2 = 44\%$, $\tau^2 = 0.0117$, $p = 0.06$

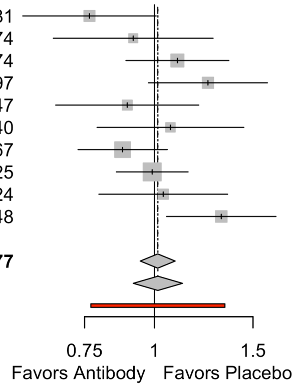

(c) ARIA-E

Study	Experimental Events	Experimental Total	Control Events	Control Total	RR	95%-CI	Weight (common)	Weight (random)
GRADUATE I	119	497	8	476	14.25	[7.04; 28.83]	7.5%	9.8%
GRADUATE II	128	496	18	470	6.74	[4.18; 10.86]	17.0%	10.6%
TRAILBLAZER-ALZ2	205	853	18	874	11.67	[7.28; 18.72]	16.4%	10.6%
Clarity_AD	113	898	15	897	7.52	[4.43; 12.79]	13.8%	10.4%
EMERGE_low	140	537	13	544	10.91	[6.26; 19.02]	11.9%	10.4%
ENGAGE_low	141	545	16	532	8.60	[5.20; 14.23]	14.9%	10.5%
EXPEDITION3	1	1054	2	1067	0.51	[0.05; 5.57]	1.8%	3.8%
EXPEDITION1 & 2	9	1027	4	1025	2.25	[0.69; 7.27]	3.7%	7.7%
Study 3000	13	267	2	344	8.37	[1.91; 36.79]	1.6%	6.5%
Study 3001	109	654	9	439	8.13	[4.16; 15.87]	9.9%	9.9%
Study 301	14	337	1	524	21.77	[2.88; 164.78]	0.7%	4.7%
Study 302	103	448	1	673	154.73	[21.67; 1104.90]	0.7%	4.9%
**Common effect model**		**7613**		**7865**	**10.07**	**[8.31; 12.21]**	**100.0%**	.
**Random effects model**					**8.88**	**[5.00; 15.77]**	.	**100.0%**
**Prediction interval**						**[1.16; 67.93]**		

Heterogeneity: $I^2 = 56\%$, $\tau^2 = 0.7479$, $p < 0.01$

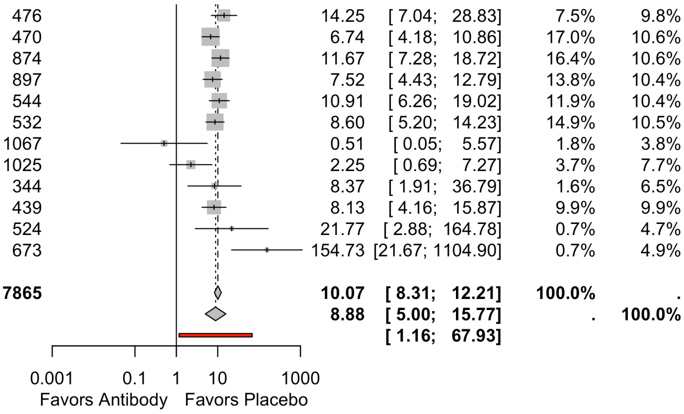

(d) ARIA-H

Study	Experimental Events	Experimental Total	Control Events	Control Total	RR	95%-CI	Weight (common)	Weight (random)
GRADUATE I	118	497	59	476	1.92	[1.44; 2.55]	13.5%	14.7%
GRADUATE II	109	496	57	470	1.81	[1.35; 2.43]	13.1%	14.5%
TRAILBLAZER-ALZ2	268	853	119	874	2.31	[1.90; 2.80]	26.4%	16.3%
Clarity_AD	155	898	81	897	1.91	[1.49; 2.46]	18.2%	15.3%
EMERGE_low	87	537	37	544	2.38	[1.65; 3.43]	8.2%	13.2%
ENGAGE_low	89	545	34	532	2.56	[1.75; 3.72]	7.7%	13.0%
EXPEDITION1 & 2	50	1027	57	1025	0.88	[0.60; 1.27]	12.8%	13.1%
**Common effect model**		**4853**		**4818**	**1.96**	**[1.76; 2.18]**	**100.0%**	.
**Random effects model**					**1.89**	**[1.46; 2.44]**	.	**100.0%**
**Prediction interval**						**[0.80; 4.48]**		

Heterogeneity: $I^2 = 75\%$, $\tau^2 = 0.0957$, $p < 0.01$

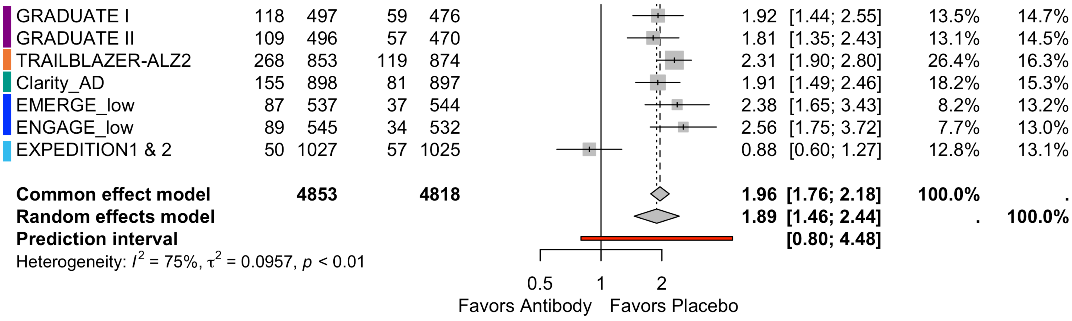

Gantenerumab **Donanemab** **Lecanemab** **Aducanumab** **Solanezumab** **Beprineuzumab**

S38 Figure: Forest plots for efficacy outcomes (low-dose populations).

(a) Headache

Study	Experimental Events	Experimental Total	Control Events	Control Total	RR	95%-CI	Weight (common)	Weight (random)
GRADUATE I	60	503	43	481	1.33	[0.92; 1.93]	6.5%	6.7%
GRADUATE II	64	501	50	474	1.21	[0.86; 1.72]	7.6%	7.6%
TRAILBLAZER-ALZ2	119	853	86	874	1.42	[1.09; 1.84]	12.5%	13.6%
Clarity_AD	100	898	73	897	1.37	[1.03; 1.82]	10.8%	11.2%
EMERGE	110	544	84	547	1.32	[1.02; 1.70]	12.3%	13.9%
ENGAGE	99	549	81	540	1.20	[0.92; 1.57]	12.0%	12.8%
EXPEDITION3	87	1054	88	1067	1.00	[0.75; 1.33]	12.9%	11.5%
EXPEDITION1 & 2	72	1027	77	1025	0.93	[0.68; 1.27]	11.3%	9.7%
Study 301	30	337	49	524	0.95	[0.62; 1.47]	5.6%	4.9%
Study 302	78	673	48	448	1.08	[0.77; 1.52]	8.5%	8.0%
**Common effect model**		**6939**		**6877**	**1.19**	**[1.08; 1.31]**	**100.0%**	**.**
**Random effects model**					**1.19**	**[1.08; 1.31]**	**.**	**100.0%**
**Prediction interval**						**[1.06; 1.34]**		

Heterogeneity: $I^2 = 0\%$, $\tau^2 = 0$, $p = 0.46$

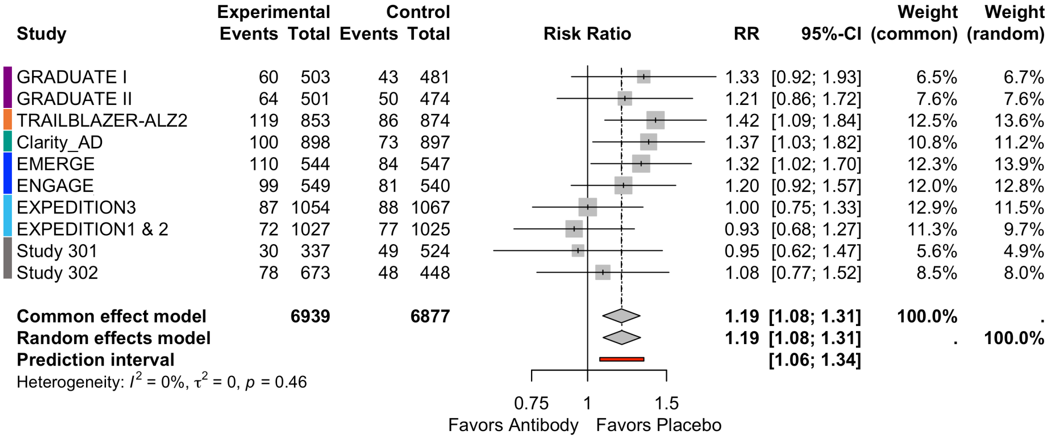

(b) Fall

Study	Experimental Events	Experimental Total	Control Events	Control Total	RR	95%-CI	Weight (common)	Weight (random)
GRADUATE I	63	503	62	481	0.97	[0.70; 1.35]	7.5%	7.4%
GRADUATE II	50	501	52	474	0.91	[0.63; 1.31]	6.4%	5.9%
TRAILBLAZER-ALZ2	114	853	110	874	1.06	[0.83; 1.36]	12.9%	13.3%
Clarity_AD	93	898	86	897	1.08	[0.82; 1.43]	10.2%	10.3%
EMERGE	68	544	71	547	0.96	[0.71; 1.31]	8.4%	8.2%
ENGAGE	80	549	57	540	1.38	[1.00; 1.90]	6.8%	7.9%
EXPEDITION3	162	1054	161	1067	1.02	[0.83; 1.25]	19.0%	19.7%
EXPEDITION1 & 2	92	1027	107	1025	0.86	[0.66; 1.12]	12.7%	11.3%
Study 301	43	337	73	524	0.92	[0.64; 1.30]	6.8%	6.5%
Study 302	100	673	64	448	1.04	[0.78; 1.39]	9.1%	9.5%
**Common effect model**		**6939**		**6877**	**1.01**	**[0.93; 1.11]**	**100.0%**	**.**
**Random effects model**					**1.01**	**[0.93; 1.11]**	**.**	**100.0%**
**Prediction interval**						**[0.91; 1.13]**		

Heterogeneity: $I^2 = 0\%$, $\tau^2 = 0$, $p = 0.71$

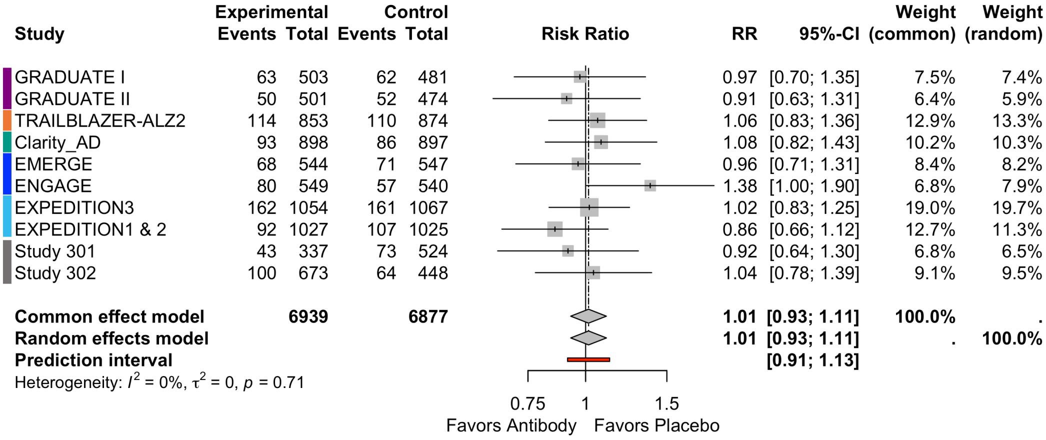

(c) Dizziness

Study	Experimental Events	Experimental Total	Control Events	Control Total	RR	95%-CI	Weight (common)	Weight (random)
GRADUATE I	45	503	41	481	1.05	[0.70; 1.57]	10.1%	10.3%
GRADUATE II	39	501	29	474	1.27	[0.80; 2.02]	7.2%	7.8%
TRAILBLAZER-ALZ2	53	853	48	874	1.13	[0.77; 1.65]	11.4%	11.7%
Clarity_AD	49	898	46	897	1.06	[0.72; 1.57]	11.1%	11.0%
EMERGE	42	544	44	547	0.96	[0.64; 1.44]	10.6%	10.2%
ENGAGE	49	549	54	540	0.89	[0.62; 1.29]	13.1%	12.4%
EXPEDITION3	62	1054	58	1067	1.08	[0.76; 1.53]	13.9%	13.9%
EXPEDITION1 & 2	58	1027	57	1025	1.02	[0.71; 1.45]	13.7%	13.3%
Study 302	54	673	31	448	1.16	[0.76; 1.77]	9.0%	9.3%
**Common effect model**		**6602**		**6353**	**1.06**	**[0.93; 1.20]**	**100.0%**	**.**
**Random effects model**					**1.06**	**[0.93; 1.20]**	**.**	**100.0%**
**Prediction interval**						**[0.90; 1.23]**		

Heterogeneity: $I^2 = 0\%$, $\tau^2 = 0$, $p = 0.98$

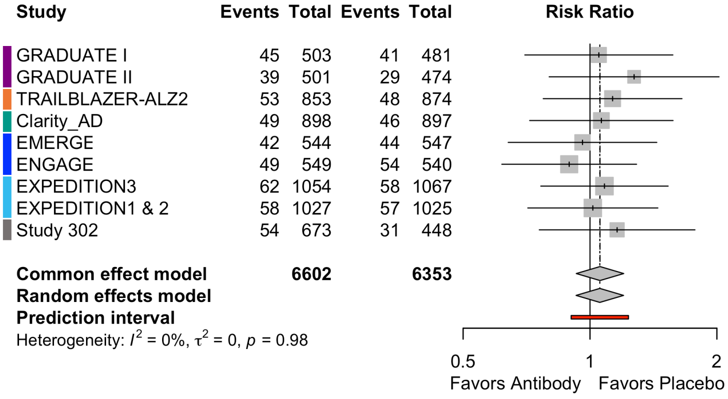

Gantenerumab **Donanemab** **Lecanemab** **Aducanumab** **Solanezumab** **Beprineuzumab**

S39 Figure: Forest plots for safety outcomes related to symptoms (low-dose populations).

(a) Cerebral macrohemorrhage: low dose

Study	Experimental Events	Experimental Total	Control Events	Control Total	Risk Ratio	RR	95%-CI	Weight (common)	Weight (random)
GRADUATE I	4	481	8	503		0.52	[0.16; 1.73]	47.6%	27.9%
GRADUATE II	10	501	2	474		4.73	[1.04; 21.48]	12.5%	18.8%
TRAILBLAZER-ALZ2	3	853	2	874		1.54	[0.26; 9.18]	12.0%	14.0%
Clarity_AD	5	898	1	897		4.99	[0.58; 42.66]	6.1%	10.0%
Study 3000	0	267	0	344				0.0%	0.0%
Study 3001	5	439	2	439		2.50	[0.49; 12.82]	12.2%	16.4%
Study 300	1	337	0	524		4.66	[0.19; 114.11]	2.4%	4.7%
Study 301	2	673	1	448		1.33	[0.12; 14.64]	7.3%	8.1%
**Common effect model**		**4449**		**4503**		**1.84**	**[1.01; 3.35]**	**100.0%**	**--**
**Random effects model**						**1.78**	**[0.88; 3.61]**	**--**	**100.0%**
**Prediction interval**							**[0.53; 6.01]**		

Heterogeneity: $I^2 = 16\%$, $\tau^2 = 0.0936$, $p = 0.31$

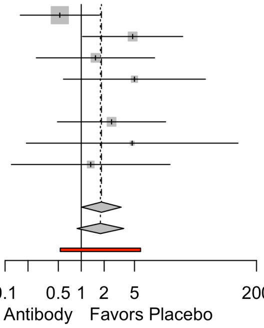

(b) Cerebral macrohemorrhage: high dose

Study	Experimental Events	Experimental Total	Control Events	Control Total	Risk Ratio	RR	95%-CI	Weight (common)	Weight (random)
GRADUATE I	4	481	8	503		0.52	[0.16; 1.73]	47.6%	27.9%
GRADUATE II	10	501	2	474		4.73	[1.04; 21.48]	12.5%	18.8%
TRAILBLAZER-ALZ2	3	853	2	874		1.54	[0.26; 9.18]	12.0%	14.0%
Clarity_AD	5	898	1	897		4.99	[0.58; 42.66]	6.1%	10.0%
Study 3000	0	263	0	344				0.0%	0.0%
Study 3001	5	439	2	439		2.50	[0.49; 12.82]	12.2%	16.4%
Study 300	1	329	0	524		4.78	[0.20; 116.88]	2.3%	4.7%
Study 301	2	673	1	448		1.33	[0.12; 14.64]	7.3%	8.2%
**Common effect model**		**4437**		**4503**		**1.84**	**[1.01; 3.35]**	**100.0%**	**--**
**Random effects model**						**1.79**	**[0.88; 3.62]**	**--**	**100.0%**
**Prediction interval**							**[0.53; 6.05]**		

Heterogeneity: $I^2 = 16\%$, $\tau^2 = 0.0953$, $p = 0.31$

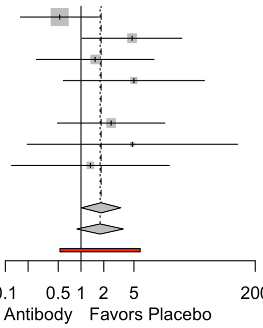

Gantenerumab **Donanemab** **Lecanemab** **Beprineuzumab**

S40 Figure: Forest plot for cerebral macrohemorrhage (low-dose and high-dose populations).

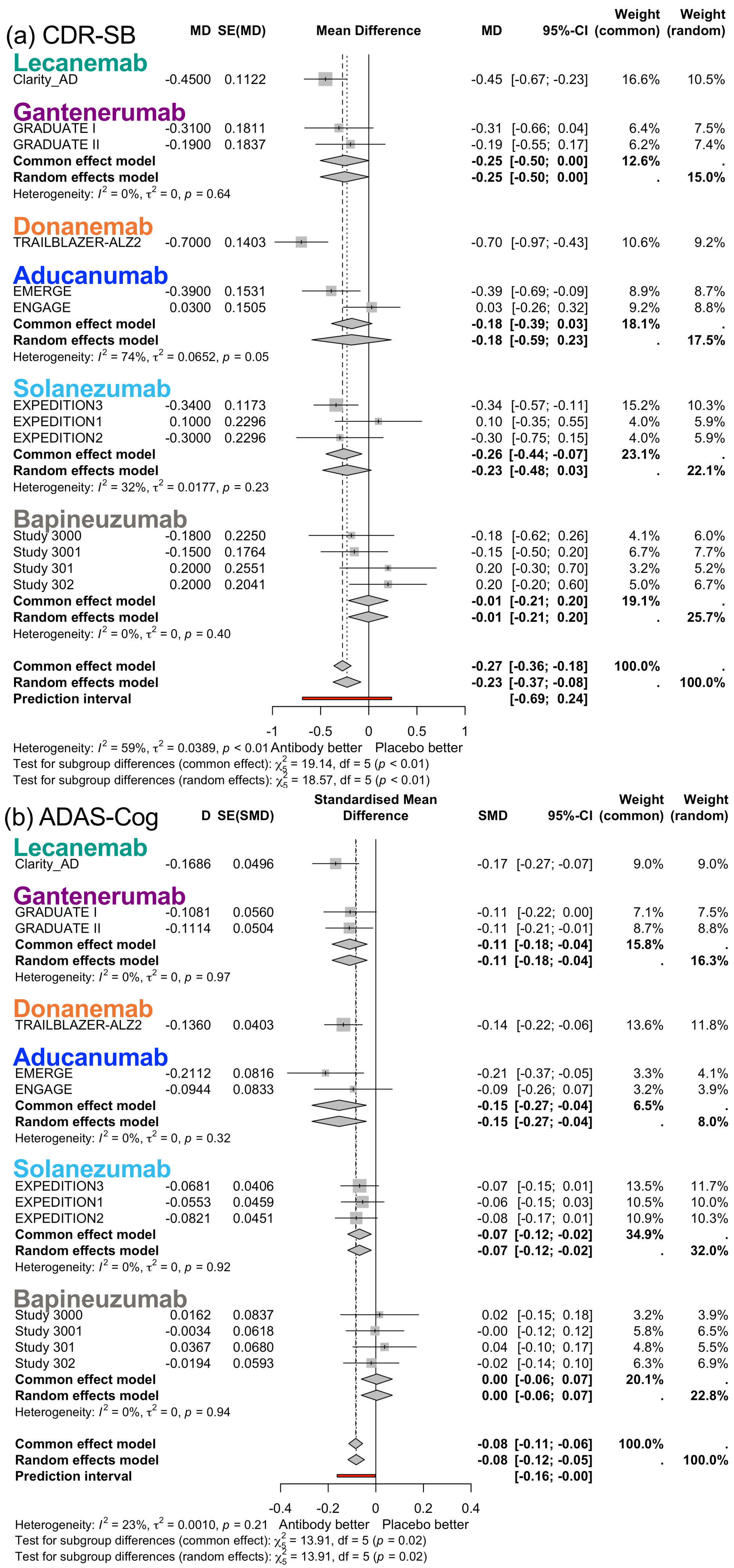

S41 Figure: Forest plots for efficacy outcomes (high-dose populations).

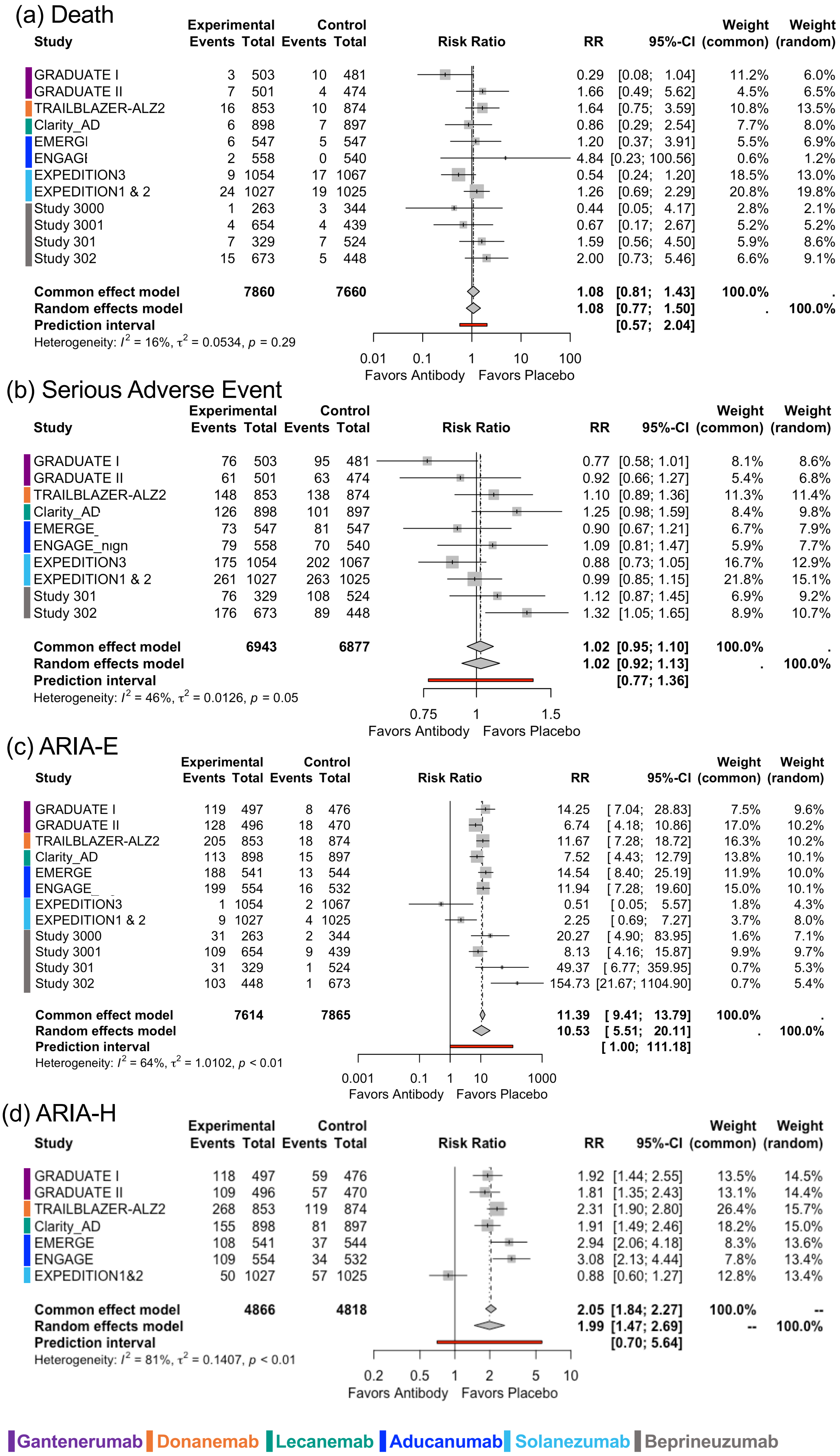

S42 Figure: Forest plots for safety outcomes (high-dose populations).

(a) Headache

Study	Experimental Events	Experimental Total	Control Events	Control Total	RR	95%-CI	Weight (common)	Weight (random)
GRADUATE I	60	503	43	481	1.33	[0.92; 1.93]	6.5%	6.6%
GRADUATE II	64	501	50	474	1.21	[0.86; 1.72]	7.6%	7.5%
TRAILBLAZER-ALZ2	119	853	86	874	1.42	[1.09; 1.84]	12.5%	13.4%
Clarity_AD	100	898	73	897	1.37	[1.03; 1.82]	10.7%	11.1%
EMERGE	107	547	84	547	1.27	[0.98; 1.65]	12.4%	13.5%
ENGAGE	115	558	81	540	1.37	[1.06; 1.78]	12.1%	13.7%
EXPEDITION3	87	1054	88	1067	1.00	[0.75; 1.33]	12.9%	11.3%
EXPEDITION1 & 2	72	1027	77	1025	0.93	[0.68; 1.27]	11.3%	9.5%
Study 301	34	329	49	524	1.11	[0.73; 1.67]	5.6%	5.3%
Study 302	78	673	48	448	1.08	[0.77; 1.52]	8.5%	7.9%
**Common effect model**		**6943**		**6877**	**1.21**	**[1.10; 1.34]**	**100.0%**	**.**
**Random effects model**					**1.22**	**[1.11; 1.34]**	**.**	**100.0%**
**Prediction interval**						**[1.09; 1.36]**		

Heterogeneity: $I^2 = 0\%$, $\tau^2 = 0$, $p = 0.49$

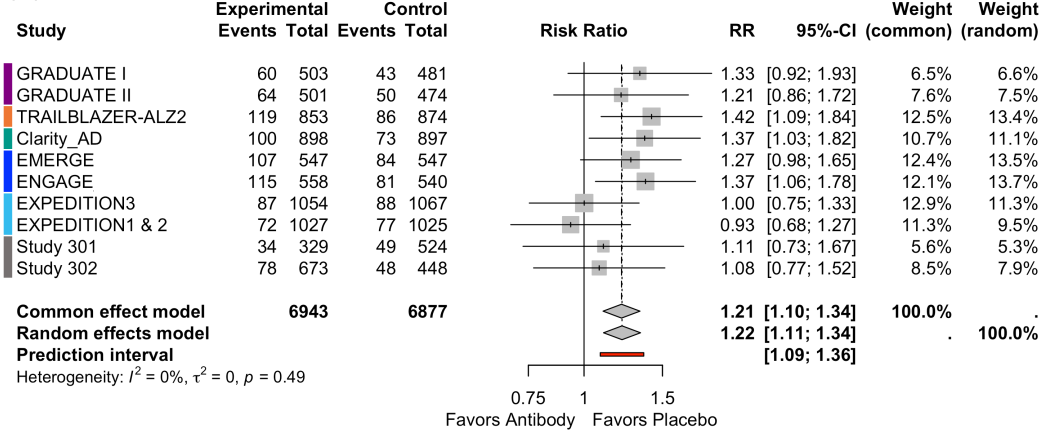

(b) Fall

Study	Experimental Events	Experimental Total	Control Events	Control Total	RR	95%-CI	Weight (common)	Weight (random)
GRADUATE I	63	503	62	481	0.97	[0.70; 1.35]	7.5%	7.4%
GRADUATE II	50	501	52	474	0.91	[0.63; 1.31]	6.4%	5.8%
TRAILBLAZER-ALZ2	114	853	110	874	1.06	[0.83; 1.36]	12.9%	13.2%
Clarity_AD	93	898	86	897	1.08	[0.82; 1.43]	10.2%	10.2%
EMERGE	76	547	71	547	1.07	[0.79; 1.45]	8.4%	8.7%
ENGAGE	86	558	57	540	1.46	[1.07; 2.00]	6.9%	8.0%
EXPEDITION3	162	1054	161	1067	1.02	[0.83; 1.25]	19.0%	19.6%
EXPEDITION1 & 2	92	1027	107	1025	0.86	[0.66; 1.12]	12.7%	11.2%
Study 301	43	329	73	524	0.94	[0.66; 1.33]	6.7%	6.4%
Study 302	100	673	64	448	1.04	[0.78; 1.39]	9.1%	9.4%
**Common effect model**		**6943**		**6877**	**1.03**	**[0.94; 1.13]**	**100.0%**	**.**
**Random effects model**					**1.03**	**[0.94; 1.13]**	**.**	**100.0%**
**Prediction interval**						**[0.93; 1.14]**		

Heterogeneity: $I^2 = 0\%$, $\tau^2 = 0$, $p = 0.57$

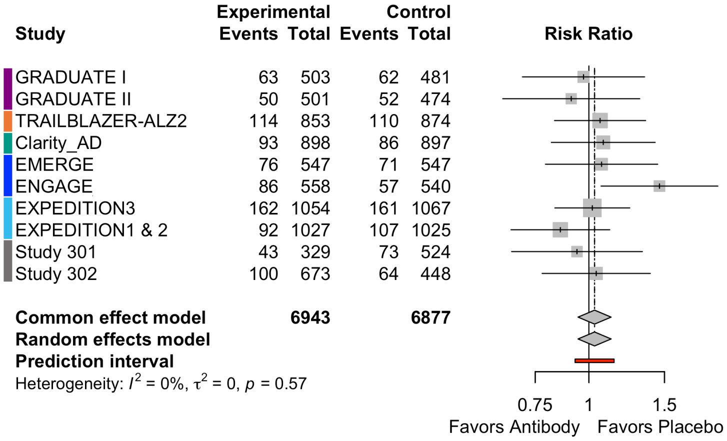

(c) Dizziness

Study	Experimental Events	Experimental Total	Control Events	Control Total	RR	95%-CI	Weight (common)	Weight (random)
GRADUATE I	45	503	41	481	1.05	[0.70; 1.57]	10.1%	10.1%
GRADUATE II	39	501	29	474	1.27	[0.80; 2.02]	7.2%	7.6%
TRAILBLAZER-ALZ2	53	853	48	874	1.13	[0.77; 1.65]	11.4%	11.5%
Clarity_AD	49	898	46	897	1.06	[0.72; 1.57]	11.1%	10.7%
EMERGE	55	547	44	547	1.25	[0.86; 1.82]	10.6%	11.5%
ENGAGE	54	558	54	540	0.97	[0.68; 1.38]	13.2%	12.8%
EXPEDITION3	62	1054	58	1067	1.08	[0.76; 1.53]	13.9%	13.6%
EXPEDITION1 & 2	58	1027	57	1025	1.02	[0.71; 1.45]	13.7%	13.0%
Study 302	54	673	31	448	1.16	[0.76; 1.77]	8.9%	9.1%
**Common effect model**		**6614**		**6353**	**1.10**	**[0.96; 1.25]**	**100.0%**	**.**
**Random effects model**					**1.10**	**[0.96; 1.25]**	**.**	**100.0%**
**Prediction interval**						**[0.94; 1.28]**		

Heterogeneity: $I^2 = 0\%$, $\tau^2 = 0$, $p = 0.99$

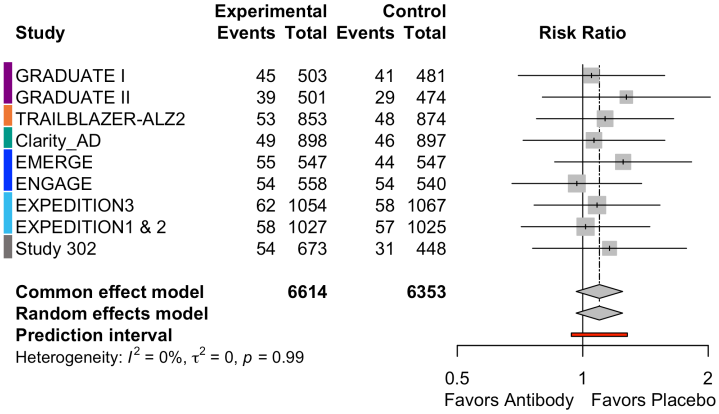

Gantenerumab **Donanemab** **Lecanemab** **Aducanumab** **Solanezumab** **Beprineuzumab**

S43 Figure: Forest plots for safety outcomes related to symptoms (high-dose populations).

(a)CDR-SB

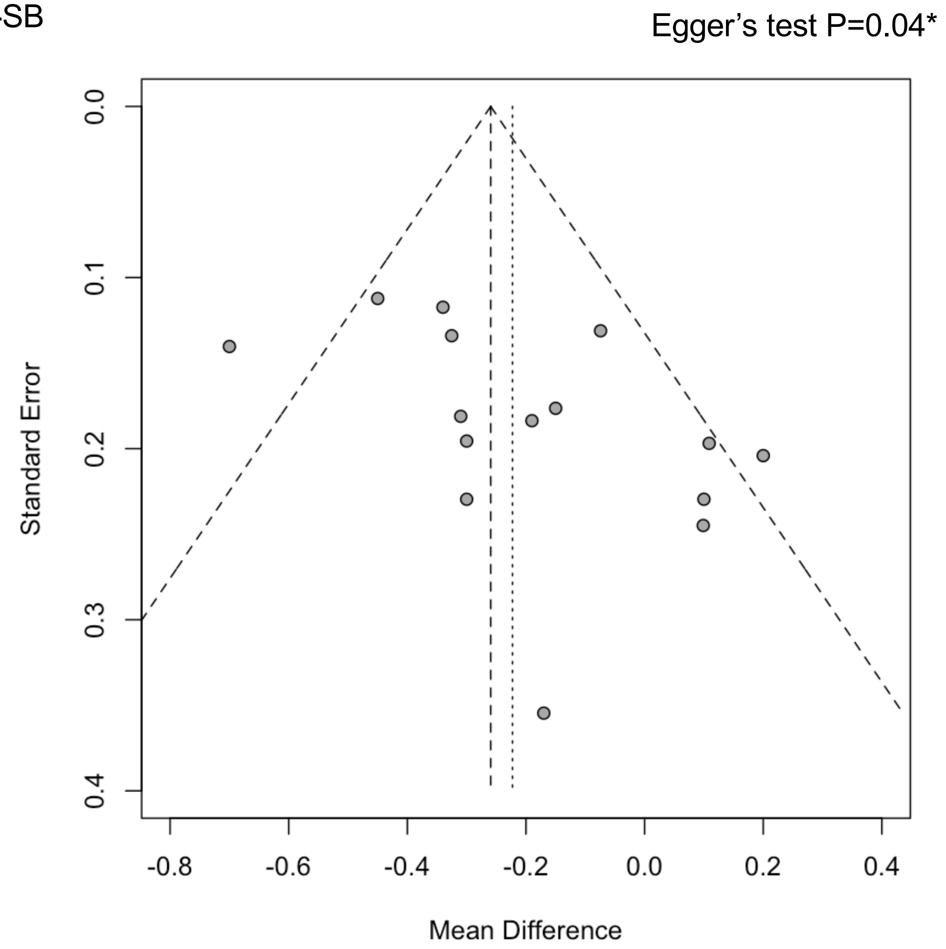

(b)ADAS-Cog

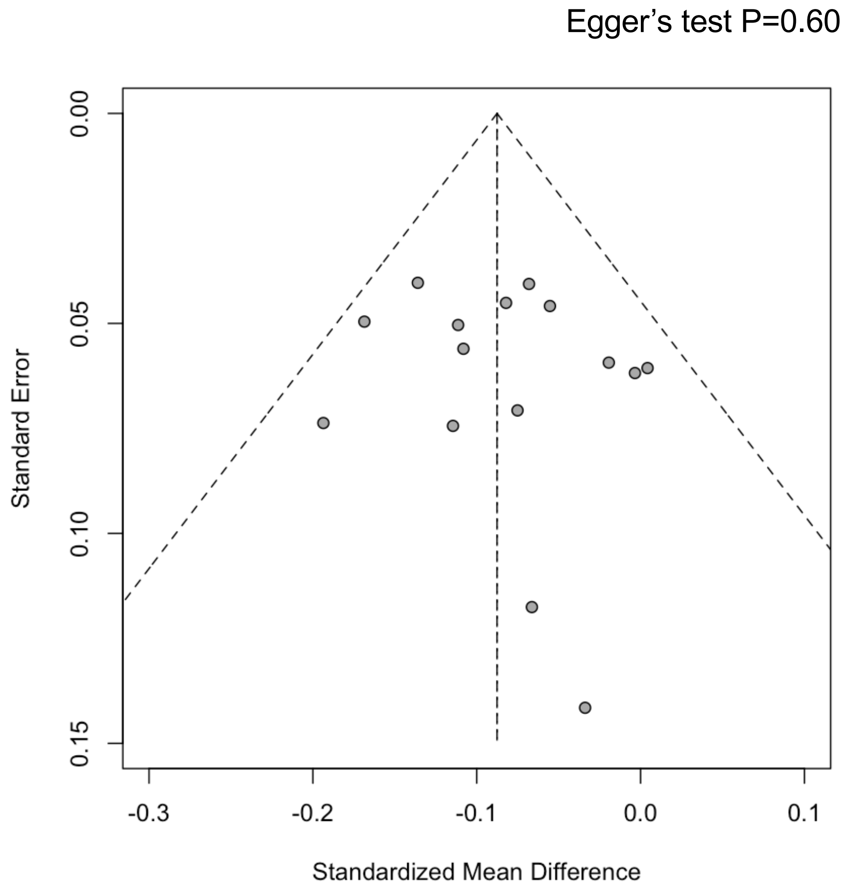

S44 Figure: Funnel plots for efficacy endpoints in sensitivity analysis 8 (including halted trials with sample size with fewer than 200 patients in each arm).

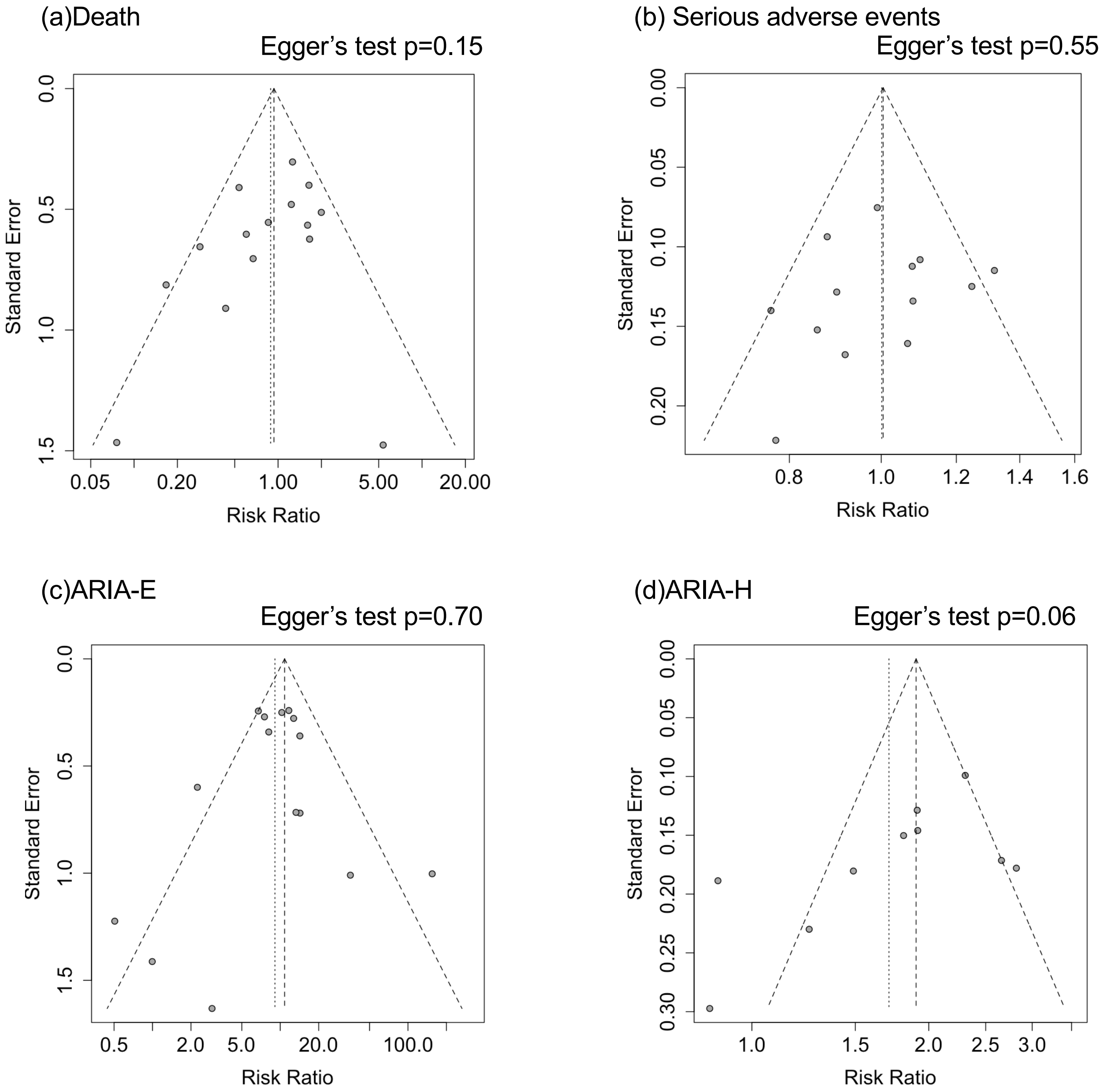

S45 Figure: Funnel plots for safety endpoints in sensitivity analysis 8 (including halted trials with a sample size of fewer than 200 patients in each arm).

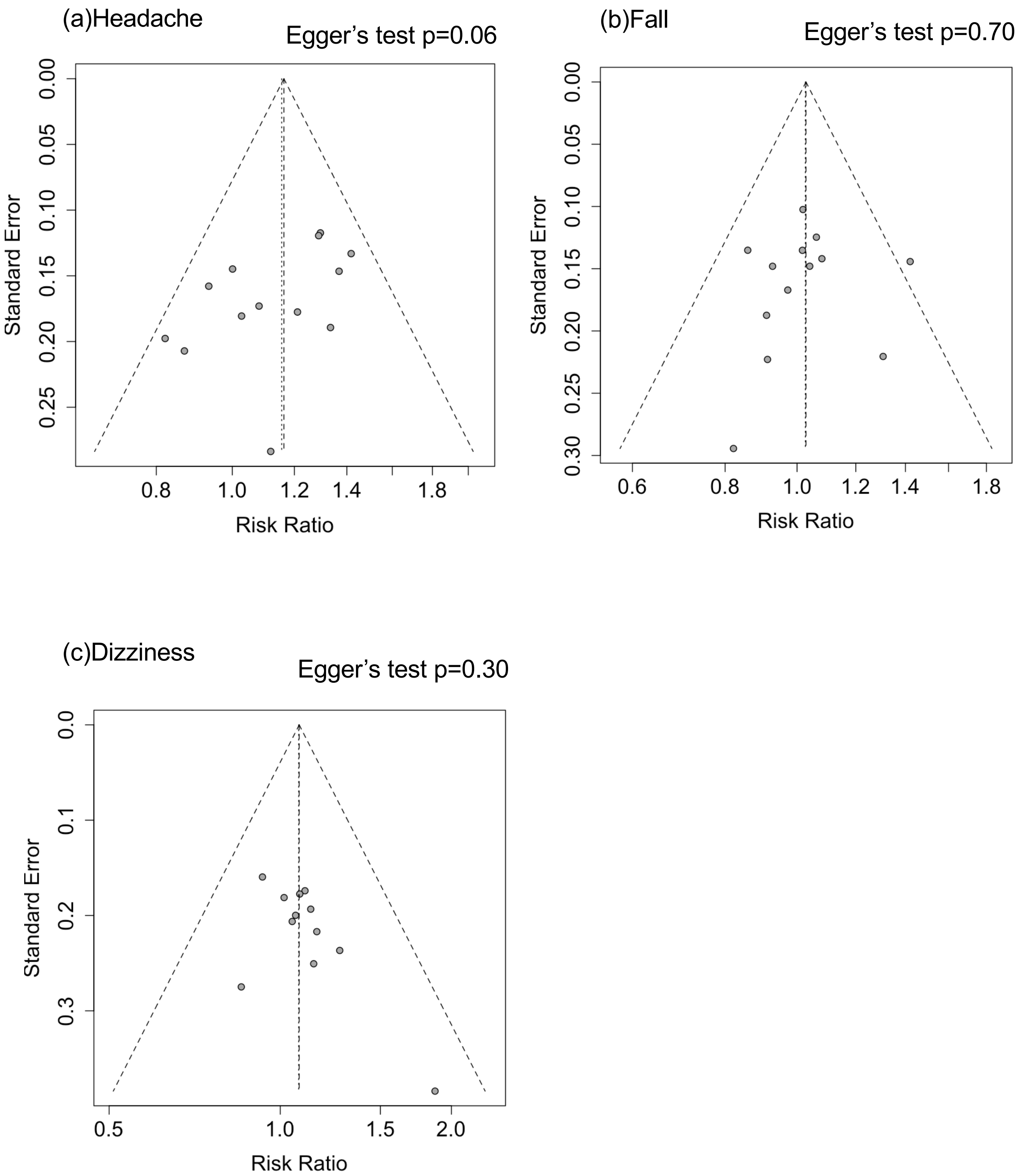

S46 Figure: Funnel plots for safety endpoints in sensitivity analysis 8 (including halted trials with a sample size of fewer than 200 patients in each arm).

CDR-SB

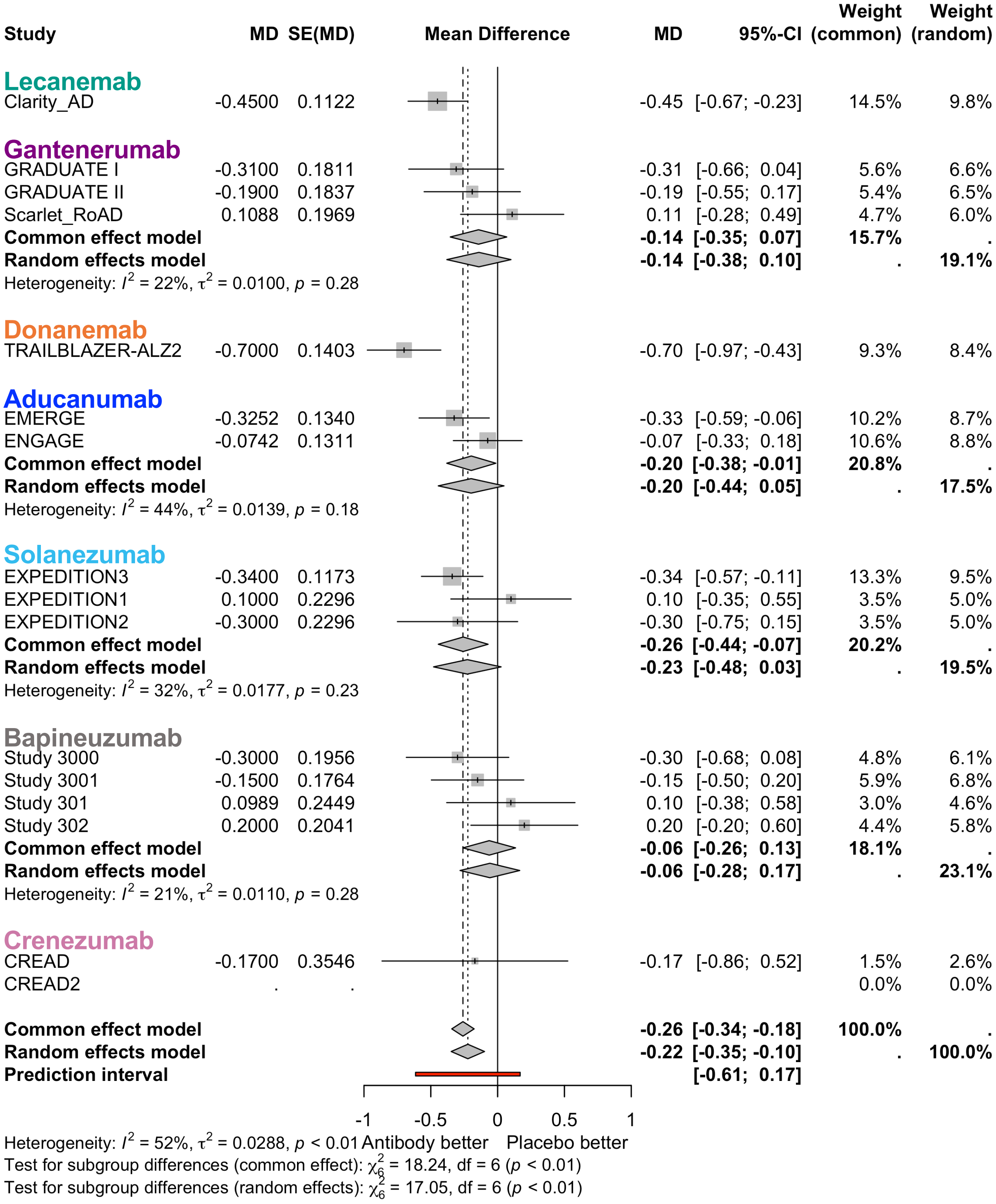

S47 Figure: Sensitivity analysis 8 (including halted trials with a sample size of fewer than 200 patients in each arm). Forest plots for the change in CDR-SB.

ADAS-Cog

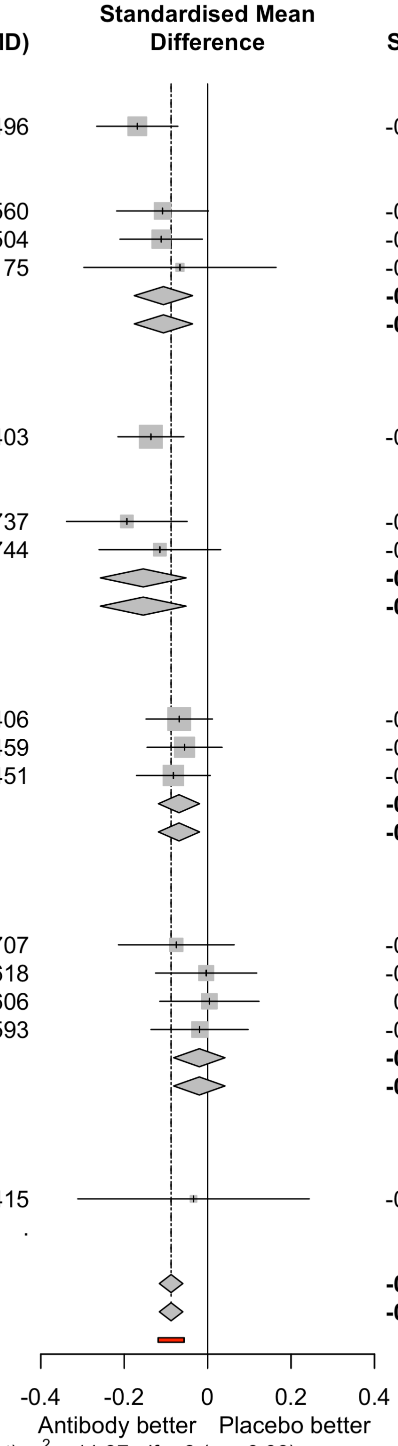

Study	SMD	SE(SMD)	SMD	95%-CI	Weight (common)	Weight (random)
**Lecanemab**						
Clarity_AD	-0.1686	0.0496	-0.17	[-0.27; -0.07]	8.5%	8.5%
**Gantenerumab**						
GRADUATE I	-0.1081	0.0560	-0.11	[-0.22; 0.00]	6.6%	6.6%
GRADUATE II	-0.1114	0.0504	-0.11	[-0.21; -0.01]	8.2%	8.2%
Scarlet_RoAD	-0.0663	0.1175	-0.07	[-0.30; 0.16]	1.5%	1.5%
**Common effect model**			**-0.11**	**[-0.18; -0.04]**	**16.3%**	**.**
**Random effects model**			**-0.11**	**[-0.18; -0.04]**	**.**	**16.3%**
Heterogeneity: $I^2 = 0\%$, $\tau^2 = 0$, $p = 0.94$						
**Donanemab**						
TRAILBLAZER-ALZ2	-0.1360	0.0403	-0.14	[-0.22; -0.06]	12.8%	12.8%
**Aducanumab**						
EMERGE	-0.1936	0.0737	-0.19	[-0.34; -0.05]	3.8%	3.8%
ENGAGE	-0.1145	0.0744	-0.11	[-0.26; 0.03]	3.8%	3.8%
**Common effect model**			**-0.15**	**[-0.26; -0.05]**	**7.6%**	**.**
**Random effects model**			**-0.15**	**[-0.26; -0.05]**	**.**	**7.6%**
Heterogeneity: $I^2 = 0\%$, $\tau^2 = 0$, $p = 0.45$						
**Solanezumab**						
EXPEDITION3	-0.0681	0.0406	-0.07	[-0.15; 0.01]	12.6%	12.6%
EXPEDITION1	-0.0553	0.0459	-0.06	[-0.15; 0.03]	9.9%	9.9%
EXPEDITION2	-0.0821	0.0451	-0.08	[-0.17; 0.01]	10.2%	10.2%
**Common effect model**			**-0.07**	**[-0.12; -0.02]**	**32.7%**	**.**
**Random effects model**			**-0.07**	**[-0.12; -0.02]**	**.**	**32.7%**
Heterogeneity: $I^2 = 0\%$, $\tau^2 = 0$, $p = 0.92$						
**Bapineuzumab**						
Study 3000	-0.0751	0.0707	-0.08	[-0.21; 0.06]	4.2%	4.2%
Study 3001	-0.0034	0.0618	-0.00	[-0.12; 0.12]	5.4%	5.4%
Study 301	0.0043	0.0606	0.00	[-0.11; 0.12]	5.7%	5.7%
Study 302	-0.0194	0.0593	-0.02	[-0.14; 0.10]	5.9%	5.9%
**Common effect model**			**-0.02**	**[-0.08; 0.04]**	**21.1%**	**.**
**Random effects model**			**-0.02**	**[-0.08; 0.04]**	**.**	**21.1%**
Heterogeneity: $I^2 = 0\%$, $\tau^2 = 0$, $p = 0.84$						
**Crenezumab**						
CREAD	-0.0339	0.1415	-0.03	[-0.31; 0.24]	1.0%	1.0%
CREAD2	.	.			0.0%	0.0%
**Common effect model**			**-0.09**	**[-0.12; -0.06]**	**100.0%**	**.**
**Random effects model**			**-0.09**	**[-0.12; -0.06]**	**.**	**100.0%**
**Prediction interval**				**[-0.12; -0.06]**		

Heterogeneity: $I^2 = 0\%$, $\tau^2 = 0$, $p = 0.52$

Test for subgroup differences (common effect): $\chi^2_6 = 11.37$, df = 6 ($p = 0.08$)

Test for subgroup differences (random effects): $\chi^2_6 = 11.37$, df = 6 ($p = 0.08$)

S48 Figure: Sensitivity analysis 8 (including halted trials with a sample size of fewer than 200 patients in each arm). Forest plots for the change in ADAS-Cog.

(a)Death

Study	Experimental Events	Experimental Total	Control Events	Control Total	RR	95%-CI	Weight (common)	Weight (random)
GRADUATE I	3	503	10	481	0.29	[0.08; 1.04]	8.8%	6.1%
GRADUATE II	7	501	4	474	1.66	[0.49; 5.62]	3.5%	6.5%
TRAILBLAZER-ALZ2	16	853	10	874	1.64	[0.75; 3.59]	8.5%	10.0%
Clarity_AD	6	898	7	897	0.86	[0.29; 2.54]	6.0%	7.4%
EMERGE	6	1091	5	547	0.60	[0.18; 1.96]	5.7%	6.8%
ENGAGE	5	1107	0	540	5.37	[0.30; 96.91]	0.6%	1.7%
EXPEDITION3	9	1054	17	1067	0.54	[0.24; 1.20]	14.5%	9.8%
EXPEDITION1 & 2	24	1027	19	1025	1.26	[0.69; 2.29]	16.4%	11.9%
Study 3000	2	530	3	344	0.43	[0.07; 2.58]	3.1%	3.9%
Study 3001	4	654	4	439	0.67	[0.17; 2.67]	4.1%	5.6%
Study 301	11	666	7	524	1.24	[0.48; 3.17]	6.7%	8.6%
Study 302	15	673	5	448	2.00	[0.73; 5.46]	5.2%	8.1%
CREAD	8	404	5	405	1.60	[0.53; 4.86]	4.3%	7.3%
CREAD2	0	404	6	398	0.08	[0.00; 1.34]	5.6%	1.8%
Scarlet_RoAD	2	531	6	266	0.17	[0.03; 0.82]	6.9%	4.6%
**Common effect**		**10896**		**8729**	**0.94**	**[0.72; 1.21]**	**100.0%**	**.**
**Random effects model**					**0.89**	**[0.59; 1.33]**	**.**	**100.0%**
**Prediction interval**						**[0.27; 2.94]**		

Heterogeneity: $I^2 = 38\%$, $\tau^2 = 0.2639$, $p = 0.07$

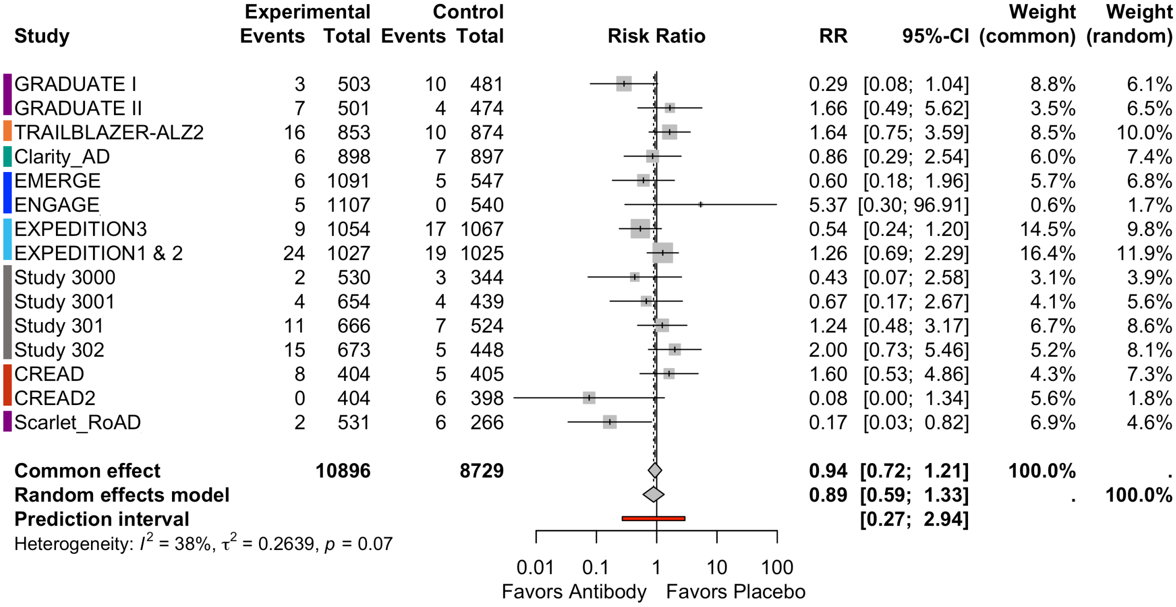

(b) Serious adverse events

Study	Experimental Events	Experimental Total	Control Events	Control Total	RR	95%-CI	Weight (common)	Weight (random)
GRADUATE I	76	503	95	481	0.77	[0.58; 1.01]	6.6%	6.8%
GRADUATE II	61	501	63	474	0.92	[0.66; 1.27]	4.4%	5.3%
TRAILBLAZER-ALZ2	148	853	138	874	1.10	[0.89; 1.36]	9.3%	9.3%
Clarity_AD	126	898	101	897	1.25	[0.98; 1.59]	6.9%	7.8%
EMERGE	145	1091	81	547	0.90	[0.70; 1.15]	7.3%	7.6%
ENGAGE	155	1107	70	540	1.08	[0.83; 1.40]	6.4%	7.2%
EXPEDITION3	175	1054	202	1067	0.88	[0.73; 1.05]	13.6%	10.7%
EXPEDITION1 & 2	261	1027	263	1025	0.99	[0.85; 1.15]	17.9%	12.8%
Study 301	148	666	108	524	1.08	[0.87; 1.34]	8.2%	8.9%
Study 302	176	673	89	448	1.32	[1.05; 1.65]	7.3%	8.7%
CREAD	67	404	63	405	1.07	[0.78; 1.46]	4.3%	5.6%
CREAD2	33	404	42	398	0.77	[0.50; 1.20]	2.9%	3.4%
Scarlet_RoAD	94	531	55	266	0.86	[0.64; 1.15]	5.0%	6.0%
**Common effect**		**9712**		**7946**	**1.00**	**[0.94; 1.07]**	**100.0%**	**.**
**Random effects model**					**1.00**	**[0.92; 1.09]**	**.**	**100.0%**
**Prediction interval**						**[0.79; 1.27]**		

Heterogeneity: $I^2 = 38\%$, $\tau^2 = 0.0099$, $p = 0.08$

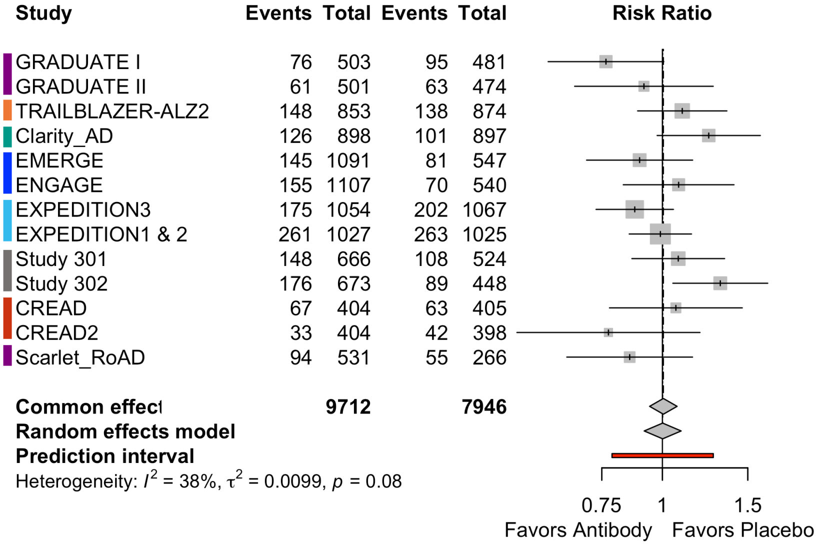

Gantenerumab **Donanemab** **Lecanemab** **Aducanumab** **Solanezumab** **Beprineuzumab** **Crenezumab**

S49 Figure: Sensitivity analysis 8 (including halted trials with a sample size of fewer than 200 patients in each arm). Forest plots for safety outcomes.

(a)ARIA-E

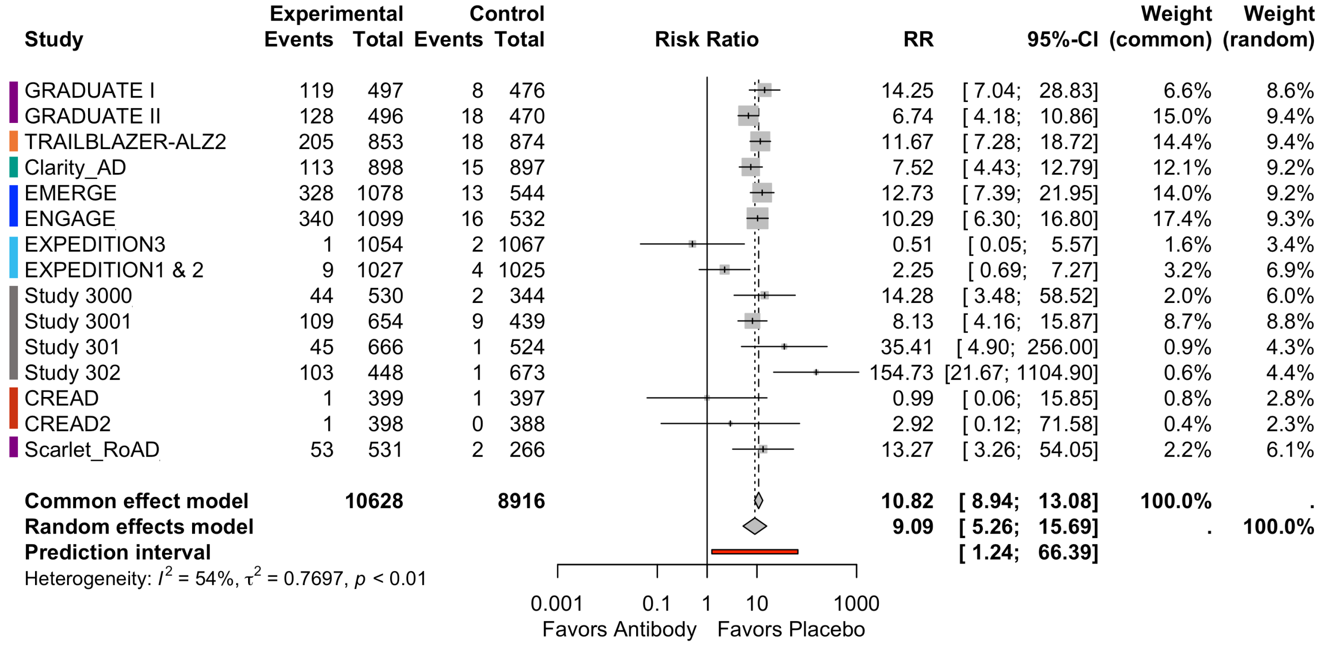

(b)ARIA-H

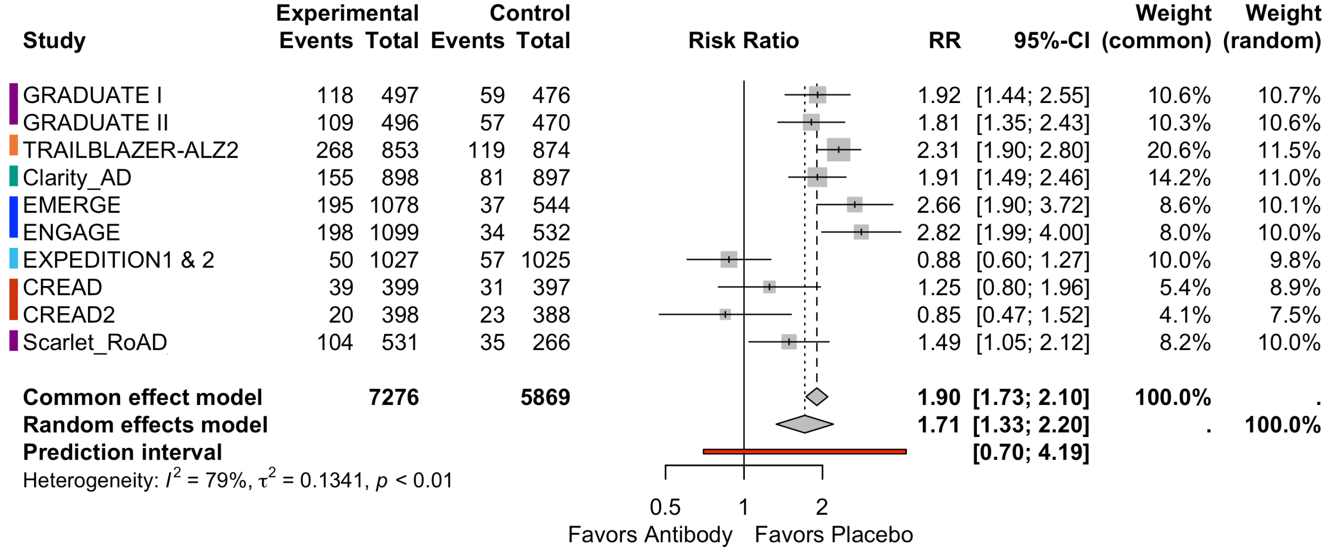

Gantenerumab **Donanemab** **Lecanemab** **Aducanumab** **Solanezumab** **Beprineuzumab** **Crenezumab**

S50 Figure: Sensitivity analysis 8 (including halted trials with a sample size of fewer than 200 patients in each arm). Forest plots for safety outcomes related to imaging abnormalities.

(a)Headache

Study	Experimental Events	Experimental Total	Control Events	Control Total	Risk Ratio	RR	95%-CI	Weight (common)	Weight (random)
GRADUATE I	60	503	43	481		1.33	[0.92; 1.93]	5.1%	5.8%
GRADUATE II	64	501	50	474		1.21	[0.86; 1.72]	5.9%	6.5%
TRAILBLAZER-ALZ2	119	853	86	874		1.42	[1.09; 1.84]	9.8%	10.5%
Clarity_AD	100	898	73	897		1.37	[1.03; 1.82]	8.4%	9.0%
EMERGE_high	217	1091	84	547		1.30	[1.03; 1.63]	12.9%	12.7%
ENGAGE_high	214	1107	81	540		1.29	[1.02; 1.63]	12.6%	12.3%
EXPEDITION3	87	1054	88	1067		1.00	[0.75; 1.33]	10.1%	9.1%
EXPEDITION1 & 2	72	1027	77	1025		0.93	[0.68; 1.27]	8.9%	7.9%
Study 301_high	64	666	49	524		1.03	[0.72; 1.46]	6.3%	6.3%
Study 302	78	673	48	448		1.08	[0.77; 1.52]	6.7%	6.8%
CREAD	39	404	45	405		0.87	[0.58; 1.30]	5.2%	4.9%
CREAD2	25	404	22	398		1.12	[0.64; 1.95]	2.6%	2.8%
Scarlet_RoAD_high	59	531	36	266		0.82	[0.56; 1.21]	5.5%	5.4%
**Common effect model**		**9712**		**7946**		**1.16**	**[1.07; 1.27]**	**100.0%**	**.**
**Random effects model**						**1.16**	**[1.05; 1.27]**	**.**	**100.0%**
**Prediction interval**							**[0.96; 1.40]**		

Heterogeneity: $I^2 = 17\%$, $\tau^2 = 0.0049$, $p = 0.28$

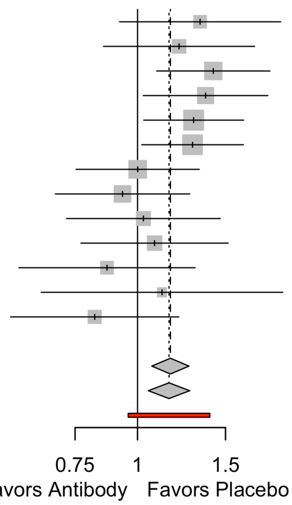

(b)Fall

Study	Experimental Events	Experimental Total	Control Events	Control Total	Risk Ratio	RR	95%-CI	Weight (common)	Weight (random)
GRADUATE I	63	503	62	481		0.97	[0.70; 1.35]	6.3%	6.2%
GRADUATE II	50	501	52	474		0.91	[0.63; 1.31]	5.3%	5.0%
TRAILBLAZER-ALZ2	114	853	110	874		1.06	[0.83; 1.36]	10.8%	11.2%
Clarity_AD	93	898	86	897		1.08	[0.82; 1.43]	8.6%	8.6%
EMERGE_high	144	1091	71	547		1.02	[0.78; 1.33]	9.4%	9.5%
ENGAGE_high	166	1107	57	540		1.42	[1.07; 1.88]	7.6%	8.4%
EXPEDITION3	162	1054	161	1067		1.02	[0.83; 1.25]	16.0%	16.6%
EXPEDITION1 & 2	92	1027	107	1025		0.86	[0.66; 1.12]	10.7%	9.5%
Study 301_high	86	666	73	524		0.93	[0.69; 1.24]	8.1%	7.9%
Study 302	100	673	64	448		1.04	[0.78; 1.39]	7.7%	7.9%
CREAD	43	404	33	405		1.31	[0.85; 2.01]	3.3%	3.6%
CREAD2	20	404	24	398		0.82	[0.46; 1.46]	2.4%	2.0%
Scarlet_RoAD_high	51	531	28	266		0.91	[0.59; 1.41]	3.7%	3.5%
**Common effect model**		**9712**		**7946**		**1.03**	**[0.95; 1.12]**	**100.0%**	**.**
**Random effects model**						**1.03**	**[0.95; 1.11]**	**.**	**100.0%**
**Prediction interval**							**[0.94; 1.12]**		

Heterogeneity: $I^2 = 0\%$, $\tau^2 = 0$, $p = 0.61$

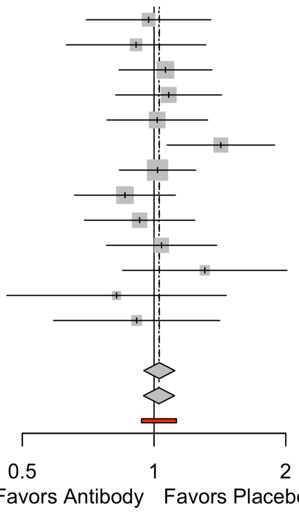

(c)Dizziness

Study	Experimental Events	Experimental Total	Control Events	Control Total	Risk Ratio	RR	95%-CI	Weight (common)	Weight (random)
GRADUATE I	45	503	41	481		1.05	[0.70; 1.57]	8.2%	8.3%
GRADUATE II	39	501	29	474		1.27	[0.80; 2.02]	5.8%	6.3%
TRAILBLAZER-ALZ2	53	853	48	874		1.13	[0.77; 1.65]	9.2%	9.4%
Clarity_AD	49	898	46	897		1.06	[0.72; 1.57]	9.0%	8.8%
EMERGE_high	97	1091	44	547		1.11	[0.79; 1.55]	11.4%	11.6%
ENGAGE_high	103	1107	54	540		0.93	[0.68; 1.27]	14.1%	13.8%
EXPEDITION3	62	1054	58	1067		1.08	[0.76; 1.53]	11.2%	11.2%
EXPEDITION1 & 2	58	1027	57	1025		1.02	[0.71; 1.45]	11.1%	10.7%
Study 302	54	673	31	448		1.16	[0.76; 1.77]	7.3%	7.5%
CREAD	23	404	27	405		0.85	[0.50; 1.46]	5.3%	4.6%
CREAD2	19	404	10	398		1.87	[0.88; 3.97]	2.0%	2.4%
Scarlet_RoAD_high	48	531	21	266		1.15	[0.70; 1.87]	5.5%	5.6%
**Common effect model**		**9046**		**7422**		**1.08**	**[0.96; 1.21]**	**100.0%**	**.**
**Random effects model**						**1.08**	**[0.96; 1.21]**	**.**	**100.0%**
**Prediction interval**							**[0.94; 1.23]**		

Heterogeneity: $I^2 = 0\%$, $\tau^2 = 0$, $p = 0.95$

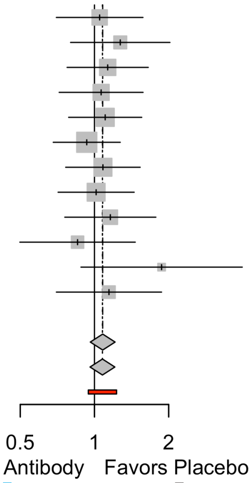

Gantenerumab **Donanemab** **Lecanemab** **Aducanumab** **Solanezumab** **Beprineuzumab** **Crenezumab**

S51 Figure: Sensitivity analysis 8 (including halted trials with a sample size of fewer than 200 patients in each arm). Forest plots for safety outcomes related to symptoms.
