# Supplementary material for: Efficacy and safety of passive immunotherapies targeting amyloid beta in Alzheimer’s disease: A systematic review and meta-analysis
Source: PLoS Med. 2025 Mar 31;22(3):e1004568. doi: 10.1371/journal.pmed.1004568 (PMC12002640; doi:10.1371/journal.pmed.1004568)
Supplement: S39 Fig — (a) Headache, (b) Fall, and (c) Dizziness. (PDF) [file pmed.1004568.s040.pdf]

(a) Headache

Study	Experimental Events	Experimental Total	Control Events	Control Total	RR	95%-CI	Weight (common)	Weight (random)
GRADUATE I	60	503	43	481	1.33	[0.92; 1.93]	6.5%	6.7%
GRADUATE II	64	501	50	474	1.21	[0.86; 1.72]	7.6%	7.6%
TRAILBLAZER-ALZ2	119	853	86	874	1.42	[1.09; 1.84]	12.5%	13.6%
Clarity_AD	100	898	73	897	1.37	[1.03; 1.82]	10.8%	11.2%
EMERGE	110	544	84	547	1.32	[1.02; 1.70]	12.3%	13.9%
ENGAGE	99	549	81	540	1.20	[0.92; 1.57]	12.0%	12.8%
EXPEDITION3	87	1054	88	1067	1.00	[0.75; 1.33]	12.9%	11.5%
EXPEDITION1 & 2	72	1027	77	1025	0.93	[0.68; 1.27]	11.3%	9.7%
Study 301	30	337	49	524	0.95	[0.62; 1.47]	5.6%	4.9%
Study 302	78	673	48	448	1.08	[0.77; 1.52]	8.5%	8.0%
**Common effect model**		**6939**		**6877**	**1.19**	**[1.08; 1.31]**	**100.0%**	**.**
**Random effects model**					**1.19**	**[1.08; 1.31]**	**.**	**100.0%**
**Prediction interval**						**[1.06; 1.34]**		

Heterogeneity: $I^2 = 0\%$, $\tau^2 = 0$, $p = 0.46$

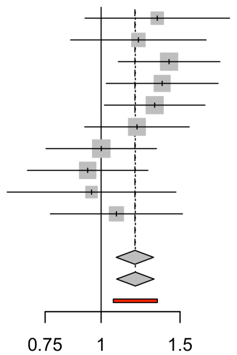

(b) Fall

Study	Experimental Events	Experimental Total	Control Events	Control Total	RR	95%-CI	Weight (common)	Weight (random)
GRADUATE I	63	503	62	481	0.97	[0.70; 1.35]	7.5%	7.4%
GRADUATE II	50	501	52	474	0.91	[0.63; 1.31]	6.4%	5.9%
TRAILBLAZER-ALZ2	114	853	110	874	1.06	[0.83; 1.36]	12.9%	13.3%
Clarity_AD	93	898	86	897	1.08	[0.82; 1.43]	10.2%	10.3%
EMERGE	68	544	71	547	0.96	[0.71; 1.31]	8.4%	8.2%
ENGAGE	80	549	57	540	1.38	[1.00; 1.90]	6.8%	7.9%
EXPEDITION3	162	1054	161	1067	1.02	[0.83; 1.25]	19.0%	19.7%
EXPEDITION1 & 2	92	1027	107	1025	0.86	[0.66; 1.12]	12.7%	11.3%
Study 301	43	337	73	524	0.92	[0.64; 1.30]	6.8%	6.5%
Study 302	100	673	64	448	1.04	[0.78; 1.39]	9.1%	9.5%
**Common effect model**		**6939**		**6877**	**1.01**	**[0.93; 1.11]**	**100.0%**	**.**
**Random effects model**					**1.01**	**[0.93; 1.11]**	**.**	**100.0%**
**Prediction interval**						**[0.91; 1.13]**		

Heterogeneity: $I^2 = 0\%$, $\tau^2 = 0$, $p = 0.71$

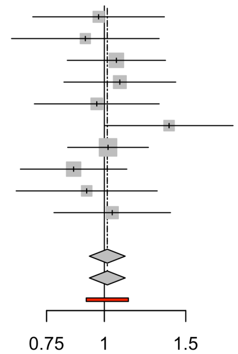

(c) Dizziness

Study	Experimental Events	Experimental Total	Control Events	Control Total	RR	95%-CI	Weight (common)	Weight (random)
GRADUATE I	45	503	41	481	1.05	[0.70; 1.57]	10.1%	10.3%
GRADUATE II	39	501	29	474	1.27	[0.80; 2.02]	7.2%	7.8%
TRAILBLAZER-ALZ2	53	853	48	874	1.13	[0.77; 1.65]	11.4%	11.7%
Clarity_AD	49	898	46	897	1.06	[0.72; 1.57]	11.1%	11.0%
EMERGE	42	544	44	547	0.96	[0.64; 1.44]	10.6%	10.2%
ENGAGE	49	549	54	540	0.89	[0.62; 1.29]	13.1%	12.4%
EXPEDITION3	62	1054	58	1067	1.08	[0.76; 1.53]	13.9%	13.9%
EXPEDITION1 & 2	58	1027	57	1025	1.02	[0.71; 1.45]	13.7%	13.3%
Study 302	54	673	31	448	1.16	[0.76; 1.77]	9.0%	9.3%
**Common effect model**		**6602**		**6353**	**1.06**	**[0.93; 1.20]**	**100.0%**	**.**
**Random effects model**					**1.06**	**[0.93; 1.20]**	**.**	**100.0%**
**Prediction interval**						**[0.90; 1.23]**		

Heterogeneity: $I^2 = 0\%$, $\tau^2 = 0$, $p = 0.98$

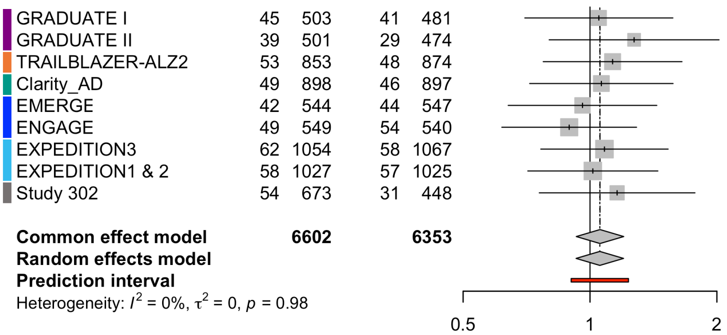

Gantenerumab **Donanemab** **Lecanemab** **Aducanumab** **Solanezumab** **Beprineuzumab**

S39 Figure: Forest plots for safety outcomes related to symptoms (low-dose populations).
