# Supplementary material for: Efficacy and safety of passive immunotherapies targeting amyloid beta in Alzheimer’s disease: A systematic review and meta-analysis
Source: PLoS Med. 2025 Mar 31;22(3):e1004568. doi: 10.1371/journal.pmed.1004568 (PMC12002640; doi:10.1371/journal.pmed.1004568)
Supplement: S40 Fig — (PDF) [file pmed.1004568.s041.pdf]

(a) Cerebral macrohemorrhage: low dose

Study	Experimental Events	Experimental Total	Control Events	Control Total	RR	95%-CI	Weight (common)	Weight (random)
GRADUATE I	4	481	8	503	0.52	[0.16; 1.73]	47.6%	27.9%
GRADUATE II	10	501	2	474	4.73	[1.04; 21.48]	12.5%	18.8%
TRAILBLAZER-ALZ2	3	853	2	874	1.54	[0.26; 9.18]	12.0%	14.0%
Clarity_AD	5	898	1	897	4.99	[0.58; 42.66]	6.1%	10.0%
Study 3000	0	267	0	344			0.0%	0.0%
Study 3001	5	439	2	439	2.50	[0.49; 12.82]	12.2%	16.4%
Study 300	1	337	0	524	4.66	[0.19; 114.11]	2.4%	4.7%
Study 301	2	673	1	448	1.33	[0.12; 14.64]	7.3%	8.1%
**Common effect model**		**4449**		**4503**	**1.84**	**[1.01; 3.35]**	**100.0%**	**--**
**Random effects model**					**1.78**	**[0.88; 3.61]**	**--**	**100.0%**
**Prediction interval**						**[0.53; 6.01]**		

Heterogeneity: $I^2 = 16\%$, $\tau^2 = 0.0936$, $p = 0.31$

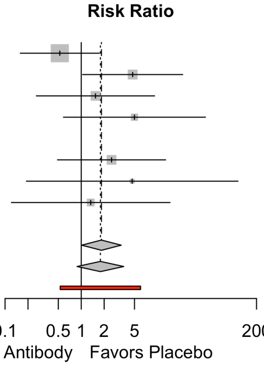

(b) Cerebral macrohemorrhage: high dose

Study	Experimental Events	Experimental Total	Control Events	Control Total	RR	95%-CI	Weight (common)	Weight (random)
GRADUATE I	4	481	8	503	0.52	[0.16; 1.73]	47.6%	27.9%
GRADUATE II	10	501	2	474	4.73	[1.04; 21.48]	12.5%	18.8%
TRAILBLAZER-ALZ2	3	853	2	874	1.54	[0.26; 9.18]	12.0%	14.0%
Clarity_AD	5	898	1	897	4.99	[0.58; 42.66]	6.1%	10.0%
Study 3000	0	263	0	344			0.0%	0.0%
Study 3001	5	439	2	439	2.50	[0.49; 12.82]	12.2%	16.4%
Study 300	1	329	0	524	4.78	[0.20; 116.88]	2.3%	4.7%
Study 301	2	673	1	448	1.33	[0.12; 14.64]	7.3%	8.2%
**Common effect model**		**4437**		**4503**	**1.84**	**[1.01; 3.35]**	**100.0%**	**--**
**Random effects model**					**1.79**	**[0.88; 3.62]**	**--**	**100.0%**
**Prediction interval**						**[0.53; 6.05]**		

Heterogeneity: $I^2 = 16\%$, $\tau^2 = 0.0953$, $p = 0.31$

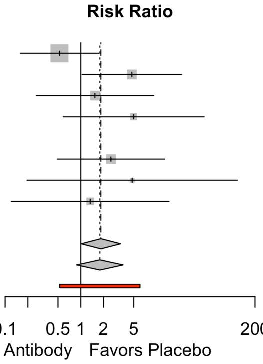

Gantenerumab **Donanemab** **Lecanemab** **Beprineuzumab**

S40 Figure: Forest plot for cerebral macrohemorrhage (low-dose and high-dose populations).
