# Supplementary material for: Efficacy and safety of passive immunotherapies targeting amyloid beta in Alzheimer’s disease: A systematic review and meta-analysis
Source: PLoS Med. 2025 Mar 31;22(3):e1004568. doi: 10.1371/journal.pmed.1004568 (PMC12002640; doi:10.1371/journal.pmed.1004568)
Supplement: S42 Fig — (a) Death, (b) Serious Adverse Event, (c) ARIA-E, (d) ARIA-H. ARIA-E, Amyloid-Related Imaging Abnormalities-Effusion, ARIA-H, Amyloid-Related Imaging Abnormalities-Hemorrhage. (PDF) [file pmed.1004568.s043.pdf]

## (a) Death

| Study | Experimental Events | Experimental Total | Control Events | Control Total | RR | 95%-CI | Weight (common) | Weight (random) |
|---|---|---|---|---|---|---|---|---|
| GRADUATE I | 3 | 503 | 10 | 481 | 0.29 | [0.08; 1.04] | 11.2% | 6.0% |
| GRADUATE II | 7 | 501 | 4 | 474 | 1.66 | [0.49; 5.62] | 4.5% | 6.5% |
| TRAILBLAZER-ALZ2 | 16 | 853 | 10 | 874 | 1.64 | [0.75; 3.59] | 10.8% | 13.5% |
| Clarity_AD | 6 | 898 | 7 | 897 | 0.86 | [0.29; 2.54] | 7.7% | 8.0% |
| EMERGI | 6 | 547 | 5 | 547 | 1.20 | [0.37; 3.91] | 5.5% | 6.9% |
| ENGAGI | 2 | 558 | 0 | 540 | 4.84 | [0.23; 100.56] | 0.6% | 1.2% |
| EXPEDITION3 | 9 | 1054 | 17 | 1067 | 0.54 | [0.24; 1.20] | 18.5% | 13.0% |
| EXPEDITION1 & 2 | 24 | 1027 | 19 | 1025 | 1.26 | [0.69; 2.29] | 20.8% | 19.8% |
| Study 3000 | 1 | 263 | 3 | 344 | 0.44 | [0.05; 4.17] | 2.8% | 2.1% |
| Study 3001 | 4 | 654 | 4 | 439 | 0.67 | [0.17; 2.67] | 5.2% | 5.2% |
| Study 301 | 7 | 329 | 7 | 524 | 1.59 | [0.56; 4.50] | 5.9% | 8.6% |
| Study 302 | 15 | 673 | 5 | 448 | 2.00 | [0.73; 5.46] | 6.6% | 9.1% |
| **Common effect model** | | **7860** | | **7660** | **1.08** | **[0.81; 1.43]** | **100.0%** | **.** |
| **Random effects model** | | | | | **1.08** | **[0.77; 1.50]** | **.** | **100.0%** |
| **Prediction interval** | | | | | | **[0.57; 2.04]** | | |

Heterogeneity: $I^2 = 16\%$, $\tau^2 = 0.0534$, $p = 0.29$

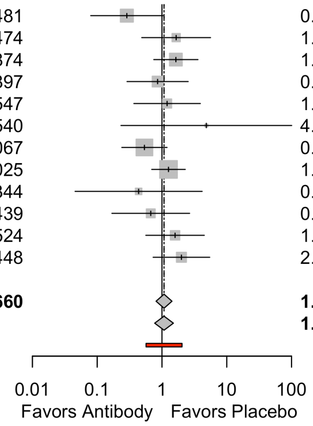


## (b) Serious Adverse Event

| Study | Experimental Events | Experimental Total | Control Events | Control Total | RR | 95%-CI | Weight (common) | Weight (random) |
|---|---|---|---|---|---|---|---|---|
| GRADUATE I | 76 | 503 | 95 | 481 | 0.77 | [0.58; 1.01] | 8.1% | 8.6% |
| GRADUATE II | 61 | 501 | 63 | 474 | 0.92 | [0.66; 1.27] | 5.4% | 6.8% |
| TRAILBLAZER-ALZ2 | 148 | 853 | 138 | 874 | 1.10 | [0.89; 1.36] | 11.3% | 11.4% |
| Clarity_AD | 126 | 898 | 101 | 897 | 1.25 | [0.98; 1.59] | 8.4% | 9.8% |
| EMERGE_ | 73 | 547 | 81 | 547 | 0.90 | [0.67; 1.21] | 6.7% | 7.9% |
| ENGAGE_nıgn | 79 | 558 | 70 | 540 | 1.09 | [0.81; 1.47] | 5.9% | 7.7% |
| EXPEDITION3 | 175 | 1054 | 202 | 1067 | 0.88 | [0.73; 1.05] | 16.7% | 12.9% |
| EXPEDITION1 & 2 | 261 | 1027 | 263 | 1025 | 0.99 | [0.85; 1.15] | 21.8% | 15.1% |
| Study 301 | 76 | 329 | 108 | 524 | 1.12 | [0.87; 1.45] | 6.9% | 9.2% |
| Study 302 | 176 | 673 | 89 | 448 | 1.32 | [1.05; 1.65] | 8.9% | 10.7% |
| **Common effect model** | | **6943** | | **6877** | **1.02** | **[0.95; 1.10]** | **100.0%** | **.** |
| **Random effects model** | | | | | **1.02** | **[0.92; 1.13]** | **.** | **100.0%** |
| **Prediction interval** | | | | | | **[0.77; 1.36]** | | |

Heterogeneity: $I^2 = 46\%$, $\tau^2 = 0.0126$, $p = 0.05$

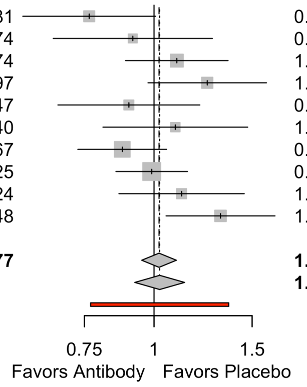


## (c) ARIA-E

| Study | Experimental Events | Experimental Total | Control Events | Control Total | RR | 95%-CI | Weight (common) | Weight (random) |
|---|---|---|---|---|---|---|---|---|
| GRADUATE I | 119 | 497 | 8 | 476 | 14.25 | [ 7.04; 28.83] | 7.5% | 9.6% |
| GRADUATE II | 128 | 496 | 18 | 470 | 6.74 | [ 4.18; 10.86] | 17.0% | 10.2% |
| TRAILBLAZER-ALZ2 | 205 | 853 | 18 | 874 | 11.67 | [ 7.28; 18.72] | 16.3% | 10.2% |
| Clarity_AD | 113 | 898 | 15 | 897 | 7.52 | [ 4.43; 12.79] | 13.8% | 10.1% |
| EMERGE | 188 | 541 | 13 | 544 | 14.54 | [ 8.40; 25.19] | 11.9% | 10.0% |
| ENGAGE_ | 199 | 554 | 16 | 532 | 11.94 | [ 7.28; 19.60] | 15.0% | 10.1% |
| EXPEDITION3 | 1 | 1054 | 2 | 1067 | 0.51 | [ 0.05; 5.57] | 1.8% | 4.3% |
| EXPEDITION1 & 2 | 9 | 1027 | 4 | 1025 | 2.25 | [ 0.69; 7.27] | 3.7% | 8.0% |
| Study 3000 | 31 | 263 | 2 | 344 | 20.27 | [ 4.90; 83.95] | 1.6% | 7.1% |
| Study 3001 | 109 | 654 | 9 | 439 | 8.13 | [ 4.16; 15.87] | 9.9% | 9.7% |
| Study 301 | 31 | 329 | 1 | 524 | 49.37 | [ 6.77; 359.95] | 0.7% | 5.3% |
| Study 302 | 103 | 448 | 1 | 673 | 154.73 | [21.67; 1104.90] | 0.7% | 5.4% |
| **Common effect model** | | **7614** | | **7865** | **11.39** | **[ 9.41; 13.79]** | **100.0%** | **.** |
| **Random effects model** | | | | | **10.53** | **[ 5.51; 20.11]** | **.** | **100.0%** |
| **Prediction interval** | | | | | | **[ 1.00; 111.18]** | | |

Heterogeneity: $I^2 = 64\%$, $\tau^2 = 1.0102$, $p < 0.01$

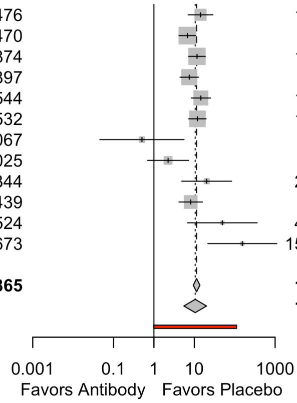


## (d) ARIA-H

| Study | Experimental Events | Experimental Total | Control Events | Control Total | RR | 95%-CI | Weight (common) | Weight (random) |
|---|---|---|---|---|---|---|---|---|
| GRADUATE I | 118 | 497 | 59 | 476 | 1.92 | [1.44; 2.55] | 13.5% | 14.5% |
| GRADUATE II | 109 | 496 | 57 | 470 | 1.81 | [1.35; 2.43] | 13.1% | 14.4% |
| TRAILBLAZER-ALZ2 | 268 | 853 | 119 | 874 | 2.31 | [1.90; 2.80] | 26.4% | 15.7% |
| Clarity_AD | 155 | 898 | 81 | 897 | 1.91 | [1.49; 2.46] | 18.2% | 15.0% |
| EMERGE | 108 | 541 | 37 | 544 | 2.94 | [2.06; 4.18] | 8.3% | 13.6% |
| ENGAGE | 109 | 554 | 34 | 532 | 3.08 | [2.13; 4.44] | 7.8% | 13.4% |
| EXPEDITION1&2 | 50 | 1027 | 57 | 1025 | 0.88 | [0.60; 1.27] | 12.8% | 13.4% |
| **Common effect model** | | **4866** | | **4818** | **2.05** | **[1.84; 2.27]** | **100.0%** | **--** |
| **Random effects model** | | | | | **1.99** | **[1.47; 2.69]** | **--** | **100.0%** |
| **Prediction interval** | | | | | | **[0.70; 5.64]** | | |

Heterogeneity: $I^2 = 81\%$, $\tau^2 = 0.1407$, $p < 0.01$

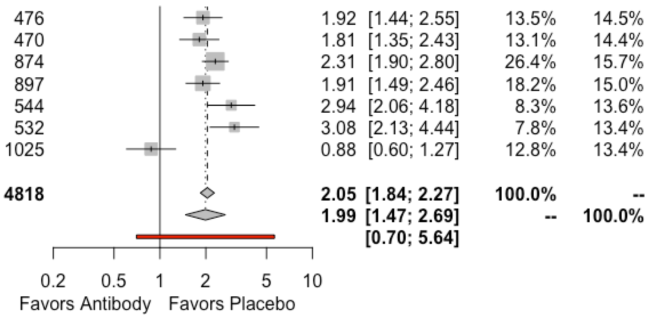


**Gantenerumab** **Donanemab** **Lecanemab** **Aducanumab** **Solanezumab** **Beprineuzumab**

S42 Figure: Forest plots for safety outcomes (high-dose populations).
