# Supplementary material for: Efficacy and safety of passive immunotherapies targeting amyloid beta in Alzheimer’s disease: A systematic review and meta-analysis
Source: PLoS Med. 2025 Mar 31;22(3):e1004568. doi: 10.1371/journal.pmed.1004568 (PMC12002640; doi:10.1371/journal.pmed.1004568)
Supplement: S43 Fig — (a) Headache, (b) Fall, and (c) Dizziness. (PDF) [file pmed.1004568.s044.pdf]

(a) Headache

Study	Experimental Events	Experimental Total	Control Events	Control Total	RR	95%-CI	Weight (common)	Weight (random)
GRADUATE I	60	503	43	481	1.33	[0.92; 1.93]	6.5%	6.6%
GRADUATE II	64	501	50	474	1.21	[0.86; 1.72]	7.6%	7.5%
TRAILBLAZER-ALZ2	119	853	86	874	1.42	[1.09; 1.84]	12.5%	13.4%
Clarity_AD	100	898	73	897	1.37	[1.03; 1.82]	10.7%	11.1%
EMERGE	107	547	84	547	1.27	[0.98; 1.65]	12.4%	13.5%
ENGAGE	115	558	81	540	1.37	[1.06; 1.78]	12.1%	13.7%
EXPEDITION3	87	1054	88	1067	1.00	[0.75; 1.33]	12.9%	11.3%
EXPEDITION1 & 2	72	1027	77	1025	0.93	[0.68; 1.27]	11.3%	9.5%
Study 301	34	329	49	524	1.11	[0.73; 1.67]	5.6%	5.3%
Study 302	78	673	48	448	1.08	[0.77; 1.52]	8.5%	7.9%
**Common effect model**		**6943**		**6877**	**1.21**	**[1.10; 1.34]**	**100.0%**	.
**Random effects model**					**1.22**	**[1.11; 1.34]**	.	**100.0%**
**Prediction interval**						**[1.09; 1.36]**		

Heterogeneity: $I^2 = 0\%$, $\tau^2 = 0$, $p = 0.49$

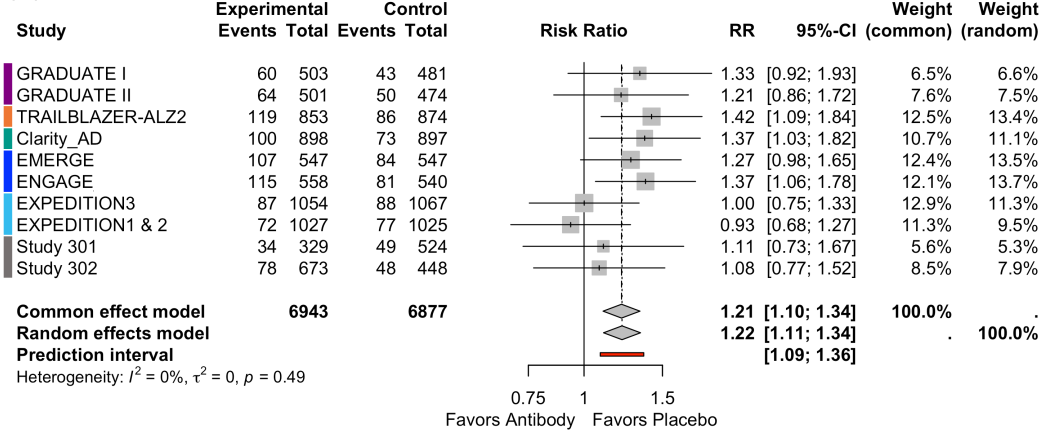

(b) Fall

Study	Experimental Events	Experimental Total	Control Events	Control Total	RR	95%-CI	Weight (common)	Weight (random)
GRADUATE I	63	503	62	481	0.97	[0.70; 1.35]	7.5%	7.4%
GRADUATE II	50	501	52	474	0.91	[0.63; 1.31]	6.4%	5.8%
TRAILBLAZER-ALZ2	114	853	110	874	1.06	[0.83; 1.36]	12.9%	13.2%
Clarity_AD	93	898	86	897	1.08	[0.82; 1.43]	10.2%	10.2%
EMERGE	76	547	71	547	1.07	[0.79; 1.45]	8.4%	8.7%
ENGAGE	86	558	57	540	1.46	[1.07; 2.00]	6.9%	8.0%
EXPEDITION3	162	1054	161	1067	1.02	[0.83; 1.25]	19.0%	19.6%
EXPEDITION1 & 2	92	1027	107	1025	0.86	[0.66; 1.12]	12.7%	11.2%
Study 301	43	329	73	524	0.94	[0.66; 1.33]	6.7%	6.4%
Study 302	100	673	64	448	1.04	[0.78; 1.39]	9.1%	9.4%
**Common effect model**		**6943**		**6877**	**1.03**	**[0.94; 1.13]**	**100.0%**	.
**Random effects model**					**1.03**	**[0.94; 1.13]**	.	**100.0%**
**Prediction interval**						**[0.93; 1.14]**		

Heterogeneity: $I^2 = 0\%$, $\tau^2 = 0$, $p = 0.57$

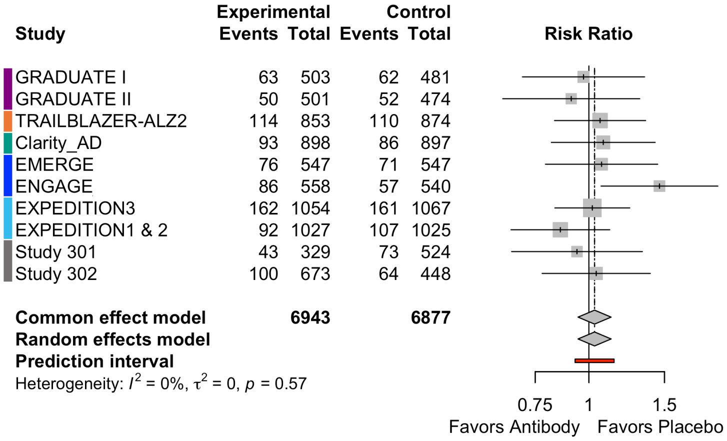

(c) Dizziness

Study	Experimental Events	Experimental Total	Control Events	Control Total	RR	95%-CI	Weight (common)	Weight (random)
GRADUATE I	45	503	41	481	1.05	[0.70; 1.57]	10.1%	10.1%
GRADUATE II	39	501	29	474	1.27	[0.80; 2.02]	7.2%	7.6%
TRAILBLAZER-ALZ2	53	853	48	874	1.13	[0.77; 1.65]	11.4%	11.5%
Clarity_AD	49	898	46	897	1.06	[0.72; 1.57]	11.1%	10.7%
EMERGE	55	547	44	547	1.25	[0.86; 1.82]	10.6%	11.5%
ENGAGE	54	558	54	540	0.97	[0.68; 1.38]	13.2%	12.8%
EXPEDITION3	62	1054	58	1067	1.08	[0.76; 1.53]	13.9%	13.6%
EXPEDITION1 & 2	58	1027	57	1025	1.02	[0.71; 1.45]	13.7%	13.0%
Study 302	54	673	31	448	1.16	[0.76; 1.77]	8.9%	9.1%
**Common effect model**		**6614**		**6353**	**1.10**	**[0.96; 1.25]**	**100.0%**	.
**Random effects model**					**1.10**	**[0.96; 1.25]**	.	**100.0%**
**Prediction interval**						**[0.94; 1.28]**		

Heterogeneity: $I^2 = 0\%$, $\tau^2 = 0$, $p = 0.99$

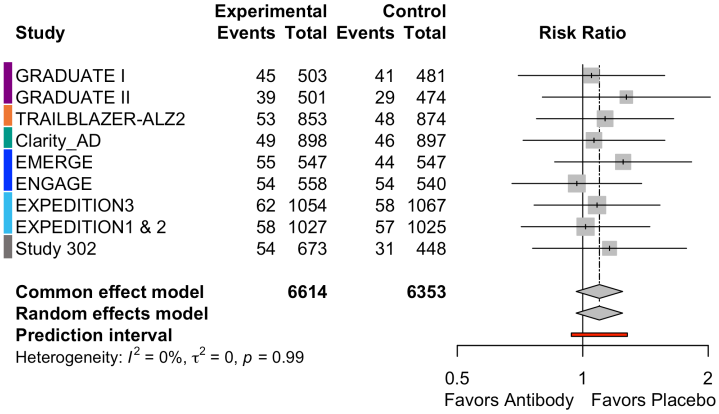

Gantenerumab | Donanemab | Lecanemab | Aducanumab | Solanezumab | Beprineuzumab

S43 Figure: Forest plots for safety outcomes related to symptoms (high-dose populations).
