# Supplementary material for: Efficacy and safety of passive immunotherapies targeting amyloid beta in Alzheimer’s disease: A systematic review and meta-analysis
Source: PLoS Med. 2025 Mar 31;22(3):e1004568. doi: 10.1371/journal.pmed.1004568 (PMC12002640; doi:10.1371/journal.pmed.1004568)
Supplement: S45 Fig — (a) Death, (b) Serious adverse event, (c) ARIA-E, (d) ARIA-H. Filled circles represent estimated treatment effect (risk ratio) and its precision (standard error) for each individual study. In addition to individual study results, the fixed effect estimate (vertical dashed line) with 95% confidence interval limits (diagonal dashed lines) and the random effects estimate (vertical dotted line) are shown in the figures. Also, p-values of Egger’s test are shown. *P-value < 0.05. ARIA-E, Amyloid-Related Imaging Abnormalities-Effusion, ARIA-H, Amyloid-Related Imaging Abnormalities-Hemorrhage. (PDF) [file pmed.1004568.s046.pdf]

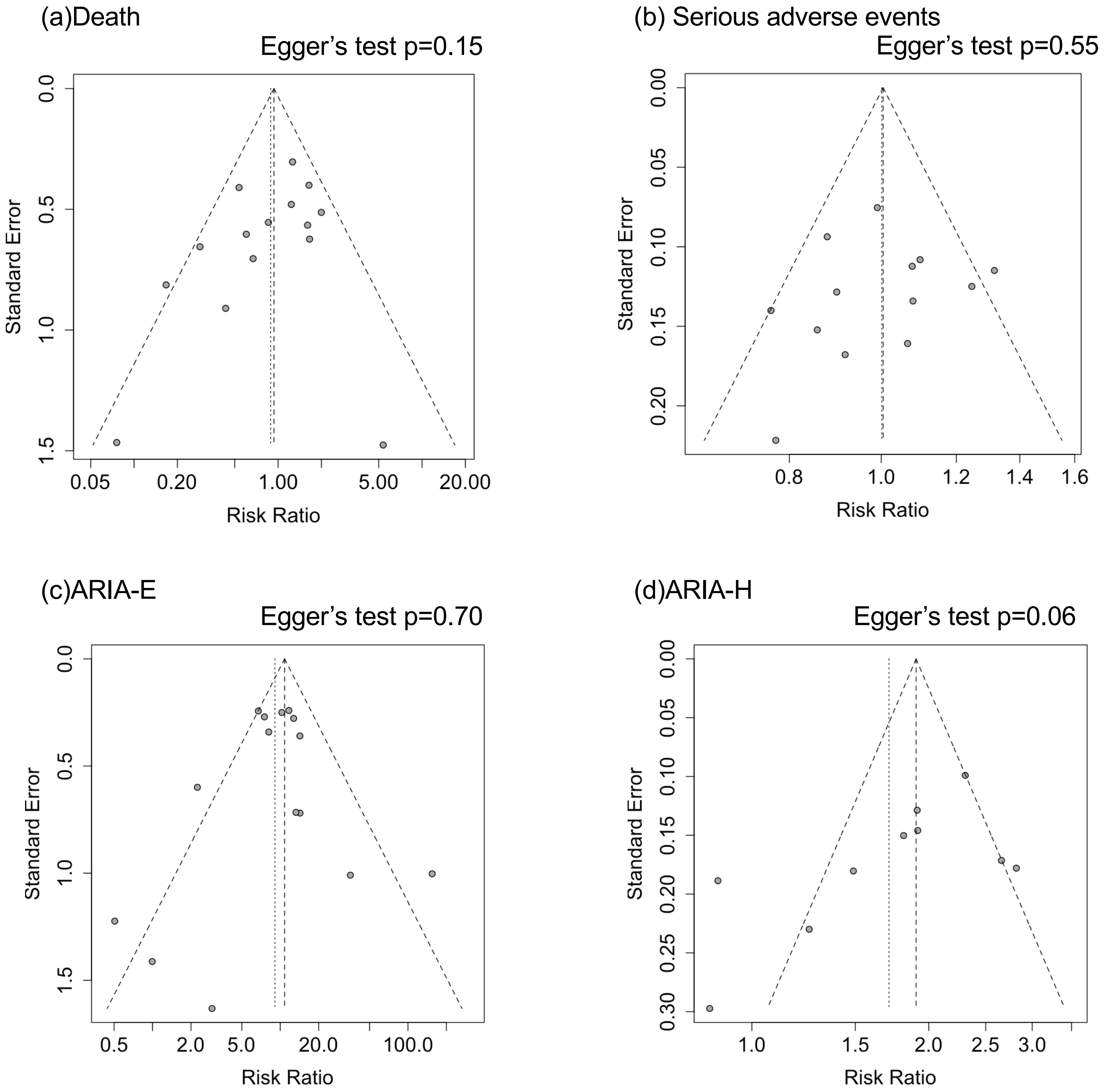

S45 Figure: Funnel plots for safety endpoints in sensitivity analysis 8 (including halted trials with a sample size of fewer than 200 patients in each arm).
