# Supplementary material for: Efficacy and safety of passive immunotherapies targeting amyloid beta in Alzheimer’s disease: A systematic review and meta-analysis
Source: PLoS Med. 2025 Mar 31;22(3):e1004568. doi: 10.1371/journal.pmed.1004568 (PMC12002640; doi:10.1371/journal.pmed.1004568)
Supplement: S47 Fig — Forest plots for the change in Clinical Dementia Rating-Sum of Boxes (CDR-SB). (PDF) [file pmed.1004568.s048.pdf]

CDR-SB

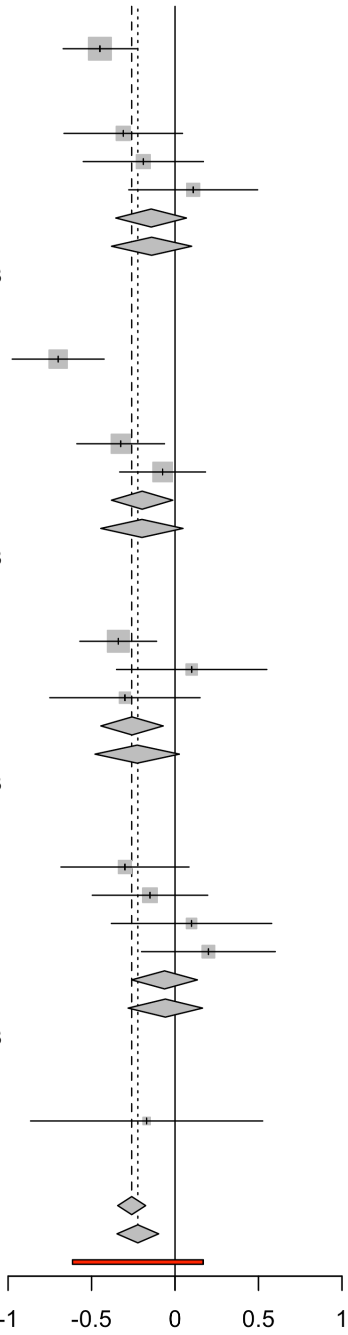

Study	MD	SE(MD)	MD	95%-CI	Weight (common)	Weight (random)
**Lecanemab**						
Clarity_AD	-0.4500	0.1122	-0.45	[-0.67; -0.23]	14.5%	9.8%
**Gantenerumab**						
GRADUATE I	-0.3100	0.1811	-0.31	[-0.66; 0.04]	5.6%	6.6%
GRADUATE II	-0.1900	0.1837	-0.19	[-0.55; 0.17]	5.4%	6.5%
Scarlet_RoAD	0.1088	0.1969	0.11	[-0.28; 0.49]	4.7%	6.0%
**Common effect model**			**-0.14**	**[-0.35; 0.07]**	**15.7%**	**.**
**Random effects model**			**-0.14**	**[-0.38; 0.10]**	**.**	**19.1%**
Heterogeneity: $I^2 = 22\%$, $\tau^2 = 0.0100$, $p = 0.28$						
**Donanemab**						
TRAILBLAZER-ALZ2	-0.7000	0.1403	-0.70	[-0.97; -0.43]	9.3%	8.4%
**Aducanumab**						
EMERGE	-0.3252	0.1340	-0.33	[-0.59; -0.06]	10.2%	8.7%
ENGAGE	-0.0742	0.1311	-0.07	[-0.33; 0.18]	10.6%	8.8%
**Common effect model**			**-0.20**	**[-0.38; -0.01]**	**20.8%**	**.**
**Random effects model**			**-0.20**	**[-0.44; 0.05]**	**.**	**17.5%**
Heterogeneity: $I^2 = 44\%$, $\tau^2 = 0.0139$, $p = 0.18$						
**Solanezumab**						
EXPEDITION3	-0.3400	0.1173	-0.34	[-0.57; -0.11]	13.3%	9.5%
EXPEDITION1	0.1000	0.2296	0.10	[-0.35; 0.55]	3.5%	5.0%
EXPEDITION2	-0.3000	0.2296	-0.30	[-0.75; 0.15]	3.5%	5.0%
**Common effect model**			**-0.26**	**[-0.44; -0.07]**	**20.2%**	**.**
**Random effects model**			**-0.23**	**[-0.48; 0.03]**	**.**	**19.5%**
Heterogeneity: $I^2 = 32\%$, $\tau^2 = 0.0177$, $p = 0.23$						
**Bapineuzumab**						
Study 3000	-0.3000	0.1956	-0.30	[-0.68; 0.08]	4.8%	6.1%
Study 3001	-0.1500	0.1764	-0.15	[-0.50; 0.20]	5.9%	6.8%
Study 301	0.0989	0.2449	0.10	[-0.38; 0.58]	3.0%	4.6%
Study 302	0.2000	0.2041	0.20	[-0.20; 0.60]	4.4%	5.8%
**Common effect model**			**-0.06**	**[-0.26; 0.13]**	**18.1%**	**.**
**Random effects model**			**-0.06**	**[-0.28; 0.17]**	**.**	**23.1%**
Heterogeneity: $I^2 = 21\%$, $\tau^2 = 0.0110$, $p = 0.28$						
**Crenezumab**						
CREAD	-0.1700	0.3546	-0.17	[-0.86; 0.52]	1.5%	2.6%
CREAD2	.	.			0.0%	0.0%
**Common effect model**			**-0.26**	**[-0.34; -0.18]**	**100.0%**	**.**
**Random effects model**			**-0.22**	**[-0.35; -0.10]**	**.**	**100.0%**
**Prediction interval**				**[-0.61; 0.17]**		

Heterogeneity: $I^2 = 52\%$, $\tau^2 = 0.0288$, $p < 0.01$

Test for subgroup differences (common effect): $\chi^2_6 = 18.24$, df = 6 ($p < 0.01$)

Test for subgroup differences (random effects): $\chi^2_6 = 17.05$, df = 6 ($p < 0.01$)

S47 Figure: Sensitivity analysis 8 (including halted trials with a sample size of fewer than 200 patients in each arm). Forest plots for the change in CDR-SB.
