# Supplementary material for: Efficacy and safety of passive immunotherapies targeting amyloid beta in Alzheimer’s disease: A systematic review and meta-analysis
Source: PLoS Med. 2025 Mar 31;22(3):e1004568. doi: 10.1371/journal.pmed.1004568 (PMC12002640; doi:10.1371/journal.pmed.1004568)
Supplement: S48 Fig — Forest plots for the change in Alzheimer’s Disease Assessment Scale-Cognitive Subscale (ADAS-Cog). (PDF) [file pmed.1004568.s049.pdf]

ADAS-Cog

Study	SMD	SE(SMD)	SMD	95%-CI	Weight (common)	Weight (random)
**Lecanemab**						
Clarity_AD	-0.1686	0.0496	-0.17	[-0.27; -0.07]	8.5%	8.5%
**Gantenerumab**						
GRADUATE I	-0.1081	0.0560	-0.11	[-0.22; 0.00]	6.6%	6.6%
GRADUATE II	-0.1114	0.0504	-0.11	[-0.21; -0.01]	8.2%	8.2%
Scarlet_RoAD	-0.0663	0.1175	-0.07	[-0.30; 0.16]	1.5%	1.5%
**Common effect model**			**-0.11**	**[-0.18; -0.04]**	**16.3%**	**.**
**Random effects model**			**-0.11**	**[-0.18; -0.04]**	**.**	**16.3%**
Heterogeneity: $I^2 = 0\%$, $\tau^2 = 0$, $p = 0.94$						
**Donanemab**						
TRAILBLAZER-ALZ2	-0.1360	0.0403	-0.14	[-0.22; -0.06]	12.8%	12.8%
**Aducanumab**						
EMERGE	-0.1936	0.0737	-0.19	[-0.34; -0.05]	3.8%	3.8%
ENGAGE	-0.1145	0.0744	-0.11	[-0.26; 0.03]	3.8%	3.8%
**Common effect model**			**-0.15**	**[-0.26; -0.05]**	**7.6%**	**.**
**Random effects model**			**-0.15**	**[-0.26; -0.05]**	**.**	**7.6%**
Heterogeneity: $I^2 = 0\%$, $\tau^2 = 0$, $p = 0.45$						
**Solanezumab**						
EXPEDITION3	-0.0681	0.0406	-0.07	[-0.15; 0.01]	12.6%	12.6%
EXPEDITION1	-0.0553	0.0459	-0.06	[-0.15; 0.03]	9.9%	9.9%
EXPEDITION2	-0.0821	0.0451	-0.08	[-0.17; 0.01]	10.2%	10.2%
**Common effect model**			**-0.07**	**[-0.12; -0.02]**	**32.7%**	**.**
**Random effects model**			**-0.07**	**[-0.12; -0.02]**	**.**	**32.7%**
Heterogeneity: $I^2 = 0\%$, $\tau^2 = 0$, $p = 0.92$						
**Bapineuzumab**						
Study 3000	-0.0751	0.0707	-0.08	[-0.21; 0.06]	4.2%	4.2%
Study 3001	-0.0034	0.0618	-0.00	[-0.12; 0.12]	5.4%	5.4%
Study 301	0.0043	0.0606	0.00	[-0.11; 0.12]	5.7%	5.7%
Study 302	-0.0194	0.0593	-0.02	[-0.14; 0.10]	5.9%	5.9%
**Common effect model**			**-0.02**	**[-0.08; 0.04]**	**21.1%**	**.**
**Random effects model**			**-0.02**	**[-0.08; 0.04]**	**.**	**21.1%**
Heterogeneity: $I^2 = 0\%$, $\tau^2 = 0$, $p = 0.84$						
**Crenezumab**						
CREAD	-0.0339	0.1415	-0.03	[-0.31; 0.24]	1.0%	1.0%
CREAD2	.	.			0.0%	0.0%
**Common effect model**			**-0.09**	**[-0.12; -0.06]**	**100.0%**	**.**
**Random effects model**			**-0.09**	**[-0.12; -0.06]**	**.**	**100.0%**
**Prediction interval**				**[-0.12; -0.06]**		

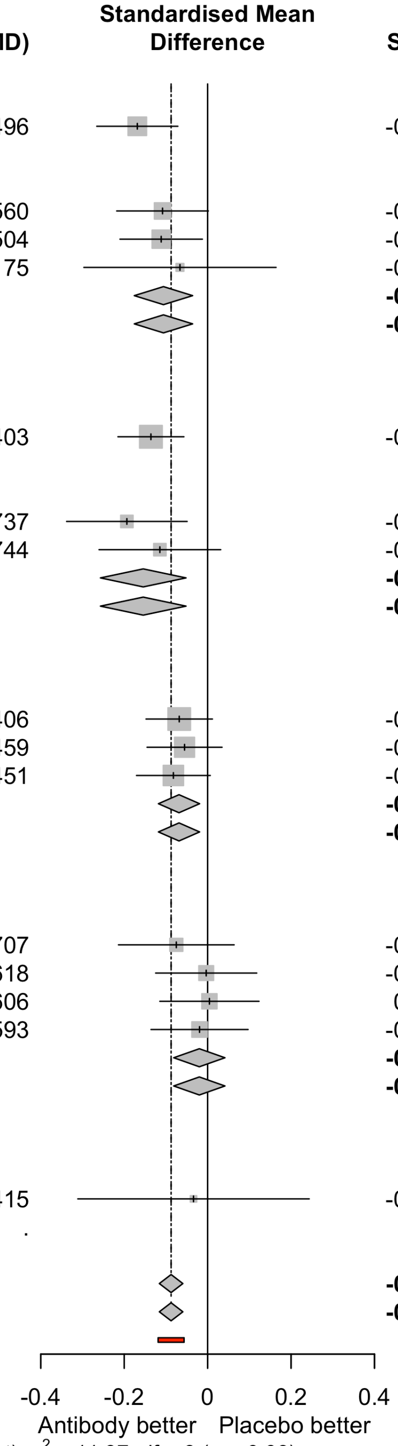

Heterogeneity: $I^2 = 0\%$, $\tau^2 = 0$, $p = 0.52$
Test for subgroup differences (common effect): $\chi^2_6 = 11.37$, df = 6 ($p = 0.08$)
Test for subgroup differences (random effects): $\chi^2_6 = 11.37$, df = 6 ($p = 0.08$)

S48 Figure: Sensitivity analysis 8 (including halted trials with a sample size of fewer than 200 patients in each arm). Forest plots for the change in ADAS-Cog.
