# Supplementary material for: Efficacy and safety of passive immunotherapies targeting amyloid beta in Alzheimer’s disease: A systematic review and meta-analysis
Source: PLoS Med. 2025 Mar 31;22(3):e1004568. doi: 10.1371/journal.pmed.1004568 (PMC12002640; doi:10.1371/journal.pmed.1004568)
Supplement: S49 Fig — (a) Death, (b) Serious adverse event. (PDF) [file pmed.1004568.s050.pdf]

(a)Death

Study	Experimental Events	Experimental Total	Control Events	Control Total	RR	95%-CI	Weight (common)	Weight (random)
GRADUATE I	3	503	10	481	0.29	[0.08; 1.04]	8.8%	6.1%
GRADUATE II	7	501	4	474	1.66	[0.49; 5.62]	3.5%	6.5%
TRAILBLAZER-ALZ2	16	853	10	874	1.64	[0.75; 3.59]	8.5%	10.0%
Clarity_AD	6	898	7	897	0.86	[0.29; 2.54]	6.0%	7.4%
EMERGE	6	1091	5	547	0.60	[0.18; 1.96]	5.7%	6.8%
ENGAGE	5	1107	0	540	5.37	[0.30; 96.91]	0.6%	1.7%
EXPEDITION3	9	1054	17	1067	0.54	[0.24; 1.20]	14.5%	9.8%
EXPEDITION1 & 2	24	1027	19	1025	1.26	[0.69; 2.29]	16.4%	11.9%
Study 3000	2	530	3	344	0.43	[0.07; 2.58]	3.1%	3.9%
Study 3001	4	654	4	439	0.67	[0.17; 2.67]	4.1%	5.6%
Study 301	11	666	7	524	1.24	[0.48; 3.17]	6.7%	8.6%
Study 302	15	673	5	448	2.00	[0.73; 5.46]	5.2%	8.1%
CREAD	8	404	5	405	1.60	[0.53; 4.86]	4.3%	7.3%
CREAD2	0	404	6	398	0.08	[0.00; 1.34]	5.6%	1.8%
Scarlet_RoAD	2	531	6	266	0.17	[0.03; 0.82]	6.9%	4.6%
**Common effect**		**10896**		**8729**	**0.94**	**[0.72; 1.21]**	**100.0%**	**.**
**Random effects model**					**0.89**	**[0.59; 1.33]**	**.**	**100.0%**
**Prediction interval**						**[0.27; 2.94]**		

Heterogeneity: $I^2 = 38\%$, $\tau^2 = 0.2639$, $p = 0.07$

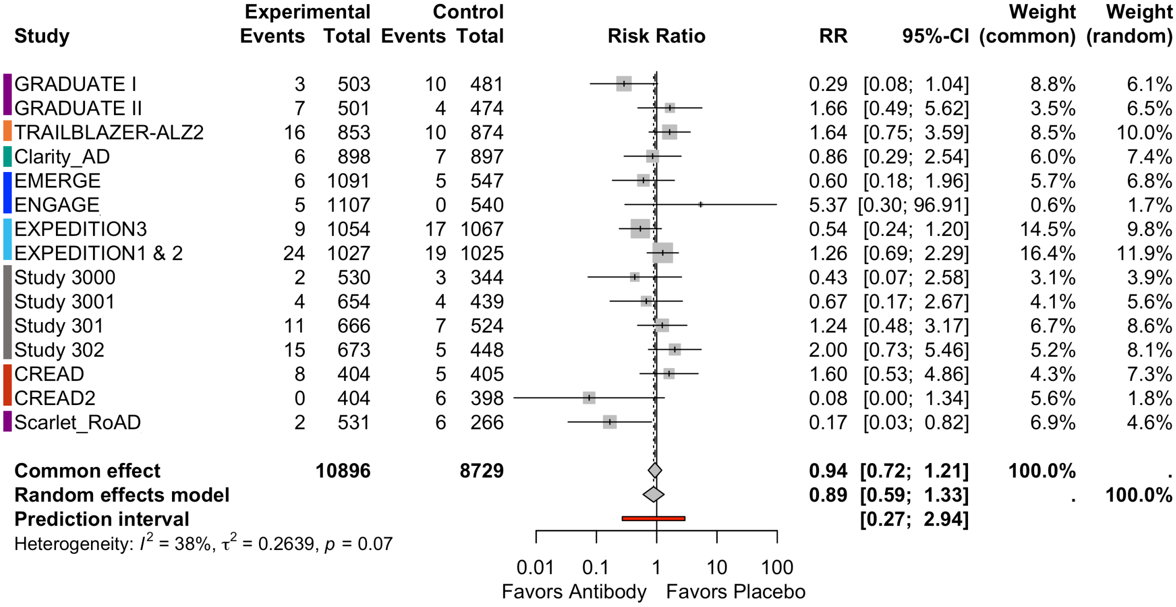

(b) Serious adverse events

Study	Experimental Events	Experimental Total	Control Events	Control Total	RR	95%-CI	Weight (common)	Weight (random)
GRADUATE I	76	503	95	481	0.77	[0.58; 1.01]	6.6%	6.8%
GRADUATE II	61	501	63	474	0.92	[0.66; 1.27]	4.4%	5.3%
TRAILBLAZER-ALZ2	148	853	138	874	1.10	[0.89; 1.36]	9.3%	9.3%
Clarity_AD	126	898	101	897	1.25	[0.98; 1.59]	6.9%	7.8%
EMERGE	145	1091	81	547	0.90	[0.70; 1.15]	7.3%	7.6%
ENGAGE	155	1107	70	540	1.08	[0.83; 1.40]	6.4%	7.2%
EXPEDITION3	175	1054	202	1067	0.88	[0.73; 1.05]	13.6%	10.7%
EXPEDITION1 & 2	261	1027	263	1025	0.99	[0.85; 1.15]	17.9%	12.8%
Study 301	148	666	108	524	1.08	[0.87; 1.34]	8.2%	8.9%
Study 302	176	673	89	448	1.32	[1.05; 1.65]	7.3%	8.7%
CREAD	67	404	63	405	1.07	[0.78; 1.46]	4.3%	5.6%
CREAD2	33	404	42	398	0.77	[0.50; 1.20]	2.9%	3.4%
Scarlet_RoAD	94	531	55	266	0.86	[0.64; 1.15]	5.0%	6.0%
**Common effect**		**9712**		**7946**	**1.00**	**[0.94; 1.07]**	**100.0%**	**.**
**Random effects model**					**1.00**	**[0.92; 1.09]**	**.**	**100.0%**
**Prediction interval**						**[0.79; 1.27]**		

Heterogeneity: $I^2 = 38\%$, $\tau^2 = 0.0099$, $p = 0.08$

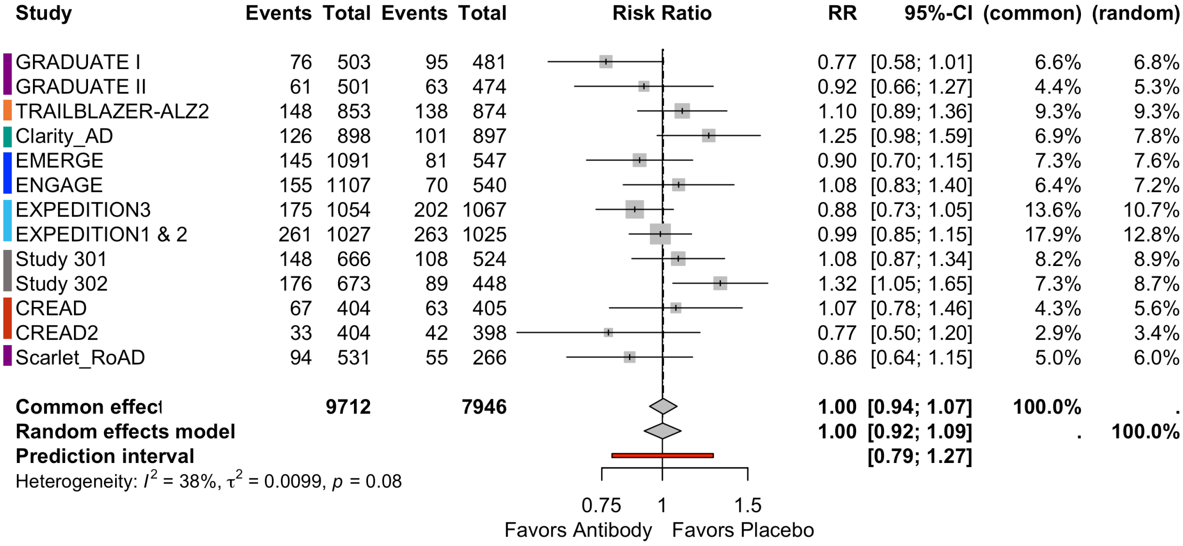

Gantenerumab **Donanemab** **Lecanemab** **Aducanumab** **Solanezumab** **Beprineuzumab** **Crenezumab**

S49 Figure: Sensitivity analysis 8 (including halted trials with a sample size of fewer than 200 patients in each arm). Forest plots for safety outcomes.
