# Supplementary material for: Efficacy and safety of passive immunotherapies targeting amyloid beta in Alzheimer’s disease: A systematic review and meta-analysis
Source: PLoS Med. 2025 Mar 31;22(3):e1004568. doi: 10.1371/journal.pmed.1004568 (PMC12002640; doi:10.1371/journal.pmed.1004568)
Supplement: S50 Fig — (a)ARIA-E, (b) ARIA-H. ARIA-E, Amyloid-Related Imaging Abnormalities-Effusion; ARIA-H, Amyloid-Related Imaging Abnormalities-Hemorrhage. (PDF) [file pmed.1004568.s051.pdf]

(a)ARIA-E

Study	Experimental Events	Experimental Total	Control Events	Control Total	RR	95%-CI	Weight (common)	Weight (random)
GRADUATE I	119	497	8	476	14.25	[7.04; 28.83]	6.6%	8.6%
GRADUATE II	128	496	18	470	6.74	[4.18; 10.86]	15.0%	9.4%
TRAILBLAZER-ALZ2	205	853	18	874	11.67	[7.28; 18.72]	14.4%	9.4%
Clarity_AD	113	898	15	897	7.52	[4.43; 12.79]	12.1%	9.2%
EMERGE	328	1078	13	544	12.73	[7.39; 21.95]	14.0%	9.2%
ENGAGE	340	1099	16	532	10.29	[6.30; 16.80]	17.4%	9.3%
EXPEDITION3	1	1054	2	1067	0.51	[0.05; 5.57]	1.6%	3.4%
EXPEDITION1 & 2	9	1027	4	1025	2.25	[0.69; 7.27]	3.2%	6.9%
Study 3000	44	530	2	344	14.28	[3.48; 58.52]	2.0%	6.0%
Study 3001	109	654	9	439	8.13	[4.16; 15.87]	8.7%	8.8%
Study 301	45	666	1	524	35.41	[4.90; 256.00]	0.9%	4.3%
Study 302	103	448	1	673	154.73	[21.67; 1104.90]	0.6%	4.4%
CREAD	1	399	1	397	0.99	[0.06; 15.85]	0.8%	2.8%
CREAD2	1	398	0	388	2.92	[0.12; 71.58]	0.4%	2.3%
Scarlet_RoAD	53	531	2	266	13.27	[3.26; 54.05]	2.2%	6.1%
**Common effect model**		**10628**		**8916**	**10.82**	**[8.94; 13.08]**	**100.0%**	**.**
**Random effects model**					**9.09**	**[5.26; 15.69]**	**.**	**100.0%**
**Prediction interval**						**[1.24; 66.39]**		

Heterogeneity: $I^2 = 54\%$, $\tau^2 = 0.7697$, $p < 0.01$

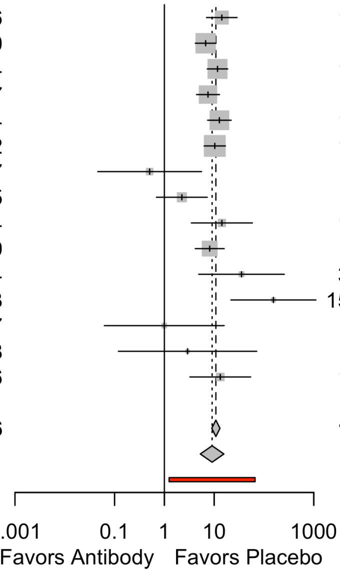

(b)ARIA-H

Study	Experimental Events	Experimental Total	Control Events	Control Total	RR	95%-CI	Weight (common)	Weight (random)
GRADUATE I	118	497	59	476	1.92	[1.44; 2.55]	10.6%	10.7%
GRADUATE II	109	496	57	470	1.81	[1.35; 2.43]	10.3%	10.6%
TRAILBLAZER-ALZ2	268	853	119	874	2.31	[1.90; 2.80]	20.6%	11.5%
Clarity_AD	155	898	81	897	1.91	[1.49; 2.46]	14.2%	11.0%
EMERGE	195	1078	37	544	2.66	[1.90; 3.72]	8.6%	10.1%
ENGAGE	198	1099	34	532	2.82	[1.99; 4.00]	8.0%	10.0%
EXPEDITION1 & 2	50	1027	57	1025	0.88	[0.60; 1.27]	10.0%	9.8%
CREAD	39	399	31	397	1.25	[0.80; 1.96]	5.4%	8.9%
CREAD2	20	398	23	388	0.85	[0.47; 1.52]	4.1%	7.5%
Scarlet_RoAD	104	531	35	266	1.49	[1.05; 2.12]	8.2%	10.0%
**Common effect model**		**7276**		**5869**	**1.90**	**[1.73; 2.10]**	**100.0%**	**.**
**Random effects model**					**1.71**	**[1.33; 2.20]**	**.**	**100.0%**
**Prediction interval**						**[0.70; 4.19]**		

Heterogeneity: $I^2 = 79\%$, $\tau^2 = 0.1341$, $p < 0.01$

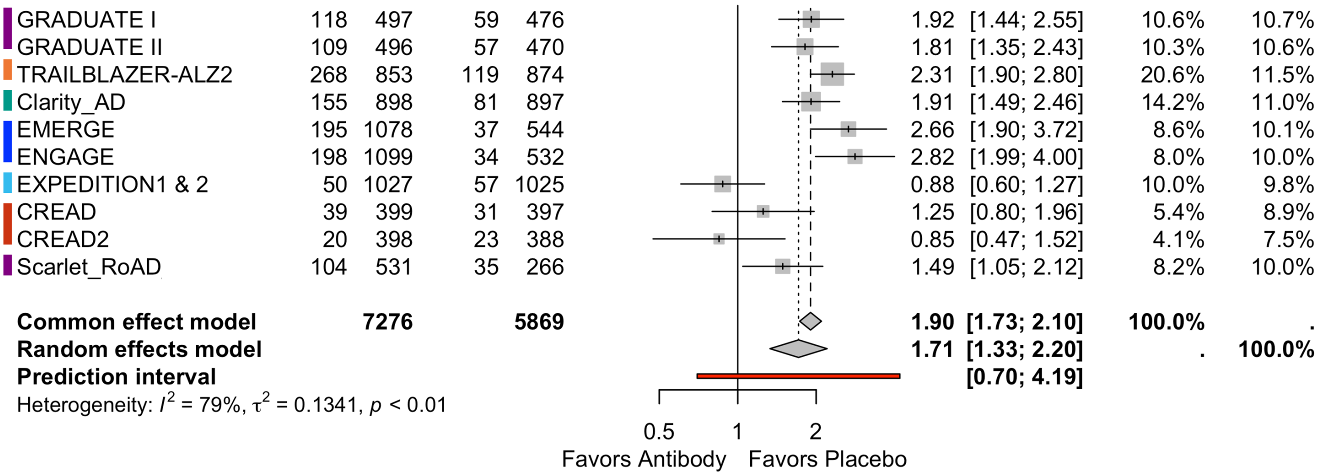

Gantenerumab **Donanemab** **Lecanemab** **Aducanumab** **Solanezumab** **Beprineuzumab** **Crenezumab**

S50 Figure: Sensitivity analysis 8 (including halted trials with a sample size of fewer than 200 patients in each arm). Forest plots for safety outcomes related to imaging abnormalities.
