# Supplementary material for: Efficacy and safety of passive immunotherapies targeting amyloid beta in Alzheimer’s disease: A systematic review and meta-analysis
Source: PLoS Med. 2025 Mar 31;22(3):e1004568. doi: 10.1371/journal.pmed.1004568 (PMC12002640; doi:10.1371/journal.pmed.1004568)
Supplement: S51 Fig — (a) Headache, (b) Fall, (c) Dizziness. (PDF) [file pmed.1004568.s052.pdf]

(a)Headache

Study	Experimental Events	Experimental Total	Control Events	Control Total	RR	95%-CI	Weight (common)	Weight (random)
GRADUATE I	60	503	43	481	1.33	[0.92; 1.93]	5.1%	5.8%
GRADUATE II	64	501	50	474	1.21	[0.86; 1.72]	5.9%	6.5%
TRAILBLAZER-ALZ2	119	853	86	874	1.42	[1.09; 1.84]	9.8%	10.5%
Clarity_AD	100	898	73	897	1.37	[1.03; 1.82]	8.4%	9.0%
EMERGE_high	217	1091	84	547	1.30	[1.03; 1.63]	12.9%	12.7%
ENGAGE_high	214	1107	81	540	1.29	[1.02; 1.63]	12.6%	12.3%
EXPEDITION3	87	1054	88	1067	1.00	[0.75; 1.33]	10.1%	9.1%
EXPEDITION1 & 2	72	1027	77	1025	0.93	[0.68; 1.27]	8.9%	7.9%
Study 301_high	64	666	49	524	1.03	[0.72; 1.46]	6.3%	6.3%
Study 302	78	673	48	448	1.08	[0.77; 1.52]	6.7%	6.8%
CREAD	39	404	45	405	0.87	[0.58; 1.30]	5.2%	4.9%
CREAD2	25	404	22	398	1.12	[0.64; 1.95]	2.6%	2.8%
Scarlet_RoAD_high	59	531	36	266	0.82	[0.56; 1.21]	5.5%	5.4%
**Common effect model**		**9712**		**7946**	**1.16**	**[1.07; 1.27]**	**100.0%**	**.**
**Random effects model**					**1.16**	**[1.05; 1.27]**	**.**	**100.0%**
**Prediction interval**						**[0.96; 1.40]**		

Heterogeneity: $I^2 = 17\%$, $\tau^2 = 0.0049$, $p = 0.28$

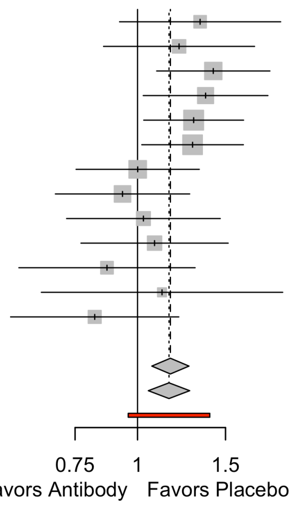

(b)Fall

Study	Experimental Events	Experimental Total	Control Events	Control Total	RR	95%-CI	Weight (common)	Weight (random)
GRADUATE I	63	503	62	481	0.97	[0.70; 1.35]	6.3%	6.2%
GRADUATE II	50	501	52	474	0.91	[0.63; 1.31]	5.3%	5.0%
TRAILBLAZER-ALZ2	114	853	110	874	1.06	[0.83; 1.36]	10.8%	11.2%
Clarity_AD	93	898	86	897	1.08	[0.82; 1.43]	8.6%	8.6%
EMERGE_high	144	1091	71	547	1.02	[0.78; 1.33]	9.4%	9.5%
ENGAGE_high	166	1107	57	540	1.42	[1.07; 1.88]	7.6%	8.4%
EXPEDITION3	162	1054	161	1067	1.02	[0.83; 1.25]	16.0%	16.6%
EXPEDITION1 & 2	92	1027	107	1025	0.86	[0.66; 1.12]	10.7%	9.5%
Study 301_high	86	666	73	524	0.93	[0.69; 1.24]	8.1%	7.9%
Study 302	100	673	64	448	1.04	[0.78; 1.39]	7.7%	7.9%
CREAD	43	404	33	405	1.31	[0.85; 2.01]	3.3%	3.6%
CREAD2	20	404	24	398	0.82	[0.46; 1.46]	2.4%	2.0%
Scarlet_RoAD_high	51	531	28	266	0.91	[0.59; 1.41]	3.7%	3.5%
**Common effect model**		**9712**		**7946**	**1.03**	**[0.95; 1.12]**	**100.0%**	**.**
**Random effects model**					**1.03**	**[0.95; 1.11]**	**.**	**100.0%**
**Prediction interval**						**[0.94; 1.12]**		

Heterogeneity: $I^2 = 0\%$, $\tau^2 = 0$, $p = 0.61$

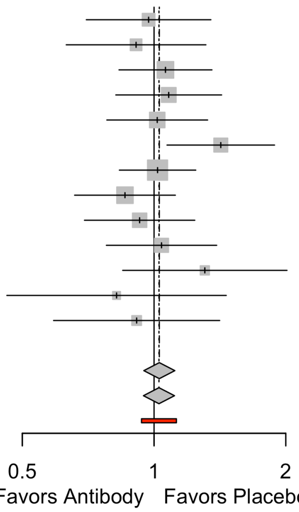

(c)Dizziness

Study	Experimental Events	Experimental Total	Control Events	Control Total	RR	95%-CI	Weight (common)	Weight (random)
GRADUATE I	45	503	41	481	1.05	[0.70; 1.57]	8.2%	8.3%
GRADUATE II	39	501	29	474	1.27	[0.80; 2.02]	5.8%	6.3%
TRAILBLAZER-ALZ2	53	853	48	874	1.13	[0.77; 1.65]	9.2%	9.4%
Clarity_AD	49	898	46	897	1.06	[0.72; 1.57]	9.0%	8.8%
EMERGE_high	97	1091	44	547	1.11	[0.79; 1.55]	11.4%	11.6%
ENGAGE_high	103	1107	54	540	0.93	[0.68; 1.27]	14.1%	13.8%
EXPEDITION3	62	1054	58	1067	1.08	[0.76; 1.53]	11.2%	11.2%
EXPEDITION1 & 2	58	1027	57	1025	1.02	[0.71; 1.45]	11.1%	10.7%
Study 302	54	673	31	448	1.16	[0.76; 1.77]	7.3%	7.5%
CREAD	23	404	27	405	0.85	[0.50; 1.46]	5.3%	4.6%
CREAD2	19	404	10	398	1.87	[0.88; 3.97]	2.0%	2.4%
Scarlet_RoAD_high	48	531	21	266	1.15	[0.70; 1.87]	5.5%	5.6%
**Common effect model**		**9046**		**7422**	**1.08**	**[0.96; 1.21]**	**100.0%**	**.**
**Random effects model**					**1.08**	**[0.96; 1.21]**	**.**	**100.0%**
**Prediction interval**						**[0.94; 1.23]**		

Heterogeneity: $I^2 = 0\%$, $\tau^2 = 0$, $p = 0.95$

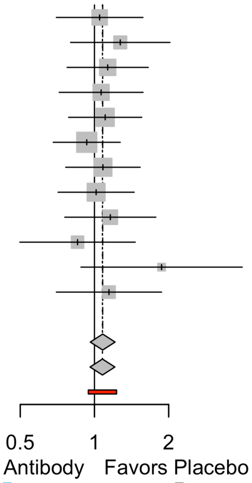

Gantenerumab | Donanemab | Lecanemab | Aducanumab | Solanezumab | Beprineuzumab | Crenezumab

S51 Figure: Sensitivity analysis 8 (including halted trials with a sample size of fewer than 200 patients in each arm). Forest plots for safety outcomes related to symptoms.
