# Supplementary material for: Efficacy and safety of passive immunotherapies targeting amyloid beta in Alzheimer’s disease: A systematic review and meta-analysis
Source: PLoS Med. 2025 Mar 31;22(3):e1004568. doi: 10.1371/journal.pmed.1004568 (PMC12002640; doi:10.1371/journal.pmed.1004568)
Supplement: Attachment — Submitted filename: Updated_ResponseLetter_R4_Feixiong.Cheng.pdf [file pmed.1004568.s056.pdf]

Point-by-Point Response Letter

Manuscript #: PMEDICINE-D-24-02295R2

Responses to the Reviewers and editors:
We would like to thank the reviewers for their constructive critique, which has helped us to improve the manuscript. Please find below our point-by point responses to the reviewer's comments.

Following the reviewers advise and the journal's policy, we thoroughly reviewed the manuscript, and additionally provided 2 files in: https://github.com/ChengF-Lab/Meta_ADAb

- s_meta_ab_antibody_data_additional.csv -Includes trial-level data collected from trials, including those halted due to futility that ended with fewer than 200 patients in each arm.
- s_meta_ab_antibody_TextF.R. - R script used in the comparason between meta and metafor packages (TextF in S1 Supporting information)

Also, we archived it in Zenodo (https://zenodo.org/records/14502672).

Also, a total of five references were added in response to the comments, and reference numbers were updated accordingly. Please note that changes made did not influence the content, conclusions, or framework of the paper. We have not listed below all minor changes made; however, these are highlighted in red in the revised manuscript.

Response to the editorial team

Editorial team Comment 1-1

Please note that we require a point-by-point response to not only reviewer comments, but all editorial comments, including general editorial requests. Please be sure to provide such a document that addresses the editorial comments of the previous decision letter.

Author Response

Thank you for pointing this out. We provide the point-by-point response to the previous editorial comments below. Also, during the second revision, we double checked we follow general editorial requests. Blue texts are our response.

-- ---
General editorial requests:
(Note: not all will apply to your paper, but please check each item carefully)

* We ask every co-author listed on the manuscript to fill in a contributing author statement, making sure to declare all competing interests. If any of the co-authors have not filled in the statement, we will remind them to do so when the paper is revised. If all statements are not completed in a timely fashion this could hold up the re-review process. If new competing interests are declared later in the revision process, this may also hold up the submission. Should there be a problem getting one of your co-authors to fill in a statement we will be in contact. Please do not add or remove authors without first discussing this with the handling editor. You can see our competing interests policy here: http://journals.plos.org/plosmedicine/s/competing-interests.

- Response: We understood the instructions. With regard to the authorship, I apologize for the oversight in the initial submission. Would it be possible to add Dr. Noah Lorincz-Comi at this stage?

* Please upload any figures associated with your paper as individual TIF or EPS files with 300dpi resolution at resubmission; please read our figure guidelines for more information on our requirements: http://journals.plos.org/plosmedicine/s/figures. While revising your submission, please upload your figure files to the PACE digital diagnostic tool, https://pacev2.apexcovantage.com/. PACE helps ensure that figures meet PLOS requirements. To use PACE, you must first register as a user. Then, login and navigate to the UPLOAD tab, where you will find detailed instructions on how to use the tool. If you encounter any issues or have any questions when using PACE, please email us at PLOSMedicine@plos.org.

    - Response: Thank you for the reminder. We uploaded all the figures as individual TIF files. Please let us know if further modification is needed.

* Please ensure that the paper adheres to the PLOS Data Availability Policy (see http://journals.plos.org/plosmedicine/s/data-availability), which requires that all data underlying the study's findings be provided in a repository or as Supporting Information. For data residing with a third party, authors are required to provide instructions with contact information (web or email address) for obtaining the data. Please note that a study author cannot be the contact person for the data. PLOS journals do not allow statements supported by "data not shown" or "unpublished results." For such statements, authors must provide supporting data or cite public sources that include it.

    - Response: At this time, we uploaded the data and the R script to our github. We provided the information in the data availability statement.

Search results, data collected from each study and the R script used for the analysis are available on Github (https://github.com/ChengF-Lab/Meta_ADAb) and archived in ZENODO(https://zenodo.org/records/14502672).

* We expect all researchers with submissions to PLOS in which author-generated code underpins the findings in the manuscript to make all author-generated code available without restrictions upon publication of the work. In cases where code is central to the manuscript, we may require the code to be made available as a condition of publication. Authors are responsible for ensuring that the code is reusable and well documented. Please make any custom code available, either as part of your data deposition or as a supplementary file. Please add a sentence to your data availability statement regarding any code used in the study, e.g. "The code used in the analysis is available from Github [URL] and archived in Zenodo [DOI link]" Please review our guidelines at https://journals.plos.org/plosmedicine/s/materials-software-and-code-sharing and ensure that your code is shared in a way that follows best practice and facilitates reproducibility and reuse. Because Github depositions can be readily changed or deleted, we encourage you to make a permanent DOI'd copy (e.g. in Zenodo) and provide the URL.

    - Response: We uploaded the data and R script on Github and archived in Zenodo. We revised the data availability statement accordingly.

“Search results, data collected from each study and the R script used for the analysis are available on Github (https://github.com/ChengF-Lab/Meta_ADAb) and archived in ZENODO(https://zenodo.org/records/14502672).”

* FINANCIAL DISCLOSURES: Please state whether any sponsors or funders (other than the named authors) played any role in study design, data collection and analysis, the decision to publish, or preparation of the manuscript. If they had no role in the research, include this sentence: “The funders had no role in study design, data collection and analysis, decision to publish, or preparation of the manuscript.”

- Response: Thank you for the reminder. We updated the financial disclosure statement in the manuscript and the author center.

FORMATTING - GENERAL
* Abstract: Please structure your abstract using the PLOS Medicine headings (Background, Methods and Findings, Conclusions). Please combine the Methods and Findings sections into one section.

- Response: Thank you for the reminder. We updated the structure of the abstract as suggested.

* At this stage, we ask that you include a short, non-technical Author Summary of your research to make findings accessible to a wide audience that includes both scientists and non-scientists. The Author Summary should immediately follow the Abstract in your revised manuscript. This text is subject to editorial change and should be distinct from the scientific abstract. Ideally each sub-heading should contain 2-3 single sentence, concise bullet points containing the most salient points from your study. In the final bullet point of 'What Do These Findings Mean?', please include the main limitations of the study in non-technical language. Please see our author guidelines for more information: https://journals.plos.org/plosmedicine/s/revising-your-manuscript#loc-author-summary.

- Response: Thank you for the instruction. We added the author summary after the abstract.

* Please express the main results with 95% CIs as well as p values. When reporting p values please report as p<0.001 and where higher as the exact p value p=0.002, for example. Throughout, suggest reporting statistical information as follows to improve clarity for the reader "22% (95% CI [13%,28%]; p</=)". Please be sure to define all numerical values at first use.

- Response: Thank you for the reminder. We showed the results appropriately.

  * Please include page numbers and line numbers in the manuscript file. Use continuous line numbers (do not restart the numbering on each page).
- Response: We added the page numbers and line numbers in the manuscript file.

* Please cite the reference numbers in square brackets. Citations should precede punctuation.

- Response: Thank you for the instruction. We modified the reference numbers.

FIGURES AND TABLES
* Please provide titles and legends for all figures and tables (including those in Supporting Information files).

- Response: We provided the titles and legends for all figures (and supplemental figures) at the end of the manuscript. Tables were embedded in the manuscript where each table was mentioned for the first time.

*Please define all abbreviations used in each figure/table (including those in Supporting Information files).

- Response: We defined all abbreviations in each figure/ tables.

* Please consider avoiding the use of red and green in order to make your figure more accessible to those with color blindness.

- Response: We did not use red and green in order.

SUPPLEMENTARY MATERIAL

* Please note that supplementary material will be posted as supplied by the authors. Therefore, please amend it according to the relevant comments outlined here.

- Response: We understood.

* Please cite your Supporting Information as outlined here: https://journals.plos.org/plosmedicine/s/supporting-information

- Response: We changed the citation format accordingly. We uploaded the Figures S1-Figures S51, and S1 Supporting information. In the S1 Supporting information, there are Appendix A, Table A-G, and Text A-H. Please let us know if we need to rename or anything.

REFERENCES

* PLOS uses the numbered citation (citation-sequence) method and first six authors, et al.

- Response: We confirmed that the citation format follows the PLOS guideline.

* Please ensure that journal name abbreviations match those found in the National Center for Biotechnology Information (NCBI) databases (http://www.ncbi.nlm.nih.gov/nlmcatalog/journals), and are appropriately formatted and capitalised.

- Response: We confirmed that the journal names match those found in the NCBI databases.

* Where website addresses are cited, please include the complete URL and specify the date of access (e.g. [accessed: 12/06/2024]).

- Response: Where website addresses are cited, we specified the date of access.

* Please also see https://journals.plos.org/plosmedicine/s/submission-guidelines#loc-references for further details on reference formatting.

- Response: Thank you for the reference. We checked the guidelines and confirmed that we followed the guideline appropriately.

STUDY TYPE-SPECIFIC REQUESTS

* Please report your SR/MA according to the PRISMA guidelines provided at the EQUATOR site. http://www.equator-network.org/reporting-guidelines/prisma/. Please provide the completed PRISMA checklist as Supporting Information. When completing the checklist, please use section and paragraph numbers, rather than page numbers. Please add the following statement, or similar, to the Methods: "This study is reported as per the Preferred Reporting Items for Systematic Reviews and Meta-Analyses (PRISMA) guideline (S1 Checklist)."

- Response: It is in Appendix A in S1 Supporting information. Also mentioned in the manuscript. (Line 102)
  "Preferred Reporting Items for Systemic Reviews and Meta-Analyses (PRISMA) guidelines were followed (**Appendix A in S1 Supporting information**)."

* Abstract: Please report your abstract according to PRISMA for abstracts (https://doi.org/10.1371/journal.pmed.1001419) following the PLOS Medicine abstract structure (Background, Methods and Findings, Conclusions). Please ensure you provide dates of search, data sources, number of studies included, types of study designs included, eligibility criteria, and synthesis/appraisal methods.

- We confirm that the above-mentioned information is covered in the abstract.

* Please note that we expect searches to be updated to within 6 months of the time of submission.

- Thank you for the reminder. Our original search was conducted on January 14th 2024. We understand that the first submission date was just a few days before July 18th 2024, which is nearly 6 months after the search date. We apologize for the timing being close to the 6-month limit. To the best of our knowledge, no new randomized controlled trials relevant to this meta-analysis have been published since the search date. We are prepared to update the search results at this time if the editors or reviewers require it. Please let us know if an update is needed.

Responses to the Reviewer #2:

**Reviewer Comment 2-1**
It would be helpful to provide a clearer statement on the assumptions underlying the meta-regression, particularly regarding potential inter-study variability in moderator definitions.

**Author Response**
Thank you for pointing this out. We added clearer statements on the assumptions underlying the meta-regression, and described the potential inter-study variability in detail.
**Revision**
Following sentence were added in the method section.<br><br>Page 8, Line 154

"While we attempted to standardize the definitions of moderators as much as possible, there were inherent differences in antibody characteristics and patient profiles across studies. For example, for AD stage, among the trials classified as "early", EXPEDITION 3, which tested Solanezumab, only included patients with mild AD but not MCI [16]. Regarding binding mechanisms, Solanezumab was classified as monomer-targeting, Donanemab as plaque-specific, while oligomer- or aggregate-binding antibodies exhibit varying affinities to fibrils, oligomers, and monomers [2,4,16-18]. Furthermore, baseline Aβ burden in the brain, measured by amyloid PET and reported in centiloids, was available only in substudy populations within a limited subset of trials. Consequently, meta-regression analyses for baseline Aβ burden were conducted using these substudies."

Page10, Line 195
"The meta-regression was performed under the assumption that the modifiers had a linear relationship with the outcome and that the heterogeneity observed could be partly explained by these modifiers."

Following reference was added to describe the variability in antibody affinity.
18. Linse S, Scheidt T, Bernfur K et.al., Kinetic fingerprints differentiate the mechanisms of action of anti-Ab antibodies. Nat Struct Mol Biol. 2020;27:1125-1133.

Reviewer Comment 2-2

While the "meta" package was chosen for the analysis, the manuscript does not explain why this specific package was selected over other options, such as "metafor," which may offer alternative estimators or variance structures for meta-regression. Please provide a rationale for selecting the "meta" package, possibly including a brief comparison to other methods or tools, to ensure that this choice yields the most reliable results for the data used.

Author Response

Thank you for pointing this out. We selected the "meta" package for our analysis because of its straightforward implementation, compatibility with our dataset structure, and robust tools for conducting both meta-analysis and meta-regression. Both the "meta" and "metafor" packages are widely used and validated tools (Viechtbauer W et.al., J Stat Soft 2010). Following your advice, we performed comparative analyses for selected examples using the metafor package and showed the results in **Text F in S1 Supporting information.**

We provided the R script for this comparative analysis in github (https://github.com/ChengF-Lab/Meta_ADAb, R script name: s_meta_ab_antibody_Text.R).
It was archived in ZENODO. https://zenodo.org/records/14502672

In summary, outputs were identical or similar between meta and metafor outputs, except for some difference in I^2 values. Then, we additionally performed meta-analyses in primary and secondary endpoints using metafor package, and summarized the I^2 values below.

	Meta	Metafor
**Primary endpoints**		
CDR-SB	53.10	53.56
ADAS-Cog	7.00	7.79
**Secondary endpoints**		

Death	21.20	22.43
SAE	46.30	49.02
ARIA-E	59.90	87.34
ARIA-H	78.80	84.92
Headache	0	0
Fall	0	0
Dizziness	0	0

The difference in output is derived from the difference in calculation methods (Detailed in the **Text F in supporting information**). Both methods are described in the original publication where I2 was introduced by Higgins et.al., in 2002 (Higgins JPT et.al., Statist Med 2002, Reference #19).

Using the criteria Higgins JPT et.al., proposed in the same paper, we judged the heterogeneity mild if I^2 is less than 30%, moderate if I^2 is 30-50%, and substantial if I^2 is >50%. Using this criteria, there was no discrepancy in interpretation of heterogeneity based on the output from Meta and Metafor packages.
Please also refer to the response to the comment No.7.

Revision

We added **Text F in S1 Supporting information** to show the results of comparative analyses for selected samples using the metafor package**.**

We added the following sentences in the methods section (page 10, Line 207).
"To ensure the consistency of results, we also performed comparative analyses for selected examples using the metafor package version 4.6-0 [2], focusing on both categorical and continuous outcomes, as well as meta-regression. Representative case studies are presented, and detailed comparisons are provided in **Text F in S1 Supporting information**."

We added the following reference.
19. Higgins JPT, Thompson SG. Quantifying heterogeneity in a meta-analysis. Statist Med. 2002; 21:1539-1558.
29. Schwarzar G, Carpenter JR, Rucker G. Meta-Analysis with R. Springer 2015. ISBN : 978-3-319-21415-3 doi:10.1007/978-3-319-21416-0
30. Viechtbauer W. Conducting Meta-Analyses in R with the metafor Package. J. Stat. Soft. 2010;36(3):1-48.
31. The metafor Package: I2 for Multilevel and Multivariate Models.[Available from: https://www.metafor-project.org/doku.php/tips%3Ai2_multilevel_multivariate] Accessed December 13th, 2024.

We provided the R script for this comparative analysis in github (https://github.com/ChengF-Lab/Meta_ADAb, R script name: s_meta_ab_antibody_Text.R).
It was archived in ZENODO. https://zenodo.org/records/14502672

Reviewer Comment 2-3

Egger's test and funnel plots are utilized to assess publication bias for certain outcomes, but it remains unclear how selective reporting was addressed, particularly regarding smaller trials with fewer than 200 participants, which were excluded. It would be valuable to discuss any

potential impact of excluding these smaller studies on publication bias and to describe any steps taken to mitigate this bias.

Author Response

Thank you for pointing the important point. We added a sensitivity analysis including smaller trials with fewer than 200 participants in each arm. Effect size for CDR-SB was slightly small, but other than that, the results were consistent with the main analysis. Even when three trials with fewer than 200 participants per group were included, the Egger's test P-value for CDR-SB change remained <0.05, as these trials were terminated early due to lack of efficacy. Please also refer to the response to the comment No. 4.

Revision

We added a sensitivity analysis including smaller trials with fewer than 200 participants in each arm, and showed the results in **S47-S51 Figures.** Funnel plots are shown in **S47-S49 Figures.** For detail, please refer to the response to the comment #4.

We added the following sentence in the main results. (Page 32, Line 446)
"Importantly, sensitivity analysis, including smaller studies with fewer than 200 participants per arm, demonstrated consistent findings in ADAS-Cog, confirming the robustness of our results."

We added the following sentence in the discussion. (page 31, Line 422)
"Notably, even when three trials with fewer than 200 participants per group were included, the Egger's test P-value for CDR-SB change remained <0.05, as these trials were terminated early due to lack of efficacy [34,35]."

Reviewer Comment 2-4

Trials like those for Aducanumab were excluded based on interim or incomplete data. However, excluding these trials entirely may overlook potentially valuable emerging evidence. Please discuss any impact this exclusion may have on the conclusions. Alternatively, consider including a separate analysis that cautiously incorporates these interim data to examine their influence on the pooled estimates.

Author Response

Actually, trials that tested Aducanumab (EMERGE and ENGAGE) were included in the main analysis since the total number of patients were >200 in each arm in both efficacy and safety outcomes, even after they were halted as a result of the interim analysis. We showed the results of sensitivity analyses excluding these trials in **S28-30 Figures**. After excluding these trials, the resultant effect sizes for primary secondary endpoints were similar to the main analysis, and it was already mentioned in the result section (page 28, Line 348).

Other RCTs that were excluded after ending the number of patients in each arm<200 were two trials of Crenezumab (Ostrowitzki S et.al., JAMA Neurology 2022, Ref No.34) and one trial (with 2 doses) of Gantenerumab in prodromal Alzheimer's disease (Ostrowizki S et.al., Alzheimers Res Ther 2017, Ref No.35). We performed an additional analysis including these trials.

Effect size for CDR-SB was slightly small, but other than that, the results were consistent with the main analysis. For efficacy, heterogeneity in results for change in CDR-SB is substantial, and we think ADAS-Cog results are more reliable. When efficacy was evaluated using ADAS-Cog, the effect size was similar to the main analysis.

We provided the data additionally corrected for this analysis in our Github.
https://github.com/ChengF-Lab/Meta_ADAb
File name:
s_meta_ab_antibody_data_additional.csv

Please also refer to the response to the comment No. 3.

Revision

In the method section, we added the following sentence. (Page 10, Line 203)
"Finally, we included trials previously excluded due to having fewer than 200 participants per arm, to assess their impact on the robustness of the findings."

In the result section, we added the following sentences (Page 29, Line 367).
"Finally, we included trials excluded in the main analysis due to having fewer than 200 participants per arm, to assess their impact on the robustness of the findings [34,35]. All three trials were halted early due to futility. Two trials tested Crenezumab but the efficacy endpoint results were available only in CREAD and not in CREAD2 [34]. Funnel plots and P-values for Egger's test for primary and secondary endpoints are shown in S44-S46 Figures. Egger's test P-values were 0.04 for CDR-SB. Similar to the main analysis, trials with small sample size that favored antibody therapy were potentially missing in CDR-SB. There was no evidence of asymmetry in funnel plots in secondary endpoints. The resultant effect sizes for primary endpoints were slightly smaller than the main analyses in CDR-SB, and similar in ADAS-Cog (CDR-SB: MD=0.22 [-0.35, -0.10]; ADAS-Cog: SMD=-0.09 [-0.12, -0.06]) (S47–S48 Figures). Results for secondary endpoints were similar to the main analysis (S49–S51 Figures)."

We modified the Table 5, and added the above-mentioned sensitivity analysis results.

We added **S44-S51 Figures** and their legends.

We added the following sentence in the main results. (Page 32, Line 446)
"Importantly, sensitivity analysis, including smaller studies with fewer than 200 participants per arm, demonstrated consistent findings in ADAS-Cog, confirming the robustness of our results."

We added the following sentence in the discussion. (page 31, Line 422)
"Notably, even when three trials with fewer than 200 participants per group were included, the Egger's test P-value for CDR-SB change remained <0.05, as these trials were terminated early due to lack of efficacy [34,35]."

We provided the data additionally corrected for this analysis in our Github.
https://github.com/ChengF-Lab/Meta_ADAb
File name:
s_meta_ab_antibody_data_additional.csv

Reviewer Comment 2-5

The manuscript does not provide details on how missing data were managed within the meta-regression analysis. Given that some trials reported different outcomes or utilized distinct scales (e.g., ADAS-Cog versions 11, 13, and 14), this could introduce inconsistencies in the results if not addressed adequately. Please describe any imputation methods or sensitivity analyses conducted for missing data. If missing data were not specifically addressed, consider including this as a limitation, as it could impact the meta-regression's robustness.

**Author Response**
Thank you for raising this important point. Indeed, ADAS-Cog scores were reported using different versions (11, 13, and 14) across the included trials. To address this, we calculated the standardized mean difference (SMD) as described in **Supplemental Text D**, and this served as the basis for conducting the meta-analysis.<br><br>Regarding the additional items in ADAS-Cog 13 and 14 that are not present in ADAS-Cog 11, we did not impute these data. This decision was based on the assumption that the scores for items unique to ADAS-Cog 13 and 14 are correlated with the scores from items shared across all versions. We acknowledge this as a limitation, and mentioned in the limitation section.<br><br>Furthermore, some complications were only reported in a subset of trials, and these have been summarized in Table A. No imputation was performed for these complications either, given their limited reporting.
**Revision**
We modified the following sentences in the limitation section (page 36, Line 520).<br>"First, ADAS-Cog scores were reported using different versions (11, 13, and 14), and SMD were calculated for standardization. However, no imputation was performed for unique items in ADAS-Cog 13 and 14, relying on the assumption that these items correlate with shared items across versions, which is a limitation. Reports on secondary or tertiary endpoints were also unavailable in some RCTs, and no imputation was performed for these."

**Reviewer Comment 2-6**
There are some inconsistencies in format and reporting of confidence intervals and p-values. Please review the manuscript for consistency in reporting statistical significance and confidence intervals (e.g., using either 95% CI consistently throughout).

**Author Response**
We thoroughly reviewed the manuscript and modified the following points.<br>In the manuscript text and tables, results were shown with Value (95%CI).<br>example:<br>1.00 (0.80,2.00)<br>-1.25 (-0.80,2.65)<br><br>Please also refer to the response to the comment No.9.
**Revision**

We thoroughly reviewed the paper and modified the reporting formats.

Reviewer Comment 2-7

Although heterogeneity is assessed and reported, the manuscript could benefit from a more detailed discussion of what specific levels of heterogeneity imply for the interpretation of results in this context. Consider adding a brief section discussing the interpretation of heterogeneity levels (e.g., low, moderate, high) and their potential influence on the reliability of pooled estimates.

Author Response

Thank you for the supportive comment. For the cutoff for the I^2 values to judge the interpretation of heterogeneity levels, we set the cutoffs based on the publication where I^2 score was originally introduced (Higgins JPT et.al., 2002). Using the criteria, heterogeneity was substantial (I^2>50%) in CDR-SB, ARIA-E, and ARIA-H, moderate in SAE (30%<=I^2<=50%), and mild in ADAS-Cog and other secondary endpoints (I^2<50%). We think the difference in heterogeneity in efficacy endpoints are derived from differences in the criteria in CDR-SB and ADS-Cog. In CDR-SB, the criteria include aspects such as attendance in community affairs, personal care, and home and hobbies. Significant individual differences in the timing of losing these abilities might have led to greater variability across trials. On the other hand, ADAS-Cog focuses on more detailed cognitive abilities, which may allow for a more sensitive assessment of cognitive maintenance or decline. The mild heterogeneity observed with ADAS-Cog suggests that this measure may provide a more reliable assessment of the overall trend in cognitive decline compared to CDR-SB.
For the substantial heterogeneity in ARIA, it is mainly driven by Solanezumab, which uniquely targets monomers and has not demonstrated a biological effect in reducing brain Ab burden. We explained it in the discussion.

Revision

We modified the following sentence in the method section. (Page 9, Line 173).
"Heterogeneity was classified as mild when I^2 was less than 30%, moderate when I^2 ranged from 30% to 50%, and substantial when I^2 exceeded 50% [3]."

We added the following sentences in the discussion section (Page 32, Line 429).
"Substantial heterogeneity was observed in the change in CDR-SB scores, whereas heterogeneity was mild when assessed by ADAS-Cog. In CDR-SB, the criteria include aspects such as attendance in community affairs, personal care, and home and hobbies. Significant individual differences in the timing of losing these abilities might have led to greater variability across trials. On the other hand, ADAS-Cog focuses on more detailed cognitive abilities, which may allow for a more sensitive assessment of cognitive maintenance or decline. The mild heterogeneity observed with ADAS-Cog suggests that this measure may provide a more reliable assessment of the overall trend in cognitive decline compared to CDR-SB."

We added or modified the following sentences in the discussion section.
・page 32, Line 448
"To explore the source of heterogeneity, we performed various meta-regression analyses."

・page 33, Line 459

"ARIA-E and ARIA-H demonstrated substantial heterogeneity, SAE exhibited moderate heterogeneity, and other safety endpoints showed mild heterogeneity across trials."

・page 33, Line 462

"Substantial heterogeneity in ARIA was mainly driven by Solanezumab, which uniquely targets monomers and has not demonstrated a biological effect in reducing brain Aβ burden. "

We added the following reference.

19. Higgins JPT, Thompson SG. Quantifying heterogeneity in a meta-analysis. Statist Med. 2002; 21:1539-1558.

Reviewer Comment 2-8

The NNT/NNH calculations are provided using Kraemer's method, but this approach is known to yield conservative estimates. Although the text mentions an alternative method (Furukawa's), the reasoning behind choosing Kraemer's approach is only briefly stated. Please provide a more in-depth explanation of why Kraemer's method was chosen over other methods for NNT/NNH calculations, despite its conservative estimates. Additionally, including a brief summary of how Furukawa's method might yield different results could contextualize the limitations of using Kraemer's method in this context.

Author Response

Thank you for raising this important point regarding the choice of method for NNT/NNH calculations. Kraemer's method was selected because it provides a consistent and straightforward approach to calculating NNT/NNH using effect size (Cohen's d). Although it tends to yield smaller values, it is widely used and well-documented in meta-analytic studies where individual patient-level data is unavailable. Given the aggregate nature of the data in our study and the lack of access to individual patient-level data, Kraemer's method was the most feasible and reliable approach for our analysis.

To estimate NNT/NNH using Furukawa's method, it would be necessary to determine an appropriate cutoff for the reduction percentage (e.g., 50% reduction). However, selecting such a cutoff can be subjective and introduce potential bias. Additionally, event rates corresponding to this reduction percentage cutoff are required for the calculation, but such data are often not explicitly reported in the literature. Consequently, applying Furukawa's method directly to our dataset would have required making significant assumptions, which might have compromised the validity of the results.

If individual patient-level data had been available, using Furukawa's method to estimate NNT/NNH could have provided values that are more sensitive to the actual distribution of responders in the experimental and control groups. This might have resulted in NNT/NNH estimates that more accurately reflect the clinical reality of the dataset.

Revision

We added the following sentence in the **Text E in S1 Supporting information** (page 15, Line 225).

"Also, it would be necessary to determine an appropriate cutoff for the reduction percentage (e.g., 50% reduction). However, selecting such a cutoff can be subjective and introduce potential bias."

Also, we added the following sentence in the **Text E in S1 Supporting information** (page 19, Line 310).
"If individual patient-level data had been available, using Furukawa's method (Furukawa TA et.al., Plos One 2011) to estimate NNT/NNH could have provided values that are more sensitive to the actual distribution of responders in the experimental and control groups. This might have resulted in NNT/NNH estimates that more accurately reflect the clinical reality of the dataset."

Reviewer Comment 2-9

In some tables, such as Supplemental Tables S2-S5, there are inconsistencies in data presentation (e.g., varying decimal places or differing levels of detail). Please standardize data presentation in all supplemental tables (e.g., using two decimal places throughout or providing consistent detail for each entry).

Author Response

Thank you for your valuable comment.
Except for the following exceptions, data are presented with two decimal places.

- Absolute values < 0.005: reported with three decimal places (e.g., 0.001, -0.002).
- When cases where only one decimal place or integer is reported in the manuscript, data were presented as it was reported.
- The number needed to harm/treat (NNH/NNT) was rounded and reported as an integer, as these measures help readers conceptualize the number of people and are typically presented as integers.
- Baseline characteristics shown in Table 1 are presented with one decimal place to ensure clarity and avoid visual clutter.
- P- Values smaller than 0.001 were reported as P < 0.001, unless only two decimal places were provided in the original source (e.g., Table E, where "P < 0.01" was available from the EXPEDITION 3 trial).

We could not locate specific reporting guidelines provided by the journal. However, we are open to further revisions should such guidelines be available.

For **neuroimaging and biomarker results** (Tables C, D, E, and F in S1 Supporting Information), we included as much detail as possible. Reporting formats varied across trials due to the following reasons:

- Some trials reported only P-values without specific numerical data.
- Others provided mean changes for antibody and placebo groups separately but did not report differences.
- In cases where only line or bar graphs were presented without precise numerical values, we refrained from extracting detailed data, as performing meta-analysis on these outcomes was not the primary objective.

For further clarification, please also refer to our responses to Comment No. 6 and No. 11.

Revision

We have standardized the number formatting in manuscript, **Table1, Table C, D, E and F in S1 Supporting information** to ensure consistency.

Reviewer Comment 2-10
Supplemental Text S3 outlines the methodology for combining groups in cases with multiple doses, yet it could be challenging for readers to follow without more detailed steps or examples. Please provide a step-by-step example of how multiple doses were combined to form a single group for analysis.

Author Response

Thank you for the supportive advice. We added examples for continuous outcomes and categorical outcomes in the **Text S3 in supporting information**. Also, in the previous revision, we provided R script for analysis in the github.

Revision

We added the following examples in the **Text S3 in supporting information** (one example each for a continuous variable and a categorical variable).

Example:

For the Noncarriers study of Bapineuzumab (Salloway S et.al., NEJM 2014), there were two dose groups of Bapineuzumab (0.5mg/kg and 1.0 mg/kg). $N_{combined}$ (the combined sample size of these groups), $M_{combined}$ (the mean change in CDR-SB for the combined group), and $SD_{combined}$ (the standard deviation of the change in CDR-SB for the combined group) were calculated.

$$N_{combined} = N_1 + N_2 = 314 + 307 = 621$$

$$M_{combined} = \frac{N_1 \cdot M_1 + N_2 \cdot M_2}{N_1 + N_2} = \frac{314 \times 2.6 + 307 \times 2.8}{314 + 307} = 2.70$$

$$SD_{combined} = \sqrt{\frac{(\mathrm{N}_1 - 1) \cdot \mathrm{SD}_1^2 + (\mathrm{N}_2 - 1) \cdot \mathrm{SD}_2^2 + \frac{\mathrm{N}_1 \cdot \mathrm{N}_2}{\mathrm{N}_1 + \mathrm{N}_2} \cdot (\mathrm{M}_1 - \mathrm{M}_2)^2}{\mathrm{N}_1 + \mathrm{N}_2 - 1}}$$

$$= \sqrt{\frac{(314 - 1) \times (0.2 \times \sqrt{314})^2 + (307 - 1) \times (0.2 \times \sqrt{307})^2 + \frac{314 \times 307}{314 + 307} \times (2.6 - 2.7)^2}{314 + 307 - 1}}$$

$$= 3.52$$

SD was calculated as $SE \times \sqrt{N}$.

The values were taken from Table 1 and Table 2 of the paper (Salloway S et.al., NEJM 2014).

Example:

For the Noncarriers study of Bapineuzumab (Salloway S et.al., NEJM 2014), there were two dose groups of Bapineuzumab (0.5mg/kg and 1.0 mg/kg). $N_{combined}$ (the combined sample size of these groups), $E_{combined}$ (the combined number of ARIAE events) were calculated.

$$N_{combined} = N_1 + N_2 = 337 + 329 = 666$$

$$E_{combined} = E_1 + E_2 = 14 + 31 = 45$$

The values were taken from and Table 3 of the paper (Salloway S et.al., NEJM 2014)

Reviewer Comment 2-11

The summary of neuroimaging and biomarker results in Supplemental Tables S3 and S4 is helpful, but lacks specific details on study populations, methodologies, or outcome variability, which could affect the interpretation of results. If possible, add details on the neuroimaging methodologies (e.g., PET tracer types) and biomarker assays used across trials, as well as any notable variations in outcomes.

Author Response

We agree with the reviewer that it is important to address the variations among trials regarding the biomarker and neuroimaging methodologies.

Reasons why we did not perform meta-analysis for neuroimaging and biomarker results were already summarized in previous revision, please refer to the section starting from Page 34, Line 488 in the main material.

Revision

We divided the original Supplemental Table C to:
Table C. Summary of amyloid PET
and
Table D. Summary of tau PET.

Also, we divided the original Supplemental Table D to:
Table E: Summary of cerebral fluid biomarker results
and
Table F: Summary of plasma biomarker results

We added additional information in each table to further describe study populations, methodologies (including PET tracer types), or outcome variability.

Reviewer Comment 2-12
While funnel plots and Egger's tests are presented for primary outcomes, a similar assessment for secondary outcomes would strengthen confidence in these findings.

Author Response
Thank you for the important comment. We showed funnel plots and res of Egger’s tests for secondary outcomes in **S2 figure**. In the manuscript, the results are summarized as “There was no evidence of asymmetry in funnel plots in secondary endpoints (**S2 Figure**)”. (Page 16, Line 242).
**Revision**
We did not make any revision in response to this comment.

Responses to the Reviewer #4:

Reviewer Comment 4-1
Thank you for the thorough response and updated data. I have no further comments at this time.

Author Response
Thank you for reviewing our paper.
**Revision**
We did not make any revision in response to this comment.

Responses to the Reviewer #5:

Reviewer Comment 5-1
I appreciate the authors' efforts to address all the comments.

Author Response
We appreciate for the reviewer for having reviewed our paper.
**Revision**
We did not make any revision in response to this comment.
