# Supplementary material for: Efficacy and safety of passive immunotherapies targeting amyloid beta in Alzheimer’s disease: A systematic review and meta-analysis
Source: PLoS Med. 2025 Mar 31;22(3):e1004568. doi: 10.1371/journal.pmed.1004568 (PMC12002640; doi:10.1371/journal.pmed.1004568)
Supplement: Attachment — Submitted filename: Meta_rebuttal_R4.docx [file pmed.1004568.s057.docx]

Point-by-Point Response Letter

**Manuscript #: PMEDICINE-D-24-02295R4**

**Responses to the editors:**

We would like to thank the editors for their variable comments, which has helped us to improve the manuscript. Please find below our point-by point responses to the reviewer’s comments.

**Response to the academic editor**

Comment from the academic editor　1-1

I would suggest they stick to “small” rather than “modest” effects.

Author Response and revision

We agree with the editor and changed the word “modest” to “small” throughout the manuscript.

Comment from the academic editor　1-2

The only area of concern for me is the suggestion that the drug price is somehow cost effective. The NICE analysis suggests even if the , effect size is considered to be meaningful (which is contested), the cost would be miles off what is considered good value, including the challenge of the side effects. The authors mention no effect on cerebral haemorrhage but the ARIA side effects are haemorrhagic so that is a bit misleading.

Author Response

Thank you for pointing these.

In the discussion where we cited medico-economic analyses of lecanemab and donanemab, we clarified that these were conducted in the US. However, they accounted for treatment cost of adverse events such as ARIA when evaluating cost effectiveness. We agree with the editor that cost-effectiveness is not a globally agreed consensus.Therefore, we also described the situation in the UK, citing the draft consultation by NICE, which did not recommend the inclusion of either lecanemab or donanemab in the National Health Service (NHS).

Also, to distinguish ARIA-H (Amyloid-Related Imaging Abnormalities-Hemorrhage, also include microhemorrhage) and cerebral macrohemorrhage (where we define more severe conditions), we sticked to use the word “macrohemorrhage” for the latter condition throughout the manuscript (In the previous version, both “hemorrhage” and “macrohemorrhage” were used. We apologize for the confusion.). For the definition of cerebral macrohemorrhage, please refer to the Text H in the supporting information.

Revision

- We changed the word “cerebral hemorrhage” to “cerebral macrohemorrage” throughout the manuscript.
- We modified the following sentences to clarify that these studies were conducted in the US.

“Reports on the cost-effectiveness of commercially available anti-Aβ antibodies **conducted in the US** suggest that, when compared to standard care without the use of these antibodies, lecanemab and donanemab extend quality-adjusted life years (QALYs) by 0.61 and 0.62 years, respectively [43,44]. While the results of medico-economic effects analysis depend heavily on regional variations in caregiving costs, which calls for caution in interpretation, the price of lecanemab, 26,500 USD per year, is considered to be set lower than its anticipated socioeconomic value **in the US** [43].”

- We added the following sentence.

“In contrast, in the United Kingdom, National Institute for Health and Care Excellence did not recommend the use of either lecanemab or donanemab within the national health service due to their limited clinical benefits and high cost. [45,46]. Thus, the cost-effectiveness of mAb therapy remains controversial, with significant variations in assessment across countries.”

- We cited the following draft guidance consultations for lecanemab and donanemab by NICE.

45.	National Institute for Health and Care Excellence. Draft guidance consultation -Lecanemab for treating mild cognitive impairment or mild dementia caused by Alzheimer's disease. August 2024. [Available from: https://www.nice.org.uk/guidance/gid-ta11220/documents/draft-guidance]. Accessed February 11^th^, 2025.

46.	National Institute for Health and Care Excellence. Draft guidance consultation - Donanemab for treating mild cognitive impairment or mild dementia caused by Alzheimer's disease. October 2024. [Available from: https://www.nice.org.uk/guidance/gid-ta11221/documents/draft-guidance]. Accessed February 11^th^, 2025.

- We consistently used the words”cerebral macromhemorrhage” throughout the manuscript, when we discuss crerebral hemorrhage more serious than ARIA-H or micro hemorrhage. These were highlighted in red.

**Response to the editors**

Comment from the editors　1-1

Title: please modify the title so it complies with PLOS Medicine's style. Your title must be nondeclarative and not a question. It should begin with main concept if possible. "Effect of" should be used only if causality can be inferred, i.e., for an RCT. Please place the study design ("A randomized controlled trial," "A retrospective study," "A modelling study," etc.) in the subtitle (ie, after a colon). We suggest modifying your title to: “Efficacy and safety of passive immunotherapies targeting amyloid beta in Alzheimer’s disease: a systematic review and metaanalysis” (or similar).

Author Response

Thank you for the valuable suggestion. We agree with the editors and modified the title accordingly.

Revision

We changed the title to:

“Efficacy and safety of passive immunotherapies targeting amyloid beta in Alzheimer’s disease: a systematic review and meta-analysis”

Comment from the editors　1-2

Please confirm that your abstract complies with our requirements, including providing all the information relevant to this study type [https://journals.plos.org/plosmedicine/s/submission-guidelines#loc-abstract](https://url.us.m.mimecastprotect.com/s/3UdSCYExODCAZO6zoc3CLTxOz9g?domain=journals.plos.org)

Author Response

We revisited the above-mentioned page to check the requirements for abstract and modified the abstract slightly following the guideline.

Please also refer to the responses to comments 1-3 to 1-7.

Revision

We stated the primary and secondary outcomes clearly in this revision.

“Primary outcomes were mean changes from baseline to post-treatment in Clinical Dementia Rating scale-Sum of Boxes (CDR-SB) and AD Assessment Scale-Cognitive Subscale (ADAS-Cog) scores, and secondary outcomes were adverse events including amyloid related imaging abnormalities with edema (ARIA-E), and ARIA with hemorrhage (ARIA-H).”

Comment from the editors　1-3

Abstract, line 38: please modify “sporadic AD patients” to “patients with sporadic AD” and ensure that you use similar patient-centric language throughout the manuscript (eg, abstract line 47-48 and other instances throughout the paper).

Author Response and revision

We agree with the editor and changed the “sporadic AD patients” to “patients with sporadic AD”.

Comment from the editors　1-4

Abstract, lines 39-40: please provide the beginning and end dates of your search (eg, database inception to Jan 4, 2024?). This should also be added to the methods section.

Author Response

We searched 3 data sources (clinical trials.gov, Pubmed, and Embase) on the same day, January 14^th^, 2024. We further excluded trials using the inclusion and exclusion criteria, but the baseline search was done in one day. If you mean we should also state when we finally decided which trials would be included, it was February 14^th^, 2024.

Revision

Abstract, we modified the following sentences.

“Phase III randomized, placebo-controlled blinded trial evaluating the efficacy and safety of anti-Aβ mAbs in patients with AD were identified through a search in clinical trials.gov, PubMed and Embase on January 14^th^, 2024. The retrieved studies were further screened from January 15^th^, 2024, to February 14^th^, 2024.”

In the method section (Line 134, Page8), we added the following sentence.

“The screening process was conducted between January 15^th^ 2024 and February 14^th^ 2024.”

Comment from the editors　1-5

Abstract, line 47: please include the comparison within the sentence; eg, “Thirteen trials (>18,000 patients) revealed an overall improvement with mAb treatment compared to placebo, with a mean difference in CDR-SB of -0.25 (95%CI [-0.38, -0.11]) (improvement) and a standardized mean difference in ADAS-Cog of -0.09 (95%CI [-0.12, -0.06]).

Author Response

We apologize that the statement was not clear enough. We modified the sentences following your comment.

Revision

We modified the following sentence in the abstract.

“The literature search identified 13 phase III RCTs, which included 18,826 AD patients. Compared with placebo, treatment with mAbs significantly improved cognitive performance on CDR-SB (mean difference [95%CI] = -0.25 [-0.38, -0.11]), and ADAS-Cog (standardized mean difference [95%CI] = -0.09 [-0.12, -0.06]), in which a negative change indicates improvement for both scores.”

Comment from the editors　1-6

Abstract: please include a sentence at the end of the Methods and Findings section indicating the main limitation(s) of the study.

Author Response

We agree with the editor and added some sentences to describe limitations.

Revision

We added the following sentences in the abstract.

“Statistical heterogeneity was relatively high for ARIA-E and ARIA-H, leading to wide confidence intervals and considerable variability in effect sizes, though meta-regression was conducted to address this. Furthermore, differences in trial designs introduce limitations in cross-trial comparisons.”

Comment from the editors　1-7

Abstract: please specify that there were no language restrictions applied to your search.

Author Response

Thank you for a variable advise.

Revision

We added the following sentence in the “Methods and Findings” section of the abstract.

“We included studies that had been published in any language”.

Comment from the editors　1-8

Drug names (donanemab, lecanemab, etc) should not be capitalized; please modify throughout the paper.

Author Response and revision

Thank you for the important advice. We reviewed the manuscript and the supporting information thoroughly and modified the drug names unless they appear at the beginning of a sentence.

Comment from the editors　1-9

Please remove numerical headings and sub-headings (ie, use only text in headings).

Author Response and Revision

Thank you for pointing out the important point. We removed numerical headings and sub-headings throughout the manuscript.

Comment from the editors　1-10

Figures. Please consider avoiding the use of red and green in order to make your figures more accessible.

Author Response and revision

We apologize for not using a color-blind friendly color pallet in the previous version. In this revision, we updated all the figures and the supplemental figures using a color-blind friendly color pallet.

Comment from the editors　1-11

Please resupply the PRISMA checklist using section and paragraph numbers, rather than line numbers.

Author Response and Revision

Thank you for the supportive comment. In this revision, we updated the PRISMA checklist using section and paragraph numbers, rather than pages or line numbers.

**Response to the comments from Reviewers:**

Comment from the Reviewer #2

Thank you for thoroughly addressing all my comments and providing detailed clarifications in your revised manuscript. I appreciate the effort you have put into enhancing the rigor and transparency of your analyses.

The manuscript now reflects a robust and valuable contribution to subject, I am happy to recommend this work for publication and look forward to seeing its positive impact in the field.

Author Response

We appreciate for the reviewer’s variable comments to our manuscript.

Revision

We did not make any revisions for this comment.
