# Supplementary material for: Efficacy and safety of passive immunotherapies targeting amyloid beta in Alzheimer’s disease: A systematic review and meta-analysis
Source: PLoS Med. 2025 Mar 31;22(3):e1004568. doi: 10.1371/journal.pmed.1004568 (PMC12002640; doi:10.1371/journal.pmed.1004568)
Supplement: Attachment — Submitted filename: Meta_Rebuttal_R5.docx [file pmed.1004568.s058.docx]

**Point-by-Point Response Letter**

**Manuscript #: PMEDICINE-D-24-02295R5**

**Responses to the editors:**

We would like to thank the editors for their variable comments, which has helped us to improve the manuscript. Please find below our point-by point responses to the editor’s comments.

Requests from Editors:

1) Abstract: Please define ‘CI’ at first use.

I defined at CI at first use in the abstract.

2) Abstract: “Early-stage AD patients” – please revise with regard to use of patient-centered language (i.e., patients with early-stage AD).

I changed to “patients with early-stage AD”. Also, I slightly modified the sentence to:

“Meta-regression analysis suggested that trials enrolling patients with early-stage AD were associated with better efficacy.”

3) Statistical reporting:
* Please report statistical information as follows to improve clarity for the reader "HR 2.00 (95% CI [1.99,2.01])".
* Please define statistical abbreviations at first use.
* Please repeat statistical definitions (HR, CI etc.) for each set of parentheses.

I changed accordingly. If further modification is needed, please let me know.

4) Author Summary: Please introduce the abbreviation ‘AD’ in the first bullet point or spell out Alzheimer’s disease throughout the Author Summary.

I introduced the abbreviation “AD” in the first bullet point of the author summary.

5) Author Summary: Please change ‘AD patients’ to ‘patients with AD’.

I changed accordingly.

6) Author Summary: In the final bullet point of 'What Do These Findings Mean?', please include the main limitations of the study in non-technical language.

I added the following bullet point at the last of the author summary.

- One limitation of the study is that the trials were designed differently, making it hard to directly compare their results. Also, we did not have detailed data on individual patients.

7) Results: On line 228, please change ‘AD patients’ to ‘patients with AD’.

Thank you for the supportive comment. I modified to “18,826 patients with MCI or dementia due to AD”.

8) Table 2: “only the mild AD patients were included” – please change to: “only patients with mild AD were included”.

I changed accordingly.

9) Table 3/Table 4: Please define ‘Ab’, ‘PET’, ‘NA’, ‘CI’ below the table.

I added definitions for the abbreviations below the table. For “Ab”, I changed the font to “Aβ”.

10) Discussion: On lines 467 and 468, please change ‘early AD patients’ to ‘patients with early AD’.

I changed accordingly.

11) Figure 1: Please define ‘AD’ and RCT’ in the figure description.

I added the definitions for AD and RCT in the figure legend. Also, I changed the “AD patients” to “patients with AD” in the figure.

12) Figure 5: Please define ‘RR’ and ‘CI’ in the figure description.

I added the definitions for RR and CI in the figure legend.
